# Design, Synthesis, and Evaluation of 1-Benzylpiperidine and 1-Benzoylpiperidine Derivatives as Dual-Target Inhibitors of Acetylcholinesterase and Serotonin Transporter for Alzheimer′s Disease †

**DOI:** 10.3390/molecules30143047

**Published:** 2025-07-21

**Authors:** Juan Pablo González-Gutiérrez, Damián Castillo-Ríos, Víctor Ríos-Campos, Ignacio Alejandro González-Gutiérrez, Dánae Flores Melivilu, Emilio Hormazábal Uribe, Felipe Moraga-Nicolás, Kerim Segura, Valentina Hernández, Amaury Farías-Cea, Hernán Armando Pessoa-Mahana, Miguel Iván Reyes-Parada, Patricio Iturriaga-Vásquez

**Affiliations:** 1 Instituto de Ciencias Aplicadas, Facultad de Ingeniería, Universidad Autónoma de Chile, Talca 3467987, Chile; damian.castillo@cloud.uautonoma.cl (D.C.-R.); miguel.reyes@usach.cl (M.I.R.-P.); 2 Electrical Signaling in Plants (ESP) Laboratory, Center of Bioinformatics, Simulation and Modeling (CBSM), Faculty of Engineering, Universidad de Talca, Campus Talca, Avenida Lircay, Talca 3460000, Chile; victoroibt@gmail.com; 3 Interno de Medicina, Facultad de Medicina y Ciencias de la Salud, Universidad Mayor, Huechuraba 8580000, Chile; ignacio.gonzalezgu@mayor.cl; 4 Carrera de Bioquímica, Departamento de Ciencias Químicas y Recursos Naturales, Facultad de Ingeniería y Ciencias, Universidad de La Frontera, Temuco 4811230, Chile; d.flores06@ufromail.cl; 5 Laboratorio de Química Ecológica, Departamento de Ciencias Químicas y Recursos Naturales, Universidad de La Frontera, Temuco 4811230, Chile; emilio.hormazabal@ufrontera.cl (E.H.U.); felipe.moraga@ufrontera.cl (F.M.-N.); 6 Centro de Excelencia de Investigación Biotecnológica Aplicada al Medio Ambiente (CIBAMA), Universidad de La Frontera, Temuco 4811230, Chile; 7 Laboratorio de Farmacología Molecular y Química Medicinal, Facultad de Ingeniería y Ciencias, Universidad de La Frontera, Temuco 4811230, Chile; k.segura01@ufromail.cl (K.S.); v.hernandez06@ufromail.cl (V.H.); 8 Laboratorio de Bioquímica y Farmacología Molecular, Escuela de Ciencias, Facultad de Ciencias de la Vida, Universidad Viña del Mar, Viña del Mar 2572007, Chile; amaury.farias@uvm.cl; 9 Escuela de Educación, Facultad de Ciencias Jurídicas, Sociales y de la Educación, Universidad Viña del Mar, Viña del Mar 2580022, Chile; 10 Departamento de Química Orgánica y Fisicoquímica, Facultad de Ciencias Químicas y Farmacéuticas, Universidad de Chile, Santiago 8380492, Chile; hpessoa@ciq.uchile.cl; 11 Centro de Investigación Biomédica y Aplicada (CIBAP), Escuela de Medicina, Facultad de Ciencias Médicas, Universidad de Santiago de Chile, Santiago 9170022, Chile

**Keywords:** organic synthesis, AChE inhibitor, SERT

## Abstract

Cholinergic neuron impairment is a significant cause of cognitive decline in Alzheimer’s disease (AD), making acetylcholinesterase (AChE) a key therapeutic target. AChE inhibitors are principal drugs prescribed to alleviate symptoms in AD patients, while up to 50% of these individuals also suffer from depression, frequently treated with selective serotonin reuptake inhibitors (SSRIs). Due to the multisymptomatic nature of AD, there is a growing interest in developing multitargeted ligands that simultaneously enhance cholinergic and serotonergic tone. This study presents the synthesis of novel ligands based on functionalized piperidines, evaluated through radioligand binding assays at the serotonin transporter (SERT) and AChE and butyrylcholinesterase (BuChE) inhibition. The pharmacological results showed that some compounds exhibited moderate inhibitory activity against AChE, with one compound **19** standing out as the most potent, also displaying a moderate BuChE inhibitory activity, while showing low affinity for SERT. On the other hand, compound **21** displayed an interesting polypharmacological profile, with good and selective activity against BuChE and SERT. The results underscore the difficulty of designing promiscuous ligands for these targets and suggest that future structural modifications could optimize their therapeutic potential in AD.

## 1. Introduction

Alzheimer’s disease (AD) is an irreversible neurological disorder common in elderly patients that affects a large portion of the world’s population [1]. Clinically, it is characterized by the onset of progressive cognitive impairments, leading to memory loss and diminished learning ability, along with a reduced capacity to perform routine daily activities and a variety of adverse neuropsychiatric symptoms such as apathy, verbal and physical agitation, irritability, anxiety, depression, delusions, and hallucinations [2]. The early pathogenic steps of this neurodegenerative disease are associated with the formation of β-amyloid plaques (amyloid cascade hypothesis) [3]. The elements of the amyloid cascade include local inflammation, oxidation, excitotoxicity due to excessive levels of glutamate, and hyperphosphorylation of the Tau protein. As a result of this process, hyperphosphorylated Tau proteins fold into intraneuronal tangles, leading to cell death [4]. Progressive neuronal destruction leads to scarcity and imbalance among various neurotransmitters (e.g., acetylcholine, dopamine, serotonin) and the cognitive deficiencies observed in AD [3,5]. Its treatment aims to interfere with these steps to block the course of the disease in its early stages. In this context, in the exploration of new therapeutic approaches for AD, mechanisms targeting acetylcholine-mediated processes (“cholinergic hypothesis”) have been primarily pursued in the search for new drugs to alleviate the progressive effects of this disease [6]. According to this hypothesis, the hydrolysis of acetylcholine (ACh) creates a deficiency of this neurotransmitter in the brain, which has been regarded as one of the leading causes of AD [7].

**Figure 1:**
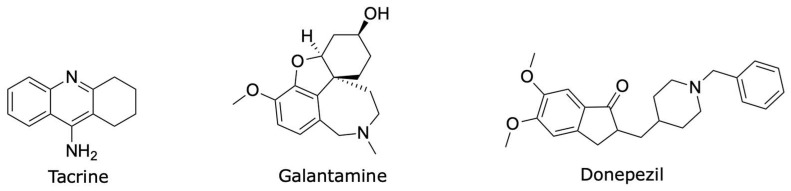
Tacrine, galantamine, and donepezil—AChE inhibitors—are drugs used for the treatment of AD.

ACh is the endogenous ligand of cholinergic receptors, widely distributed in the central (CNS) and peripheral nervous systems [8]. Cholinergic activity is key for cerebral cortex operation and plays a crucial role in memory processes and selective attention [9,10]. ACh is synthesized from choline and acetyl-CoA by the enzyme choline acetyltransferase and stored in synaptic vesicles for subsequent release. Once ACh is released into the synaptic cleft [11] it can bind to its cholinergic receptors on the postsynaptic neuron, promoting the propagation of the nerve impulse, or it can be hydrolyzed by the enzyme acetylcholinesterase (AChE), terminating the cholinergic impulse [11]. AChE is a relevant enzyme involved in cholinergic neurotransmission, both in the peripheral and central nervous systems. As mentioned earlier, its primary function is to catalyze the hydrolysis of ACh to generate choline and acetate ions [12]. AChE is an important therapeutic target for treating AD [13] because the deterioration of cholinergic neurons in the brain and the loss of cholinergic neurotransmission due to ACh degradation are among the leading causes of cognitive decline in patients with AD [13]. Current first-line treatments for mild and moderate AD include the use of AChE inhibitors such as tacrine (IC_50_ = 0.424 μM in human AChE; h-AChE), galantamine (IC_50_ = 18.6 μM in h-AChE), and donepezil (IC_50_ = 0.023 μM in h-AChE) [14,15,16] (Figure 1), which consequently increases cholinergic tone in the mesocortical pathway [17,18]. However, these drugs are unable to slow or prevent the progression of AD, and they only provide symptomatic benefits and lose therapeutic efficacy over time [19].

**Figure 2:**
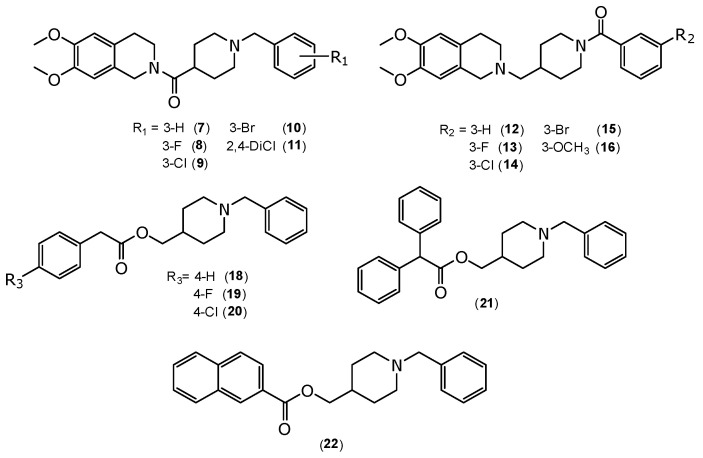
Chemical structure of 1,2,3,4-tetrahydroisoquinolines 1-benzylpiperidine or 1-benzoylpiperidine functionalized derivatives (compounds **7**–**16**) and chemical structure of phenylacetate and 2-naphthoate derivatives of 1-benzylpiperidine (compounds **18**–**22**).

Although progressive cognitive decline is the hallmark of the disease, neuropsychiatric symptoms affect nearly all patients and are often persistent [17]. In addition, depression is one of the most common neuropsychiatric symptoms in Alzheimer’s disease, affecting up to 50% of patients. Antidepressants, such as selective serotonin reuptake inhibitors (SSRIs), remain as one of the first-line treatments for Alzheimer’s patients suffering from depression [18]. Currently, due to the limited efficacy of AChE inhibitors as standalone treatments, there is a growing demand for new therapeutic options to address this condition [20] as well as a lack of alternative treatment options for patients with depression [18]. Considering the multisymptomatological nature of AD, an alternative strategy to the use of combinations of drugs is the design of novel multitarget ligands that simultaneously enhance cholinergic and serotonergic tone by inhibiting AChE and blocking the serotonin transporter, respectively [20]. Based on the aforementioned factors and on the versatility of piperidine as a common scaffold for AChE or monoamine transporters ligands [21,22], we report here the synthesis of 1,2,3,4-tetrahydroisoquinolines-1-benzoylpiperidine or -1-benzylpiperidine functionalized derivatives (Figure 2, compounds **7**–**16**) and functionalized 2-phenylacetate, 2,2-diphenylacetate, and 2-naphthoate derivatives of 1-benzylpiperidine (Figure 2, compounds **18**–**22**). These compounds were evaluated for their inhibitory activity on AChE and their affinity for the serotonin transporter (SERT), aiming to potentially address both cognitive deficits and neuropsychiatric symptoms in AD.

## 2. Results and Discussion

### 2.1. Chemistry

**Figure 3:**
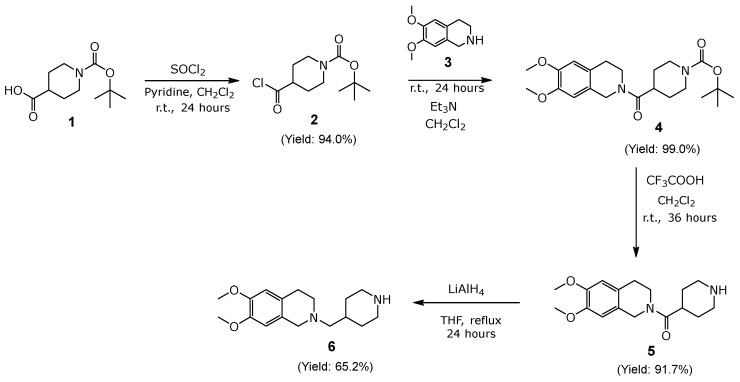
Synthetic Scheme I of compounds (**2**, **4**, **5**, and **6**).

For the synthesis of compound **2**, as described in Section 3.1.1 (Figure 3), ethyl ether was added to precipitate pyridine as pyridinium salt (a white solid), which could be separated from the reaction crude by filtration. Compound **2** showed a good reaction yield (94.0%), and due to the reactivity of compound **2**, it was used immediately for the following reaction.

For the synthesis of compound **4**, as described in Section 3.1.2 (Figure 3), the reaction between compound **2** and 6,7-dimethoxy-1,2,3,4-tetrahydroisoquinoline (compound **3,** Figure 3) yields compound **4** with a yield of 99.0%. The reaction was monitored by TLC using ethyl acetate as the mobile phase. This compound was characterized by ^1^H-NMR, showing the following characteristic signals: a signal at δ 6.73 ppm indicating the presence of two aromatic protons, a signal at δ 3.71 ppm originating from the CH_3_O- and CH_2_-N groups of piperidine, and a signal at δ 1.40 ppm corresponding to the three CH_3_ groups of N-Boc-piperidine. Compound **4** was used immediately for the following reaction without further purification.

Compound **5** was synthesized according to the procedure described in Section 3.1.3 (Figure 3), with a yield of 91.7%. The characterization of this compound was performed using ^1^H-NMR, showing the following characteristic signals: a signal at δ 6.59 ppm originating from the aromatic protons of 1,2,3,4-tetrahydroisoquinolines (THQ) and a signal at δ 2.02 ppm corresponding to the three aliphatic -NH- groups of piperidine after the removal of the N-Boc protecting group. Compound **6** was synthesized according to Section 3.1.4 (Figure 3), with a reaction yield of 65.2%, without further purification. The reaction was monitored by TLC using methanol as the mobile phase. This compound was characterized by using ^1^H-NMR, obtaining the following characteristic signals: two aromatic signals at δ 6.56 ppm and 6.49 ppm, each integrating for one hydrogen, a signal at δ 3.80 ppm integrating for *six* hydrogens corresponding to the -OCH_3_ of the methoxy groups of THQ, and a signal integrating for two hydrogens at δ 2.31 ppm.

**Figure 4:**
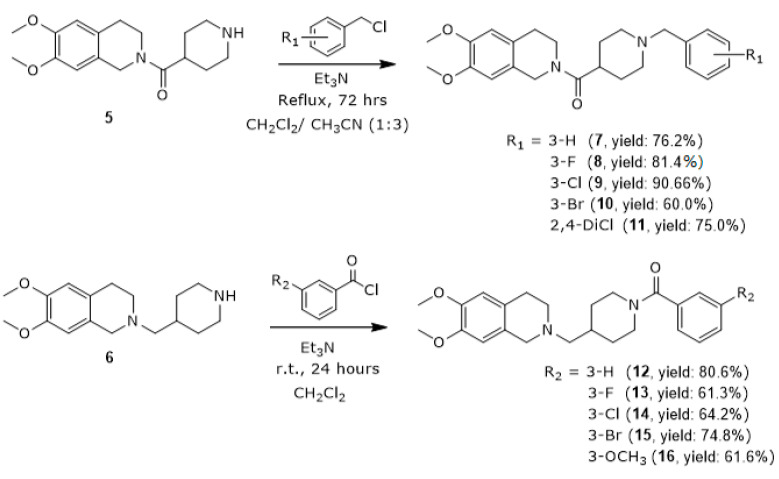
Synthetic Scheme II of THQ compounds (**7**–**16**).

**Figure 5:**
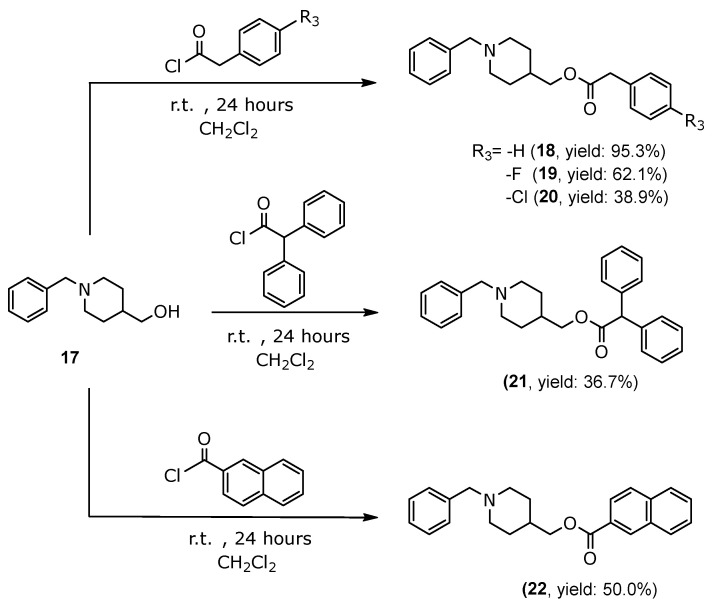
Synthetic Scheme III of compounds (**18**–**22**).

Compounds (**7**–**16**) were synthesized according to Section 3.1.5 and Section 3.1.6 (Figure 4), and compounds (**18**–**22**) were synthesized according to Section 3.1.7 (Figure 5), with yields ranging from 36.7% to 95.3%. All the synthesized compounds (**7**–**16** and **18**–**22**), which were fully characterized spectroscopically by FT-IR, ^1^H-NMR, ^13^C-NMR, and HRMS were observed to exhibit the following signals:

In ^1^H-NMR, all compounds (**7**–**16**) exhibited an aromatic signal from the phenyl group with a δ range of 7.90 to 7.24 ppm, which, depending on substitution, integrates for five to three hydrogens. Additionally, an aromatic signal was observed with a δ range of 6.98 to 6.79 ppm, integrating for two hydrogens from the THQ, and a signal integrating for six hydrogens with a δ range of 3.90 to 3.74 ppm, originating from the methoxy groups of the THQ.

Regarding compounds (**7**–**11**), they showed a signal at δ 3.60 ppm, integrating for two hydrogens from the methylene group of the alkyl chain between the piperidine and the phenyl group (N-CH_2_-Ph). On the other hand, compounds (**12**–**16**) presented signals with a δ ranging from 3.19 to 3.01 ppm, originating from the methylene of the alkyl chain located between the amino group of the THQ and the methylene of the piperidine (N-CH_2_-CH). The compounds (**18**–**22**) exhibited an aromatic signal from the phenyl groups, with δ values ranging from 8.26 to 7.08 ppm, which, depending on substitution, integrated for 15 to 10 hydrogens. Additionally, they showed a signal with a δ ranging from δ 4.28 to 3.85 ppm, integrating for two hydrogens from the methylene of the alkyl chain between piperidine and the phenyl group (N-CH_2_-Ph). Finally, these compounds (**18**–**22**) presented signals with a δ ranging from 4.03 to 3.62 ppm, originating from the methylene of the alkyl chain located between the oxygen from the ester group (phenylacetate moiety) and the methylene of the ester group (COO-CH_2_-CH).

On the other hand, in ^13^C-NMR, compounds (**7**–**16**) exhibited the following characteristics: a signal in a range of δ 173.0 to 168.0 ppm, which corresponds to the carbonyl carbon of the amide; twelve signals in a range of δ 159.0 to 109.0 ppm, which arise from the aromatic carbons; and one signal in a range of δ 60.0 to 57.0 ppm that originates from the methylene group. In the case of molecules (**7**–**11**), this methylene group is part of the N-CH_2_-Ph portion, while in the case of molecules (**12**–**16**), it comes from the methylene group of the N-CH_2_-CH portion. Additionally, these molecules exhibit a signal from the methoxy (CH_3_O-) carbon groups of the THQ at a range of δ 56.0 to 55.0 ppm. Compounds (**18**–**22**) exhibit a signal in a range of δ 174.56 to 166.59 ppm, which corresponds to the carbonyl carbon of the ester; signals in a range of δ 163.04 to 115.31 ppm arising from the aromatic carbons; and one signal in a range of δ 63.19 to 60.67 ppm that originates from the methylene group, which is part of the N-CH_2_-Ph portion. Additionally, these molecules exhibit a signal from the methylene group as part of the COO-CH_2_ (ester group) at a range of δ 69.37 to 65.20 ppm (all spectra figures are added in the Supporting Information 1-D spectra Figures S1–S33 and 2-D spectra HSQC and HMQC Figures S49–S52).

Furthermore, in IR spectroscopy, compounds (**7**–**16**) exhibited the following characteristics: an aromatic C-H absorption stretching band between 3030 and 3010 cm^−1^, an aliphatic C-H absorption stretching band between 2940 and 2930 cm^−1^, an absorption stretching band for the methoxy groups between 2830 and 2810 cm^−1^, an absorption stretching band for the amide carbonyl group between 1640 and 1610 cm^−1^, a C-O absorption stretching band between 1230 and 1210 cm^−1^, and an absorption stretching band for C-N between 1120 and 1100 cm^−1^. Compounds (**18**–**22**) exhibited the following characteristics: an aromatic C-H absorption stretching band between 3047 and 3025 cm^−1^, an aliphatic C-H absorption stretching band between 2941 and 2886 cm^−1^, an absorption stretching band for the ester carbonyl group between 1735 and 1707 cm^−1^, a C-O absorption stretching band between 1255 and 1218 cm^−1^, and an absorption stretching band for C-N between 1147 and 1040 cm^−1^.

For high-resolution mass spectrometry (HRMS), the molar masses found by this technique for all compounds were compared with those calculated theoretically (all HRMS figures are shown in Supporting Information Figures S34–S48).

### 2.2. Biological Evaluation

To evaluate the affinity of our compounds for h-SERT, binding affinity (K_i_) was determined using [^3^H]-paroxetine ([^3^H]-parox) as a specific radioligand (Section 3.4). Competitive binding studies were conducted on homogenized membranes prepared from the human clonal cell line HEK293 for h-SERT. The estimated K_i_ values indicate that some compounds display competitive binding affinity for h-SERT. The inhibitory activity on AChE or butyrylcholinesterase (BuChE), which has also been regarded as a relevant cholinesterase target in AD [23], was determined using the spectrophotometric method, as described by Ellman, modified from Mella (Section 3.3). The IC_50_ values indicate that some compounds inhibit AChE and/or BuChE. The results of the biological evaluation of our compounds on AChE, BuChE, and h-SERT are shown in Table 1 (the corresponding concentration–response curves are provided in Supplementary Information Figures S53 and S54).

Compounds **7**–**10** exhibited moderate inhibitory activity against AChE (IC_50_ values between 28 and 41 µM) and a low affinity for both BuChE and SERT. The presence of halogen substituents at position 3 of the aromatic ring in the benzylpiperidine moiety (as in compounds **8**–**10**) did not enhance activity towards AChE nor promote interaction with SERT. In contrast, compounds **12**–**16** displayed an affinity for SERT in the low micromolar range but exhibited significantly lower (or negligible) inhibitory activity against AChE (IC_50_ values > 200 µM) and BuChE. This indicates that, in the series studied here, the structural features favoring SERT binding are distinct from those that optimize AChE inhibition.

Compound **19** emerged as the most potent AChE inhibitor in the series (IC_50_ = 5.10 ± 0.24 µM). This could be attributed to the presence of a fluorine atom at the *para* position in the 2-phenylacetate moiety, which likely enhances interaction with key residues at the AChE active site. However, this compound was less potent than the reference drug galantamine (IC_50_ = 1.19 ± 0.046 µM). Interestingly, compound **19** also exhibited a moderate inhibitory activity against BuChE (IC_50_ = 26.78 ± 0.81 µM), suggesting a dual inhibitory profile that, as in galantamine, could be therapeutically relevant. Despite its effectiveness against cholinesterases, its affinity for h-SERT was notably low (K_i_ = 196.6 ± 11.34 µM). This finding indicates that incorporating bulky aromatic substituents may favor interaction with AChE and BuChE but negatively impact binding to h-SERT, possibly due to steric hindrance.

On the other hand, compound **21** displayed a good inhibitory activity against BuChE (IC_50_ = 6.16 ± 0.29 µM), which was more potent and selective (considering the effect upon AChE IC_50_ > 500 µM) than that of galanthamine. In addition, although lower, the affinity of compound **21** for SERT (K_i_ = 25.5 ± 1.01 µM) was in the same range as that shown for BuChE. Thus, compound **21** appears as an interesting lead for drugs with a novel and potentially relevant polypharmacological profile. Furthermore, these results suggest that while structural modifications introducing steric bulk on the benzylpiperidine moiety may enhance selectivity towards BuChE, they may also have less effect on SERT affinity, favoring the appearance of an attractive dual-target activity. Overall, compound **19** stands out as the only molecule in the series with dual inhibitory activity against both AChE and BuChE, whereas compound **21** appears to be a promising selective BuChE inhibitor with an additional activity upon SERT.

### 2.3. Molecular Docking

**Figure 6:**
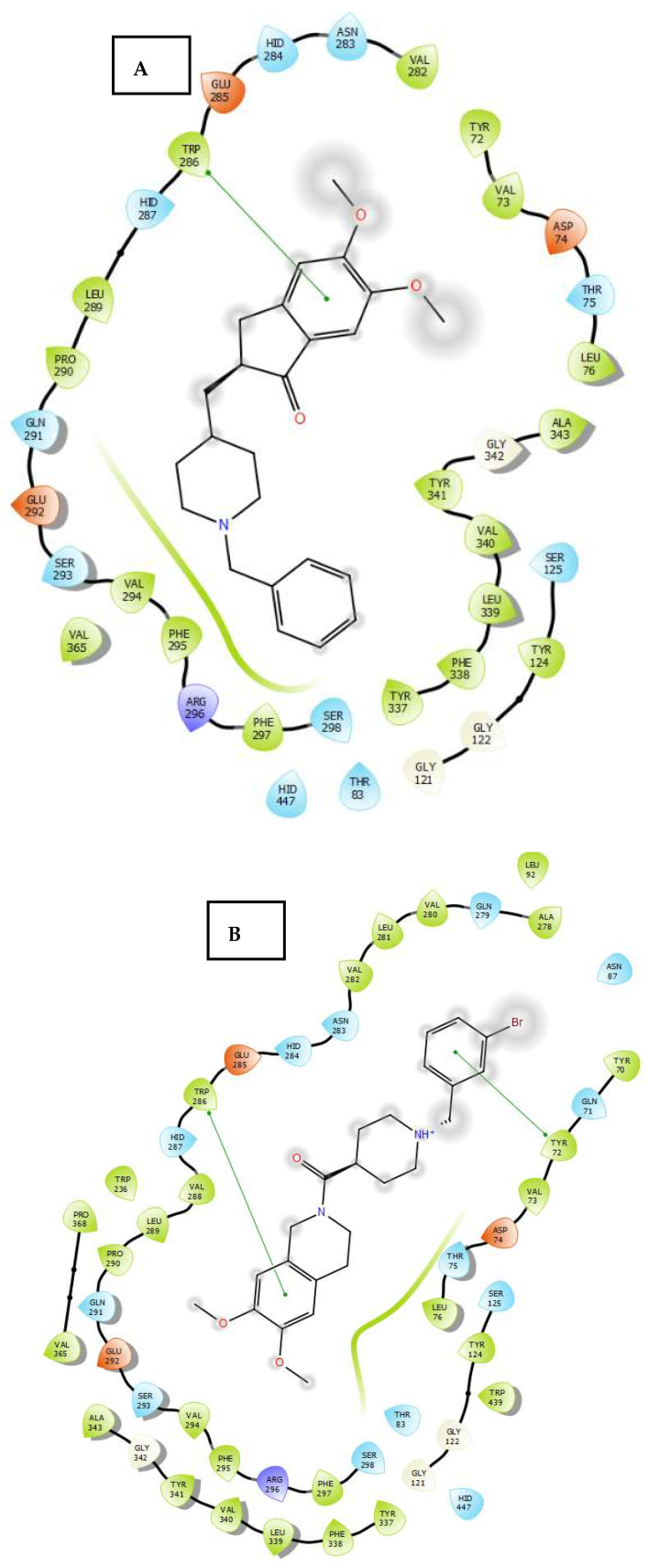

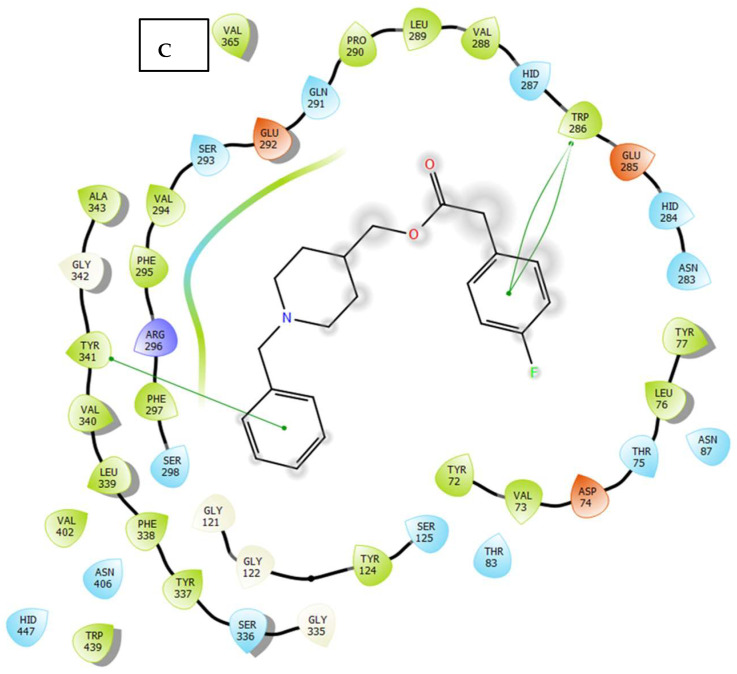
Binding modes of donepezil (**A**), compound **10** (**B**), and compound **19** (**C**) to human AChE (PDB: 1B41). Dotted lines correspond to different interactions. Dotted lines represent various interactions that may occur in ligand–enzyme complexes.

To evaluate how ligands **7**–**22** interact with the enzyme, molecular docking simulations were performed as described in Section 3.4. The X-ray crystal structures of human AChE and SERT were obtained from the Protein Data Bank (PDB: 1B41 and 6AWN, respectively). The docking studies of the compounds with AChE are in line with the experimental results and reveal the following interactions: compounds **7**, **8**, and **10** (Figure 6B) exhibited π-π interactions between the aromatic ring of the 1,2,3,4-tetrahydroisoquinoline moiety of the ligand and the residue Trp286 at the catalytic site of the enzyme. Additionally, compounds **7**–**11** exhibited a π-π interaction between the aromatic ring of the N-benzylpiperidine moiety of the ligand and the Tyr341, Tyr337, and Tyr72 residues. Compound **9** was located in a position that suggested π-π interactions between the aromatic ring of the 1,2,3,4-tetrahydroisoquinoline moiety of the ligand and the Trp286 and Tyr72 residues, as well as a π-π interaction between the aromatic ring of the N-benzylpiperidine moiety of the ligand and the Tyr341 residue. Compound **16** showed a π-π interaction between the aromatic ring of the 1,2,3,4-tetrahydroisoquinoline moiety of the ligand and the Trp286 residue, as well as a π-π interaction between the aromatic ring of the *N*-benzylpiperidine moiety of the ligand and the Tyr72 residue. Compound **18** exhibited π-π interactions between the aromatic ring of its 2-phenylacetate moiety and the Trp286 and Tyr72 residues at the enzyme’s active site. Additionally, a π-π interaction was apparent between the aromatic ring of the ligand’s *N*-benzylpiperidine moiety and the Tyr341 residue. The docking studies for compound **19** (Figure 6C) are consistent with the biochemical results, and the high affinity observed may be explained by the presence of a double π-π interaction between the aromatic ring of its 2-phenylacetate moiety and the Trp286 residue, as well as a π-π interaction between the aromatic ring of its N-benzylpiperidine moiety and the Tyr341 residue. These interactions enhance the stability of the ligand–enzyme complex. Notably, these interactions are absent in donepezil (Figure 6A). Similarly, compound **20** exhibited π-π interactions between the aromatic ring of its 2-phenylacetate moiety and the Trp286 residue, as well as with the Tyr341 residue via the *N*-benzylpiperidine moiety. Compound **21** exhibited a π-π interaction between the aromatic ring of its 2-phenylacetate moiety and the Tyr121 residue. Finally, compound **22** exhibited π-π interactions between the aromatic ring of its naphthoate moiety and the Trp286 and Tyr341 residues.

**Figure 7:**
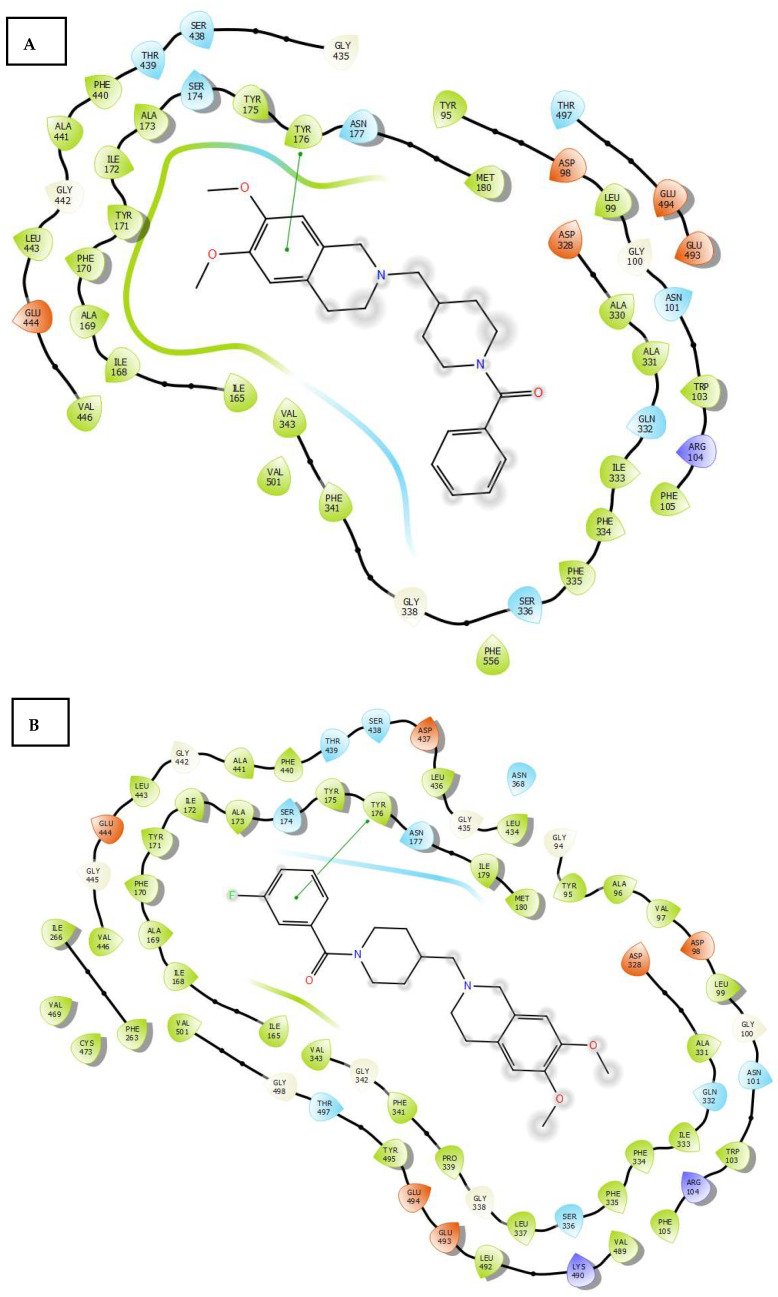

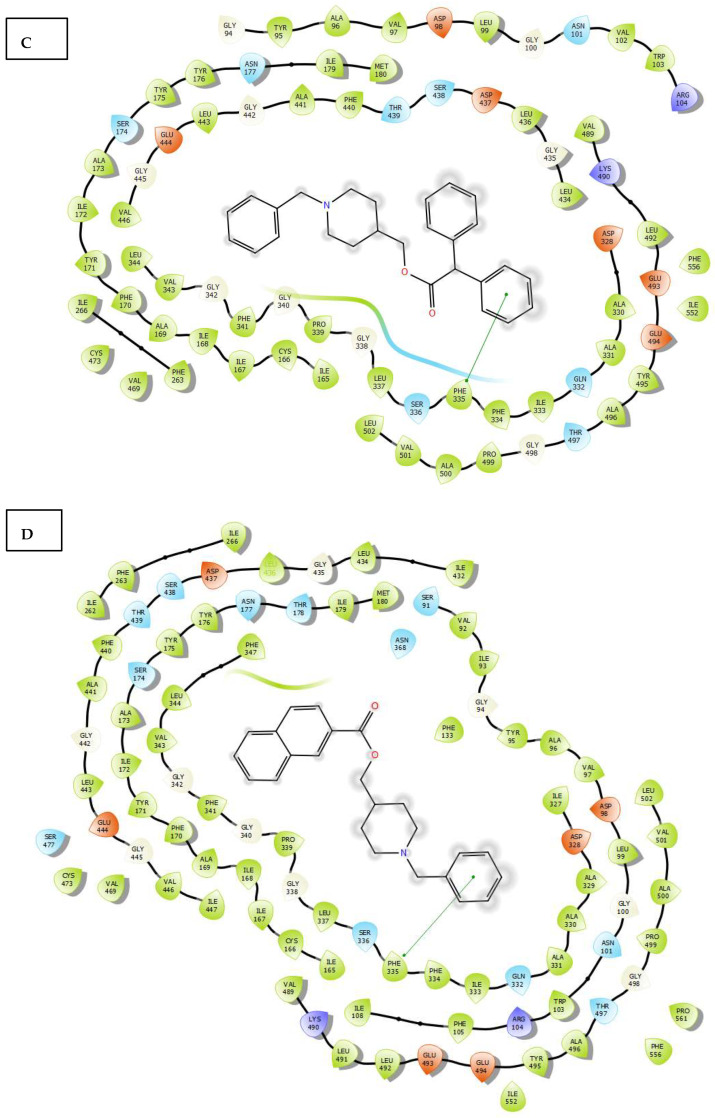
Binding modes of compound **12** (**A**), compound **13** (**B**), compound **21** (**C**), and compound **22** (**D**) to human h-SERT (PDB: 6AWN). Dotted lines correspond to different interactions. Dotted lines correspond to different interactions which may take place in ligand-enzyme complexes.

The docking studies of the compounds on h-SERT are in line with the experimental results and reveal the following interactions: Unsubstituted compound **12** (Figure 7A) exhibits π-π interactions between the aromatic ring of its 1,2,3,4-tetrahydroisoquinoline moiety and the Tyr176 residue. In comparison, compounds **13** (Figure 7B) and **14**, with similar K_i_ values, exhibit π-π interactions between the aromatic ring of their *N*-benzylpiperidine moiety and the Tyr176 residue at the central site. For compound **16**, a notable cation–π interaction was observed between the 1,2,3,4-tetrahydroisoquinoline moiety of the ligand and the Arg104 residue. The poor affinity of these compounds (**12**-**16**) for the central site of h-SERT could be explained by the absence of ionic and/or cation–π interactions between the amine of the piperidine ring or 1,2,3,4-tetrahydroisoquinoline moiety and an Asp or Glu residue at binding site, which are commonly observed between known ligands like paroxetine and the central site of h-SERT [24]. This suggests that the addition of a benzyl group to the piperidine ring would hinder the formation of this cation–π interaction, thereby increasing the energy of the ligand–protein complex and its affinity. The docking studies of the compounds on h-SERT also revealed the following interactions: Compound **19** exhibited double π-π interactions between the aromatic ring of its 2-phenylacetate moiety and the Phe335 and Phe341 residues at the central site. In the case of compound **21** (Figure 7C), a π-π interaction occurred between one of the aromatic rings of the 2-phenylacetate moiety and the Phe335 residue at the central site of h-SERT. In comparison, compound **22** (Figure 7D) showed a π-π interaction between the aromatic ring of its *N*-benzylpiperidine moiety and the Phe335 residue. The relative lower affinity of compounds **19**, **21**, and **22** (as compared with compounds **12**–**16)** for the central site of h-SERT could be explained by the absence of cation–π interactions between the amine of the piperidine ring and an Asp or Glu residue, which are commonly observed between known ligands like paroxetine and the central site of h-SERT [24]. This suggests that the addition of a benzyl group to the piperidine ring would hinder the formation of this cation–π interaction, thereby decreasing affinity.

## 3. Materials and Methods

### 3.1. Synthesis and Chemical Characterization

All the reagents and solvents used to synthesize the compounds were obtained commercially and used without further purification. The 6,7-dimethoxy-1,2,3,4-tetrahydroisoquinoline hydrochloride, *N*-Boc-Isonipecotic acid, benzyl alcohol derivatives, benzoyl chloride derivatives (1-benzylpiperidin-4-yl)methanol (1-benzylpiperidin-4-yl)methanol, 2,2-diphenylacetyl chloride, 2-naphthoyl chloride, 2-naphthoyl chloride, and 2-phenylacetyl chloride derivatives were obtained from Ak Scientific Inc. (Union City, CA, USA), and thionyl chloride, acetone, diethyl ether, dichloromethane, methanol, ethyl acetate, and isopropyl alcohol were obtained from Merck Millipore (Darmstadt, Germany).

The IR spectra were recorded on an FT-IR IRSpirit Shimadzu (Kyoto, Japan), and wavenumbers were reported in cm^−^^1^. ^1^H-NMR, ^13^C-NMR, HSQC, and HMQC spectra were recorded using a Bruker AMX 400 spectrometer (Billerica, MA, USA) at 400 MHz (all spectra are shown in the Supporting Information; 1-D spectra Figures S1–S33 and 2-D spectra HSQC and HMQC Figures S49–S52*)*. Chemical shifts were reported relative to TMS (δ = 0.00) and coupling constants (*J*) are given in Hz. High-resolution mass spectra (HRMS) were recorded using a Bruker compact QTOF MS with direct injection. Reactions and product mixtures were routinely monitored by thin-layer chromatography (TLC) on silica-gel-pre-coated F_254_ Merck plates (Darmstadt, Germany), and the compounds obtained were purified by column chromatography using ethyl acetate as the mobile phase.

#### 3.1.1. Procedures for the Synthesis of *tert*-Butyl-4-(chlorocarbonyl)piperidine-1-carboxylate (**2**)

The *N*-Boc-isopiperic acid (compound **1**) (4.3 mmol, 1.0 g) was reacted with pyridine (4.3 mmol, 0.35 mL) and SOCl_2_ (4.3 mmol, 0.3 mL) in 50 mL of CH_2_Cl_2_ at room temperature with constant stirring for 24 h. After the reaction was completed, the crude reaction mixture was concentrated under reduced pressure, and then diethyl ether was added, resulting in the formation of a white precipitate, which was filtered and discarded. The product dissolved in diethyl ether was concentrated under reduced pressure and used without further purification for the next reaction. Yield 94.0%.

#### 3.1.2. Procedures for the Synthesis of *tert*-Butyl-4-(6,7-dimethoxy-1,2,3,4-tetrahydroisoquinoline-2-carbonyl)piperidine-1-carboxylate (**4**)

The previous *tert*-butyl-4-(chlorocarbonyl)piperidine-1-carboxylate (compound **2**, 8.07 mmol, 2.0 g) was reacted with 6,7-dimethoxy-1,2,3,4-tetrahydroisoquinoline (compound **3**, 8.07 mmol, 1.56 g) and a spoonful of NaHCO_3_ in 50 mL of CH_2_Cl_2_ at room temperature with constant stirring for 24 h. Then, a 10% NaHCO_3_ solution was added to the reaction mixture and transferred to a separatory funnel. The compound was extracted using 3 portions of 30 mL of CH_2_Cl_2_, and anhydrous sodium sulfate was added to the organic phase. Finally, the solution was filtered and concentrated under reduced pressure, resulting in the formation of a white solid that was used for the next reaction without further purification. Yield 99.0%. (C_22_H_32_N_2_O_5_): ^1^H-NMR (400 MHz, DMSO-d_6_) δ 6.61 (s, 2H), 4.64 (s, 1H), 4.59 (s, 1H), 4.15 (br, 2H), 3.84 (s, 7H), 3.71 (s, 1H), 2.79 (m, 5H), 1.72 (m, 4H), 1.45 (s, 9H).

#### 3.1.3. Procedures for the Synthesis of (6,7-Dimethoxy-3,4-dihydroisoquinolin-2(1*H*)-yl)(piperidin-4-yl)methanone (**5**)

The *tert*-butyl-4-(6,7-dimethoxy-1,2,3,4-tetrahydroisoquinoline-2-carbonyl)piperidine-1-carboxylate (compound **4**, 4.95 mmol, 2.0 g) was reacted with 5 mL of trifluoroacetic acid in 100 mL of CH_2_Cl_2_ at room temperature with constant stirring for 24 h. After the reaction time concluded, the mixture was neutralized using a concentrated 10% K_2_CO_3_ solution. The biphasic solution was then transferred to a separatory funnel, and the organic compound was extracted with five 30 mL portions of CH_2_Cl_2_. The organic phase was concentrated under reduced pressure, yielding a white solid with a 91.7% yield. (C_17_H_24_N_2_O_3_): ^1^H-NMR (400 MHz, Chloroform-d) δ 6.59 (s, 2H), 4.63 (s, 1H), 4.58 (s, 1H), 3.83 (s, 6H), 3.80–3.75 (m, 1H), 3.74–3.63 (m, 1H), 3.15 (d, *J* = 12.6 Hz, 2H), 2.88–3.78 (m, 1H), 2.77–2.61 (m, 4H), 2.02 (s, 1H), 1.70 (m, 4H).

#### 3.1.4. Procedures for the Synthesis of 6,7-Dimethoxy-2-(piperidin-4-ylmethyl)-1,2,3,4-tetrahydroisoquinoline (**6**)

The (6,7-dimethoxy-3,4-dihydroisoquinolin-2(1*H*)-yl)(piperidin-4-yl)methanone (compound **5**, 9.86 mmol, 3.0 g) was reacted with LiAlH_4_ (0.053 mmol, 2.0 g) in dry THF under reflux and constant stirring for 24 h. After the completion of the reaction time, the excess of LiAlH_4_ was removed with a 5% NaOH solution and vacuum-filtered using Celite 545. Subsequently, the reaction crude was concentrated under reduced pressure, and the organic compound was extracted with five portions of 30 mL CH_2_Cl_2_. Anhydrous sodium sulfate was added to the organic phase and then filtered. The resulting organic phase was concentrated under reduced pressure, yielding a white solid that was used for the next reaction without further purification. Yield 65.2% (C_17_H_26_N_2_O_2_): ^1^H-NMR (400 MHz, Chloroform-d) δ ^1^H-NMR (400 MHz, D_2_O) δ 6.91 (s, 1H), 6.83 (s, 1H), 4.56 (d, *J* = 15.1 Hz, 1H), 4.33–4.23 (m, 1H), 3.84 (s, 8H), 3.67–3.38 (m, 3H), 3.39–3.27 (m, 2H), 3.24–3.01 (m, 4H), 2.52–2.33 (m, 1H), 2.17–2.08 (m, 2H), 1.72–1.56 (m, 2H).

#### 3.1.5. General Procedures for the Synthesis of (1-Benzylpiperidin-4-yl)(6,7-dimethoxy-3,4-dihydroisoquinolin-2(1*H*)-yl)methanone Derivatives (**7**–**11**)

The (6,7-dimethoxy-3,4-dihydroisoquinolin-2(1*H*)-yl)(piperidin-4-yl)methanone (1.27 mmol, 0.386 g) was reacted with previously synthesized benzyl chloride derivatives (1.27 mmol) and K_2_CO_3_ in a mixture of CH_3_CN/CH_2_Cl_2_ (3:1) under reflux and constant stirring for 72 h. After the reaction time, the mixture was concentrated under reduced pressure, transferred to a separatory funnel, and 20 mL of 10% K_2_CO_3_ solution was added. The organic compound was then extracted with three portions of 30 mL of CH_2_Cl_2_, and anhydrous sodium sulfate was added to dry the organic phase. The organic phase was concentrated under reduced pressure, yielding a pale-yellow oil, which was further purified by column chromatography using ethyl acetate as the mobile phase, resulting in a colorless oil. Finally, a hydrochloride salt was obtained using a 7.4% *w*/*v* HCl solution in isopropanol.

##### (1-Benzylpiperidin-4-yl)(6,7-dimethoxy-3,4-dihydroisoquinolin-2(1*H*)-yl)methanone (**7**)

Obtained as a white solid, yield 76.2% (C_24_H_30_N_2_O_3_): ^1^H-NMR (400 MHz, DMSO) δ 7.46 (s, 5H), 6.73 (s, 2H), 4.50 (s, 2H), 3.71 (s, 8H), 3.75–3.59 (m, 2H), 3.02–2.87 (m, 4H), 2.78–2.71 (m, 2H), 2.68–2.61 (m, 1H), 2.05–1.93 (m, 2H), 1.87–1.77 (m, 2H). ^13^C-NMR (101 MHz, DMSO) δ 171.62, 147.52, 147.26, 131.66, 129.48, 128.74 (2C), 125.90, 125.12, 124.93, 111.84, 109.99, 58.99, 55.53 (2C), 50.45, 46.02, 43.53, 42.52, 35.26, 28.65, 27.32, 25.62 (2C). IR (cm^−^^1^): 3008, 2948, 2833, 1614, 1221, 1116. HRMS *m*/*z* calcd. for C_24_H_30_N_2_O_3_ (M + H), 395.2329; found, 395.2324.

##### (6,7-Dimethoxy-3,4-dihydroisoquinolin-2(1*H*)-yl)(1-(3-fluorobenzyl)piperidin-4-yl)methanone (**8**)

Obtained as a white solid, yield 81.4% (C_24_H_29_FN_2_O_3_): ^1^H-NMR (400 MHz, DMSO) δ 7.65–7.58 (m, 1H), 7.56–7.44 (m, 2H), 7.36–7.25 (m, 1H), 6.73 (s, 2H), 4.29 (s, 2H), 3.72 (s, 8H), 3.67–3.59 (m, 2H), 3.07–2.88 (m, 4H), 2.80–2.73 (m, 2H), 2.70–2.61 (m, 1H), 2.13–1.94 (m, 2H), 1.89–1.77 (m, 2H).^13^C-NMR (101 MHz, DMSO) δ 171.61, 161.91 (d, *J* = 244.3 Hz), 147.41, 147.34, 130.77, 130.69, 127.86, 125.89, 125.11, 118.45 (d, *J* = 22.2 Hz), 116.40 (d, *J* = 20.8 Hz), 111.83, 109.98, 58.11, 55.55, 55.52, 50.49, 46.03, 43.52, 42.51, 35.30, 28.65, 27.32, 25.58. ^13^C-NMR (101 MHz, DMSO) δ, IR (cm^−^^1^): 3037, 2942, 2819, 1620, 1229, 1116. HRMS *m*/*z* calcd. for C_24_H_29_FN_2_O_3_ (M + H), 413.2235; found, 413.2233.

##### (1-(3-Chlorobenzyl)piperidin-4-yl)(6,7-dimethoxy-3,4-dihydroisoquinolin-2(1*H*)-yl)methanone (**9**)

Obtained as a white solid, yield 90.6% (C_24_H_29_ClN_2_O_3_): ^1^H-NMR (400 MHz, DMSO) δ 7.79 (s, 1H), 7.65–7.58 (m, 1H), 7.56–7.44 (m, 2H), 6.73 (s, 2H), 4.28 (s, 2H), 3.71 (s, 9H), 3.67–3.59 (m, 1H), 3.01–2.87 (m, 4H), 2.79–2.72 (m, 2H), 2.68–2.61 (m, 1H), 2.12–1.92 (m, 2H), 1.90–1.76 (m, 2H). ^13^C-NMR (101 MHz, DMSO) δ 172.08, 147.87, 147.80, 133.71, 131.94, 131.01 (2C), 130.90, 129.92, 126.35, 125.57, 112.30, 110.45, 58.48, 56.02, 55.99, 50.95, 46.49, 43.99, 42.98, 35.72, 29.12, 27.78, 26.05. IR (cm^−^^1^): 3064, 2942, 2815, 1618, 1227, 1112. HRMS *m*/*z* calcd. for C_24_H_29_ClN_2_O_3_ (M + H), 429.1939; found, 429.1935.

##### (1-(3-Bromobenzyl)piperidin-4-yl)(6,7-dimethoxy-3,4-dihydroisoquinolin-2(1*H*)-yl)methanone (**10**)

Obtained as a white solid, yield 60.0% (C_24_H_29_BrN_2_O_3_): ^1^H-NMR (400 MHz, DMSO-d6) δ 7.90 (s, 1H), 7.66 (t, *J* = 8.6 Hz, 2H), 7.43 (t, *J* = 7.8 Hz, 1H), 6.73 (s, 2H), 4.56–4.51 (s, 2H), 3.70 (s, 8H), 3.69–3.60 (m, 2H), 3.02–2.85 (m, 4H), 2.82–2.72 (m, 2H), 2.71–2.57 (m, 1H), 2.19–1.89 (m, 2H), 1.89–1.71 (m, 2H). ^13^C-NMR (101 MHz, DMSO) δ 172.08, 147.87, 147.80, 134.77, 132.81, 132.62, 131.25 (2C), 126.35, 125.57, 122.30, 112.29, 110.44, 58.43, 56.00 (2C), 50.95, 46.49, 43.99, 42.98, 35.71, 29.12, 27.78, 26.05. IR (cm^−^^1^): 3055, 2932, 2813, 1620, 1264, 1110. HRMS *m*/*z* calcd. for C_24_H_29_^81^BrN_2_O_3_ (M + H), 475.1417; found, 475.1414 and cald for. C_24_H_29_^79^BrN_2_O_3_ (M + H) 473.1427; found, 473.1424.

##### (1-(2,4-Dichlorobenzyl)piperidin-4-yl)(6,7-dimethoxy-3,4-dihydroisoquinolin-2(1*H*)-yl)methanone (**11**)

Obtained as a white solid, yield 75.0% (C_24_H_28_Cl_2_N_2_O_3_): ^1^H-NMR (400 MHz, DMSO) δ 7.99–7.94 (m, 1H), 7.80–7.72 (m, 1H), 7.65–7.58 (m, 1H), 6.74 (s, 2H), 4.29 (s, 2H), 3.71 (s, 8H), 3.69–3.67 (m, 1H), 3.67–3.61 (m, 1H), 2.96–2.92 (m, 4H), 2.79–2.72 (m, 2H), 2.69–2.62 (m, 1H), 1.98–1.94 (m, 2H), 1.87–1.81 (m, 2H). ^13^C-NMR (101 MHz, DMSO) δ 172.08, 147.87, 147.80, 134.18, 132.87, 132.54, 131.72, 131.27, 130.98, 126.34, 125.56, 112.29, 110.44, 57.72, 56.01, 55.99, 50.87, 46.50, 43.99, 42.98, 35.68, 29.12, 27.79, 26.06. IR (cm^−^^1^): 3072, 2938, 2813, 1620, 1244, 1112. HRMS *m*/*z* calcd. for C_24_H_28_Cl_2_N_2_O_3_ (M + H), 463.1550; found, 463.1541.

#### 3.1.6. General Procedures for the Synthesis of (4-((6,7-Dimethoxy-3,4-dihydroisoquinolin-2(1*H*)-yl)methyl)piperidin-1-yl)(phenyl)methanone (**12**–**16**)

The 6,7-dimethoxy-2-(piperidin-4-ylmethyl)-1,2,3,4-tetrahydroisoquinoline (1.27 mmol, 0.386 g) was reacted with benzoyl chloride derivatives (1.27 mmol) in CH_2_Cl_2_ at room temperature under constant stirring for 24 h. After the completion of the reaction time, the reaction crude was concentrated under reduced pressure, transferred to a separatory funnel, and a 10% K_2_CO_3_ solution was added. Subsequently, the organic compound was extracted with three portions of 30 mL of CH_2_Cl_2_, and anhydrous sodium sulfate was added to the organic phase. The organic phase was filtered and concentrated under reduced pressure, resulting in a pale-yellow oil, which was purified by column chromatography using ethyl acetate as the mobile phase, yielding a colorless oil. Finally, the hydrochloride salts of the compounds were obtained using a 7.4% *w*/*v* HCl solution in isopropanol.

##### (4-((6,7-Dimethoxy-3,4-dihydroisoquinolin-2(1*H*)-yl)methyl)piperidin-1-yl)(phenyl)methanone (**12**)

Obtained as a white solid, yield 80.6% (C_24_H_30_N_2_O_3_): ^1^H-NMR (400 MHz, D_2_O) δ 7.59–7.49 (m, 3H), 7.43 (d, *J* = 1.8 Hz, 2H), 6.92 (s, 1H), 6.83 (s, 1H), 4.63–4.46 (m, 2H), 4.27 (d, *J* = 15.2 Hz, 1H), 3.90–3.73 (m, 8H), 3.50–3.34 (m, 1H), 3.34–3.15 (m, 4H), 3.13–2.95 (m, 2H), 2.48–2.31 (m, 1H), 2.05–1.93 (m, 1H), 1.86–1.71 (m, 1H), 1.55–1.27 (m, 2H). ^13^C- (101 MHz, D_2_O) δ 172.34, 148.15, 147.29, 134.58, 130.35, 128.82 (2C), 126.42 (2C), 123.47, 119.21, 111.53, 109.63, 60.03, 59.37, 55.79, 55.73, 53.06, 47.42, 41.92, 30.76, 29.54, 28.81, 23.91. IR (cm^−^^1^): 3064, 2953, 2833, 1631, 1223, 1118. HRMS *m*/*z* calcd. for C_24_H_30_N_2_O_3_ (M + H), 395.2329; found, 395.2313.

##### (4-((6,7-Dimethoxy-3,4-dihydroisoquinolin-2(1*H*)-yl)methyl)piperidin-1-yl)(3-fluorophenyl)methanone (**13**)

Obtained as a white solid, yield 61.3% (C_24_H_29_FN_2_O_3_): ^1^H-NMR (400 MHz, D_2_O) δ 7.60–7.43 (m, 1H), 7.35–7.15 (m, 3H), 6.93 (s, 1H), 6.84 (s, 1H), 4.64–4.42 (m, 2H), 3.85 (s, 6H), 3.82–3.74 (m, 2H), 3.69–3.59 (m, 1H), 3.53–3.40 (m, 1H), 3.30–3.21 (m, 3H), 3.21–3.08 (m, 2H), 3.07–2.95 (m, 1H), 2.49–2.31 (m, 1H), 2.07–1.95 (m, 1H), 1.87–1.76 (m, 1H), 1.52–1.26 (m, 2H). ^13^C-NMR (101 MHz, D_2_O) δ 170.80, 162.33 (d, *J* = 245.8 Hz), 148.15, 147.28, 136.53, 130.93, 123.46, 122.36, 119.20, 117.17 (d, *J* = 21.2 Hz), 113.53 (d, *J* = 23.5 Hz), 111.53, 109.62, 59.99, 59.39, 55.78, 55.72, 53.54, 47.35, 41.93, 30.73, 29.49, 28.75, 23.92. IR (cm^−^^1^): 3060, 2950, 2839, 1631, 1233, 1124. HRMS *m*/*z* calcd. for C_24_H_29_FN_2_O_3_ (M + H), 413.2235; found, 413.2227.

##### (3-Chlorophenyl)(4-((6,7-dimethoxy-3,4-dihydroisoquinolin-2(1*H*)-yl)methyl)piperidin-1-yl)methanone (**14**)

Obtained as a white solid, yield 64.2% (C_24_H_29_ClN_2_O_3_): ^1^H-NMR (400 MHz, DMSO) δ 7.57–7.42 (m, 3H), 7.37–7.30 (m, 1H), 6.80 (s, 2H), 4.58–4.34 (m, 2H), 4.24–4.08 (m, 1H), 3.73 (d, *J* = 5.1 Hz, 6H), 3.65–3.45 (m, 2H), 3.35–3.21 (m, 2H), 3.19–3.03 (m, 3H), 2.95–2.83 (m, 2H), 2.35–2.21 (m, 1H), 2.14–1.79 (m, 2H), 1.33–1.16 (m, 2H). ^13^C-NMR 13C NMR (101 MHz, DMSO) δ 167.76, 148.77, 148.08, 138.81, 133.67, 130.98, 129.79, 126.89, 125.69, 123.77, 120.22, 111.91, 110.21, 60.07, 56.04 (2C), 56.01, 52.19, 49.84, 47.07, 41.53, 31.22, 30.02, 24.49. IR (cm^−^^1^): 3072, 2924, 2856, 1629, 1229, 1130. HRMS *m*/*z* calcd. for C_24_H_29_ClN_2_O_3_ (M + H), 429.1939; found, 429.1950.

##### (3-Bromophenyl)(4-((6,7-dimethoxy-3,4-dihydroisoquinolin-2(1*H*)-yl)methyl)piperidin-1-yl)methanone (**15**)

Obtained as a white solid, yield 74.8% (C_24_H_29_BrN_2_O_3_): ^1^H-NMR (400 MHz, DMSO) δ 7.76–7.63 (m, 1H), 7.57 (s, 1H), 7.48–7.33 (m, 2H), 6.80 (d, *J* = 4.7 Hz, 2H), 4.51–4.38 (m, 2H), 4.23–4.10 (m, 1H), 3.73 (d, *J* = 5.1 Hz, 6H), 3.68–3.45 (m, 2H), 3.31–3.18 (m, 2H), 3.15–3.02 (m, 3H), 2.95–2.79 (m, 2H), 2.33–2.17 (m, 1H), 2.10–1.71 (m, 2H), 1.34–1.19 (m, 2H). ^13^C-NMR (101 MHz, DMSO) δ 167.63, 148.76, 148.08, 139.05, 132.68, 131.21, 129.73, 126.05, 123.80, 122.17, 120.27, 111.93, 110.23, 60.06, 56.05 (2C), 56.01, 52.18, 49.81, 47.10, 41.56, 31.26, 30.64, 24.50. IR (cm^−^^1^): 3072, 2930, 2860, 1625, 1227, 1124. HRMS *m*/*z* calcd. for C_24_H_29_^81^BrN_2_O_3_ (M + H), 475.1417; found, 475.1436 and cald for C_24_H_29_^79^BrN_2_O_3_ (M + H) 473,1427; found, 473.1445.

##### (4-((6,7-Dimethoxy-3,4-dihydroisoquinolin-2(1*H*)-yl)methyl)piperidin-1-yl)(3-methoxyphenyl)methanone (**16**)

Obtained as a white solid, yield 61.6% (C_25_H_32_N_2_O_4_): ^1^H-NMR (400 MHz, D_2_O) δ 7.46 (t, *J* = 7.9 Hz, 1H), 7.17–7.09 (m, 1H), 7.06–6.98 (m, 2H), 6.92 (s, 1H), 6.83 (s, 1H), 4.57 (d, *J* = 13.5 Hz, 1H), 4.40 (s, 2H), 3.91–3.82 (m, 9H), 3.82–3.72 (m, 1H), 3.71–3.50 (m, 2H), 3.34–3.20 (m, 3H), 3.18–3.11 (m, 2H), 3.07–2.95 (m, 1H), 2.44–2.30 (m, 1H), 2.00 (d, *J* = 13.2 Hz, 1H), 1.79 (d, *J* = 13.2 Hz, 1H), 1.51–1.25 (m, 2H). ^13^C-NMR (101 MHz, D_2_O) δ 171.76, 159.01, 148.15, 147.29, 136.06, 130.32, 123.46, 119.21, 118.95, 115.84, 111.96, 111.53, 109.63, 59.99, 55.79, 55.73, 55.50, 52.98, 50.12, 47.37, 41.88, 30.77, 29.56, 28.81, 23.92. IR (cm^−^^1^): 3018, 2936, 2854, 1625, 1229, 1124. HRMS *m*/*z* calcd. for C_25_H_32_N_2_O_4_ (M + H), 425.2435; found, 425.2468.

#### 3.1.7. General Procedure for the Synthesis of 2-Phenylacetate of (1-Benzylpiperidin-4-yl)methyl Derivatives (**18**–**20**), 2,2-Diphenylacetate of (1-Benzylpiperidin-4-yl)methyl (**21**) and 2-Naphthoate of (1-Benzylpiperidin-4-yl)methyl (**22**)

In a 100 mL round-bottom flask, the derivative of 2-phenylacetyl chloride (2.79 mmol–3.09 mmol), 2,2-diphenylacetyl chloride (2.50 mmol), or 2-naphthoyl chloride (2.78 mmol) was reacted with (1-benzylpiperidin-4-yl)methanol (compound **17**, 2.50 mmol–3.09 mmol) in 50 mL of CH_2_Cl_2_ at room temperature with constant stirring for 24 h (Figure 3). After the reaction time was completed, a 10% NaHCO_3_ solution was added to the reaction mixture, and it was transferred to a separatory funnel. The compound was extracted using three portions of 30 mL of CH_2_Cl_2_, dried with anhydrous sodium sulfate, and then filtered. Finally, it was concentrated under reduced pressure in a rotary evaporator, forming a yellow oil. The hydrochloride salts of the compounds were obtained using a 7.4% HCl solution in isopropanol.

##### (1-Benzylpiperidin-4-yl)methyl-2-phenylacetate (**18**)

Obtained as a white solid, yield 95.3% (C_21_H_25_NO_2_): ^1^H-NMR (400 MHz, CDCl_3_) δ 7.46–7.15 (m, 10H), 3.96 (d, *J* = 6.1 Hz, 2H), 3.62 (s, 2H), 3.54 (s, 2H), 2.97–2.87 (m, 2H), 2.00 (td, *J* = 11.9, 2.4 Hz, 2H), 1.72–1.61 (m, 3H), 1.50–1.26 (m, 2H). ^13^C-NMR (101 MHz, CDCl_3_) δ 171.60, 136.75, 134.12, 129.64, 129.41, 129.28, 128.60, 128.34, 127.46, 127.13, 126.30, 68.98, 62.70, 52.65 (2C), 41.45, 34.98, 28.22 (2C). IR (cm^−^^1^): 3025, 2941, 1726, 1255, 1147. HRMS *m*/*z* calcd. for C_21_H_25_NO_2_ (M + H), 324.1958; found, 324.1964.

##### (1-Benzylpiperidin-4-yl)methyl-2-(4-fluorophenyl)acetate (**19**)

Obtained as a white solid, yield 62.1% (C_21_H_24_FNO_2_): ^1^H-NMR (400 MHz, CDCl_3_) δ 7.45–7.32 (m, 7H), 7.31–7.24 (m, 2H), 4.02 (d, *J* = 6.0 Hz, 2H), 3.71 (s, 2H), 3.64 (s, 2H), 3.07 (d, *J* = 3.4 Hz, 2H), 2.19 (t, *J* = 2.5 Hz, 2H), 1.79–1.65 (m, 3H), 1.60–1.45 (m, 2H). ^13^C-NMR (101 MHz, D_2_O) δ 174.56, 163.04, 160.63, 131.20, 131.12, 130.17, 129.82, 129.24, 128.61, 115.53, 115.31, 68.21, 60.67, 51.85 (2C), 39.68, 32.50, 25.61 (2C). IR (cm^−^^1^): 3047, 2941, 1725, 1218, 1146. HRMS *m*/*z* calcd. for C_21_H_24_FNO_2_ (M + H), 342.1864; found, 342.1884.

##### (1-Benzylpiperidin-4-yl)methyl-2-(4-chlorophenyl)acetate (**20**)

Obtained as a white solid, yield 38.9% (C_21_H_24_ClNO_2_): ^1^H-NMR (400 MHz, CDCl_3_) δ 7.30–7.23 (m, 2H), 7.23–7.18 (m, 1H), 7.18–7.12 (m, 1H), 6.97–6.86 (m, 5H), 3.85 (d, *J* = 6.0 Hz, 2H), 3.69 (s, 2H), 3.48 (s, 2H), 3.10–3.01 (m, 2H), 2.22–2.12 (m, 2H), 1.67–1.55 (m, 3H), 1.54–1.40 (m, 2H). ^13^C-NMR (101 MHz, CDCl_3_) δ 171.23, 137.84, 133.13, 132.60, 130.71, 129.63, 129.41, 128.77 (2C), 128.35, 128.31, 127.23, 69.37, 63.19, 53.03 (2C), 40.75, 35.24, 28.69 (2C). IR (cm^−^^1^): 3029, 2941, 1735, 1249, 1146. HRMS *m*/*z* calcd. for C_21_H_24_ClNO_2_ (M + H), 358,1568; found, 395.2324.

##### (1-Benzylpiperidin-4-yl)methyl-2,2-diphenylacetate (**21**)

Obtained as a white solid, yield 36.7% (C_27_H_29_NO_2_): ^1^H NMR (400 MHz, CDCl_3_) δ 7.46–7.16 (m, 15H), 5.03 (s, 1H), 4.01 (d, *J* = 6.6 Hz, 2H), 3.75 (s, 2H), 3.14–3.03 (m, 2H), 2.25–2.13 (m, 2H), 1.78–1.60 (m, 3H), 1.59–1.43 (m, 2H). ^13^C-NMR (101 MHz, D_2_O) δ 172.21, 131.21 (4C), 130.76, 130.14 (4C), 129.38, 129.21 (4C), 129.01, 128.69, 128.63 (2C), 65.20, 60.68, 52.14 (2C), 48.68, 35.26, 25.76 (2C). IR (cm^−^^1^): IR (cm^−^^1^): 3025, 2886, 1720, 1226, 1043. HRMS *m*/*z* calcd. for C_27_H_29_NO_2_ (M + H), 400.2271; found, 400.2290.

##### (1-Benzylpiperidin-4-yl)methyl-2-naphthoate (**22**)

Obtained as a white solid, yield 50.0% (C_24_H_25_NO_2_): ^1^H-NMR (400 MHz, CDCl_3_) δ 8.62 (s, 1H), 8.05–7.96 (m, 2H), 7.94–7.82 (m, 2H), 7.62–7.46 (m, 4H), 7.45–7.33 (m, 3H), 4.28 (d, *J* = 5.4 Hz, 2H), 4.03 (s, 2H), 3.36 (d, *J* = 11.9 Hz, 2H), 2.62–2.52 (m, 2H), 2.20–1.87 (m, 5H). ^13^C-NMR (101 MHz, CDCl_3_) δ 166.59, 135.68, 132.59, 131.60, 131.38, 130.19, 130.09, 129.62, 129.36 (2C), 129.29, 128.51, 128.35, 127.78, 126.95, 126.83, 125.15, 67.63, 65.75, 60.94, 52.35, 51.94, 29.75 (2C), 25.91. IR (cm^−^^1^): IR (cm^−^^1^): 3047, 2936, 1707, 1230, 1040. HRMS *m*/*z* calcd. for C_24_H_25_NO_2_ (M + H), 360.1958; found, 360.1953.

### 3.2. Acetylcholinesterase (AChE) and Butyrylcholinesterase (BuChE) Inhibitory Activity

The inhibition of AChE and BuChE activities was determined using the spectrophotometric method according to Ellman [25] and modified from Mella et al. [26]. Briefly, 50 µL of AChE or BuChE (0.50 U/mL) in phosphate-buffered saline (8 mM NaH_2_PO_4_, 2.3 mM Na_2_HPO_4_, 0.15 M NaCl, pH 7.5) and 50 μL of the samples (compounds at different concentrations) dissolved in the same buffer were added to the wells. The plates were incubated for 30 min at 25 °C before the addition of 100 μL of the substrate solution (0.04 M Na_2_HPO_4_, 0.2 mM 5,5′-dithiobis (2-nitrobenzoic acid) (DTNB), acetylthiocholine iodide (ATCI), or butyrylthiocholine iodide (BTCI), 0.24 mM) in HPLC-grade water. After 3 min (AChE) or 5 min (BuChE), the absorbance was read at 405 nm in a microplate reader (BIOBASE-EL10A). Enzymatic inhibition was calculated as a percentage compared to a control using a buffer with no inhibitor. The IC_50_ values are reported as the mean ± SD of three determinations. Galanthamine hydrobromide (Sigma, St. Louis, MO, USA) was used as a positive control. The data were analyzed using Prism 10.3.0 software (GraphPad Software, Inc., Boston, MA, USA).

### 3.3. Protocol Binding of [^3^H]-Paroxetine on h-SERT Cells

The cellular background HEK293 containing 400 µL of h-SERT (Code RBHSTM400UA, Perkin-Elmer, Santiago, Chile) was diluted in 12 Eppendorf tubes, containing a storage buffer solution of Tris-HCl 50 mM (pH 7.4), EDTA 0.5 mM, MgCl_2_ 10 mM, and 10% sucrose, obtaining a final volume between 260 and 340 µL, which was finally stored at −80 °C [27]. Each Eppendorf tube was incubated with 50 mM Tris HCl buffer (pH 7.4), 120 mM NaCl, 5 mM KCl, and the drugs under study using increasing concentrations, in the presence of 2 nM of [^3^H]-paroxetine (specific activity 23.1 C_i_/mmol, Code NET86925UC, Perkin-Elmer, Shelton, CT, USA) with a final volume of 250 µL [27]. Non-specific binding was determined using 25 mM fluoxetine. After 30 min at 27 °C, the incubation was stopped by rapid filtration on a Whatman GF/C filter preabsorbed in 0.5% polyethylenimine (PEI), washed with cold working buffer solution, 3 × 3 mL, filtered, and scintillation liquid was added. The radioactivity was measured by liquid scintillation spectrometry (MicroBeta 2450 microplate counter, Perkin-Elmer, Shelton, CT, USA). The data were plotted by non-linear regression variable inhibitor-response dose (Prism 10.3.0, GraphPad, Boston, MA, USA) to estimate the IC_50_ and K_i_ values for the tested compounds using the Cheng–Prusoff equation.

### 3.4. Molecular Docking Study

The molecular docking of the compounds on models of AChE and the central binding site of h-SERT was performed using the Lamarckian genetic algorithm search method with AutoDock v4.0 software (San Diego, CA, USA). The X-ray crystal structures of human AChE and SERT were obtained from the Protein Data Bank (PDB: 1B41 and 6AWN, respectively). Compounds (**1**–**15**) were built using the 2D Sketcher program from Schrödinger Suites 2018 (Maestro, version 11.8., Schrödinger, LLC, New York, NY, USA.) [28,29]. Once the protein and compound models were prepared, molecular docking simulations were performed using the SwissDock server, which utilizes Autodock Vina for the docking process [28,29]. A grid with dimensions of 30 × 30 × 30 Å was generated for the docking simulation. However, if the computation time exceeded 10 min due to server limitations, the grid size was reduced accordingly. The docking results were analyzed using Maestro software from Schrödinger Suites 2018 (Maestro, version 11.8., Schrödinger, LLC, New York, NY, USA) to evaluate the interactions between the compounds and the amino acid residues of the protein. To validate the results, donepezil was used as a reference compound. Donepezil was evaluated using the same methodology applied to compounds (**1**–**15**).

## 4. Conclusions

The chemical structures of compounds **7**–**22** were confirmed using ^1^H NMR, ^13^C NMR, FT-IR, and HRMS. Radioligand binding and inhibition assays, supported by docking studies, were performed on this series of novel compounds targeting AChE, BuChE, and h-SERT. Unfortunately, our results indicate that obtaining a compound with balanced activity on the three targets was not possible. While some compounds exhibited good anticholinesterase activity, their affinity for SERT was low, and vice versa. Compound **19** was the most potent AChE/ BuChE inhibitor, while compounds **12**–**16** showed the highest affinity for SERT. Remarkably, compound **21** exhibited an interesting and novel polypharmacological profile, displaying a good and very selective inhibitory activity against BuChE while showing an affinity in the same range for SERT. Globally, our results highlight the challenge of designing promiscuous ligands for these targets. They also suggest that future structural modifications should enhance simultaneous interactions with the active sites of different combinations of these targets, aimed at optimizing their therapeutic potential in the treatment of Alzheimer’s disease.

## Figures and Tables

**Table 1:** IC_50_ or K_i_ values (µM) for synthesized compounds (**7**–**16** and **18**–**22**) against AChE, BuChE, and h-SERT.

Compound	AChE IC_50_ (µM)	BuChE IC_50_ (µM)	h-SERT K_I_ (µM)
**7**	34.37 ± 0.51	-	-
**8**	41.16 ± 3.17	-	-
**9**	34.47 ± 1.97	268.20 ± 13.83	-
**10**	28.28 ± 0.87	426.30 ± 28.61	-
**11**	-	-	-
**12**	289.60 ± 10.94	-	2.62 ± 0.82
**13**	-	-	1.91 ± 0.77
**14**	-	-	2.43 ± 0.24
**15**	242.40 ± 24.37	-	1.47 ± 0.73
**16**	439.10 ± 28.02	-	1.59 ± 0.58
**18**	199.90 ± 8.61	256.20 ± 5.03	27.1 ± 3.40
**19**	5.10 ± 0.24	26.78 ± 0.81	196.6 ± 11.34
**20**	259.10 ± 14.57	-	-
**21**	-	6.16 ± 0.29	25.5 ± 1.01
**22**	61.70 ± 2.56	59.30 ± 1.42	26.9 ± 2.57
GAL	1.19 ± 0.046	29.05 ± 0.972	-

The non-specific binding at h-SERT exhibited radioligand displacement by [^3^H]-paroxetine at a 2 nM concentration; the dissociation constant (K_d_) used to estimate K_i_ was 1.31 nM for [^3^H]-paroxetine. Galantamine hydrobromide (GAL) was used as a positive control in AChE and BuChE inhibition assays. The IC_50_ values are expressed as means ± SD of three determinations. The symbol (-) indicates no effect with IC_50_ > 500 µM in AChE and K_i_ > 100 µM in h-SERT.

## Data Availability

The original contributions presented in this study are included in the article/Supplementary Material. Further inquiries can be directed to the corresponding author.

## Supplementary Materials

The following supporting information can be downloaded at: https://www.mdpi.com/article/10.3390/molecules30143047/s1, Figures S1–S33 correspond to 1-D NMR spectra and 2-D spectra HSQC and HMQC Figures S49–S52. The HRMS figures correspond to Figures S34–S48 and concentration-response curves correspond to Figures S53 and S54.

## References

[1] Mount C., Downton C. (2006). Alzheimer disease: Progress or profit?. Nat Med..

[2] Cummings J.L., Doody R., Clark C. (2007). Disease-modifying therapies for Alzheimer disease: Challenges to early intervention. Neurology.

[3] Yiannopoulou K.G., Papageorgiou S.G. (2013). Current and future treatments for Alzheimer’s disease. Ther. Adv. Neurol. Disord..

[4] Chen G., Xu T., Yan Y., Zhou Y., Jiang Y., Melcher K., Xu H.E. (2017). Amyloid beta: Structure, biology and structure-based therapeutic development. Acta Pharmacol. Sin..

[5] Galimberti D., Scarpini E. (2011). Disease-modifying treatments for Alzheimer’s disease. Ther. Adv. Neurol. Disord..

[6] Klafki H.-W., Staufenbiel M., Kornhuber J., Wiltfang J. (2006). Therapeutic approaches to Alzheimer’s disease. Brain..

[7] Francis P.T., Palmer A.M., Snape M., Wilcock G.K. (1999). The cholinergic hypothesis of Alzheimer’s disease: A review of progress. J. Neurol. Neurosurg. Psychiatry.

[8] Foster D., Choi D., Conn P.J., Rook J. (2014). Activation of M1 and M4 muscarinic receptors as potential treatments for Alzheimer’s disease and schizophrenia. Neuropsychiatr. Dis. Treat..

[9] Onajole O.K., Vallerini G.P., Eaton J.B., Lukas R.J., Brunner D., Caldarone B.J., Kozikowski A.P. (2016). Synthesis and Behavioral Studies of Chiral Cyclopropanes as Selective α4β2-Nicotinic Acetylcholine Receptor Partial Agonists Exhibiting an Antidepressant Profile. Part III. ACS Chem. Neurosci..

[10] Strachan J.-P., Kombo D.C., Mazurov A., Heemstra R., Bhatti B.S., Akireddy R., Murthy S., Miao L., Jett J.E., Speake J. (2014). Identification and pharmacological characterization of 3,6-diazabicyclo[3.1.1]heptane-3-carboxamides as novel ligands for the α4β2 and α6/α3β2β3 nicotinic acetylcholine receptors (nAChRs). Eur. J. Med. Chem..

[11] Barret K.E., Barman S.M., Boitano S., Brooks H.L., Barrett K.E., Boitano S., Barman S.M., Brooks H.L., Michael Weitz B.P.K. (2012). Ganong’ s Review of Medical Physiology.

[12] Sharma K. (2019). Cholinesterase inhibitors as Alzheimer’s therapeutics (Review). Mol. Med. Rep..

[13] Silman I., Sussman J.L. (2005). Acetylcholinesterase: “classical” and “non-classical” functions and pharmacology. Curr. Opin. Pharmacol..

[14] Pachón-Angona I., Refouvelet B., Andrýs R., Martin H., Luzet V., Iriepa I., Moraleda I., Diez-Iriepa D., Oset-Gasque M.-J., Marco-Contelles J. (2019). Donepezil + chromone + melatonin hybrids as promising agents for Alzheimer’s disease therapy. J. Enzym. Inhib. Med. Chem..

[15] Marucci G., Buccioni M., Ben D.D., Lambertucci C., Volpini R., Amenta F. (2021). Efficacy of acetylcholinesterase inhibitors in Alzheimer’s disease. Neuropharmacology..

[16] Lilienfeld S. (2002). Galantamine — a Novel Cholinergic Drug with a Unique Dual Mode of Action for the Treatment of Patients with Alzheimer’s Disease. CNS Drug Rev..

[17] Fernández M., Gobartt A.L., Balañá M. (2010). Behavioural symptoms in patients with Alzheimer’s disease and their association with cognitive impairment. BMC Neurol..

[18] Orgeta V., Tabet N., Nilforooshan R., Howard R. (2017). Efficacy of Antidepressants for Depression in Alzheimer’s Disease: Systematic Review and Meta-Analysis. Leoutsakos J-M, editor. J. Alzheimer’s Dis..

[19] Singh B., Day C.M., Abdella S., Garg S. (2024). Alzheimer’s disease current therapies, novel drug delivery systems and future directions for better disease management. J. Control. Release.

[20] Méndez-Rojas C., Quiroz G., Faúndez M., Gallardo-Garrido C., Pessoa-Mahana C.D., Chung H., Gallardo-Toledo E., Saitz-Barría C., Araya-Maturana R., Kogan M.J. (2018). Synthesis and biological evaluation of potential acetylcholinesterase inhibitors based on a benzoxazine core. Arch. Pharm..

[21] Giancola J.B., Bonifazi A., Cao J., Ku T., Haraczy A.J., Lam J., Rais R., Coggiano M.A., Tanda G., Newman A.H. (2020). Structure-activity relationships for a series of (Bis(4-fluorophenyl)methyl)sulfinylethyl-aminopiperidines and -piperidine amines at the dopamine transporter: Bioisosteric replacement of the piperazine improves metabolic stability. Eur. J. Med. Chem..

[22] Añazco T., Werner T., Torres M.J., Hornos-Carneiro M.F., Fernández J., Zivkovic A., Salas C.O., Castro-Álvarez A., Gutiérrez M., Stark H. (2025). First in class pyrrolo[2,3-*d*]pyrimidine derivatives fused to fluorobenzylpiperidines as dual ligands of acetylcholinesterase and histamine H_3_ receptor. Arch. Pharm..

[23] Zhou S., Huang G. (2022). The biological activities of butyrylcholinesterase inhibitors. Biomed. Pharmacother..

[24] Coleman J.A., Navratna V., Antermite D., Yang D., Bull J.A., Gouaux E. (2020). Chemical and structural investigation of the paroxetine-human serotonin transporter complex. eLife..

[25] Ellman G.L., Courtney K.D., Andres V., Featherstone R.M. (1961). A new and rapid colorimetric determination of acetylcholinesterase activity. Biochem. Pharmacol..

[26] Mella M., Moraga-Nicolás F., Machuca J., Quiroz A., Mutis A., Becerra J., Astudillo Á., Hormazábal E. (2022). Acetylcholinesterase inhibitory activity from *Amaryllis belladonna* growing in Chile: Enzymatic and molecular docking studies. Nat. Prod. Res..

[27] Smith P.K., Krohn R.I., Hermanson G.T., Mallia A.K., Gartner F.H., Provenzano M.D., Fujimoto E.K., Goeke N.M., Olson B.J., Klenk D.C. (1985). Measurement of protein using bicinchoninic acid. Anal. Biochem..

[28] Bugnon M., Röhrig U.F., Goullieux M., Perez M.A.S., Daina A., Michielin O., Zoete V. (2024). SwissDock 2024: Major enhancements for small-molecule docking with Attracting Cavities and AutoDock Vina. Nucleic Acids Res..

[29] Eberhardt J., Santos-Martins D., Tillack A.F., Forli S. (2021). AutoDock Vina 1.2.0: New Docking Methods, Expanded Force Field, and Python Bindings. J. Chem. Inf. Model..

