# Supplementary material for: Design, Synthesis, and Evaluation of 1-Benzylpiperidine and 1-Benzoylpiperidine Derivatives as Dual-Target Inhibitors of Acetylcholinesterase and Serotonin Transporter for Alzheimer′s Disease
Source: Molecules. 2025 Jul 21;30(14):3047. doi: 10.3390/molecules30143047 (PMC12299174; doi:10.3390/molecules30143047)
Supplement: Supplementary file 1 [file molecules-30-03047-s001.zip › molecules-3693675-supplementary.pdf]

Supplementary material.

Design, Synthesis, and Evaluation of 1-Benzylpiperidine and 1-benzoylpiperidine Derivatives as Dual-Target Inhibitors of Acetylcholinesterase and Serotonin Transporter for Alzheimer's Disease.

Juan Pablo González-Gutiérrez[1*]; Damián Castillo-Ríos[1]; Víctor Ríos C.[2]; Ignacio Alejandro González-Gutiérrez[3]; Dánae Flores Melivilu[4]; Emilio Hormazábal Uribe[5,6]; Felipe Moraga-Nicolás[5,6]; Kerim Segura[7]; Valentina Hernandez[7]; Amaury Farías-Cea[8,9]; Hernán Armando Pessoa-Mahana[10]; Miguel Iván Reyes-Parada[1,11] and Patricio Iturriaga-Vásquez[7*].

1 Instituto de Ciencias Aplicadas, Facultad de Ingeniería, Universidad Autónoma de Chile, Talca 3467987, Chile; juan.gonzalez01@uautonoma.cl (J.P.G.-G.)

2 Electrical Signaling in Plants (ESP) Laboratory – Center of Bioinformatics, Simulation and Modeling (CBSM), Faculty of Engineering, Universidad de Talca, Campus Talca, Avenida Lircay, Talca CL-3460000, Chile.

3 Interno de Medicina, Facultad de Medicina y Ciencias de la salud, Universidad Mayor, Huechuraba 8580000, Chile.

4 Carrera de Bioquímica, Departamento de Ciencias Químicas y Recursos Naturales, Facultad de Ingeniería y Ciencias, Universidad de La Frontera, Temuco, Chile

5 Laboratorio de Química Ecológica, Departamento de Ciencias Químicas y Recursos Naturales, Universidad de La Frontera, Temuco, Chile; emilio.hormazabal@ufrontera.cl

6 Centro de Excelencia de Investigación Biotecnológica Aplicada al Medio Ambiente (CIBAMA), Universidad de La Frontera, Temuco, Chile; felipe.moraga@ufrontera.cl

7 Laboratorio de Farmacología Molecular y Química Medicinal, Facultad de Ingeniería y Ciencias, Universidad de La frontera, Temuco 4811230, Chile; patricio.iturriaga@ufrontera.cl (P.I-V)

8 Laboratorio de Bioquímica y Farmacología Molecular, Escuela de Ciencias, Facultad de Ciencias de la Vida, Universidad Viña del Mar, Viña del Mar 2572007, Chile.

9 Escuela de Educación, Facultad de Ciencias Jurídicas, Sociales y de la Educación, Universidad Viña del Mar, Viña del Mar 2580022, Chile. amaury.farias@uvm.cl

10 Departamento de Química Orgánica y Fisicoquímica, Facultad de Ciencias Químicas y Farmacéuticas, Universidad de Chile, 8380492 Santiago, Chile; hpessoa@ciq.uchile.cl

11 Centro de Investigación Biomédica y Aplicada (CIBAP), Escuela de Medicina, Facultad de Ciencias Médicas, Universidad de Santiago de Chile, 9170022 Santiago, Chile; miguel.reyes@usach.cl (M-R-P)

* Correspondence: juan.gonzalez01@uautonoma.cl, Tel.: (+(56)-9-52916663); patricio.iturriaga@ufrontera.cl; Tel.: (+(56)-9-99990325)

Index.

- **Supplementary Figures – ^{1}H-NMR Spectra (compounds 4–22, Figures S1–S18), pages 3-20.**
- **Supplementary Figures – ^{13}C-NMR Spectra (compounds 7–22, Figures S19–S33), pages 21-34.**
- **High resolution mass spectrum (HRMS): (compounds 7–22, Figures S34–S48), pages 35-42.**
- **2D – NMR HSQC and HMBC compounds 7 and 12. Figures S49–S52 pages, 43-46.**
- **Biological assays - Concentration-Response Curves. Figures S53–S54, pages 47-48.**

Supplementary Figures – ^{1}H-NMR Spectra (compounds 4–22, Figures S1–S18)

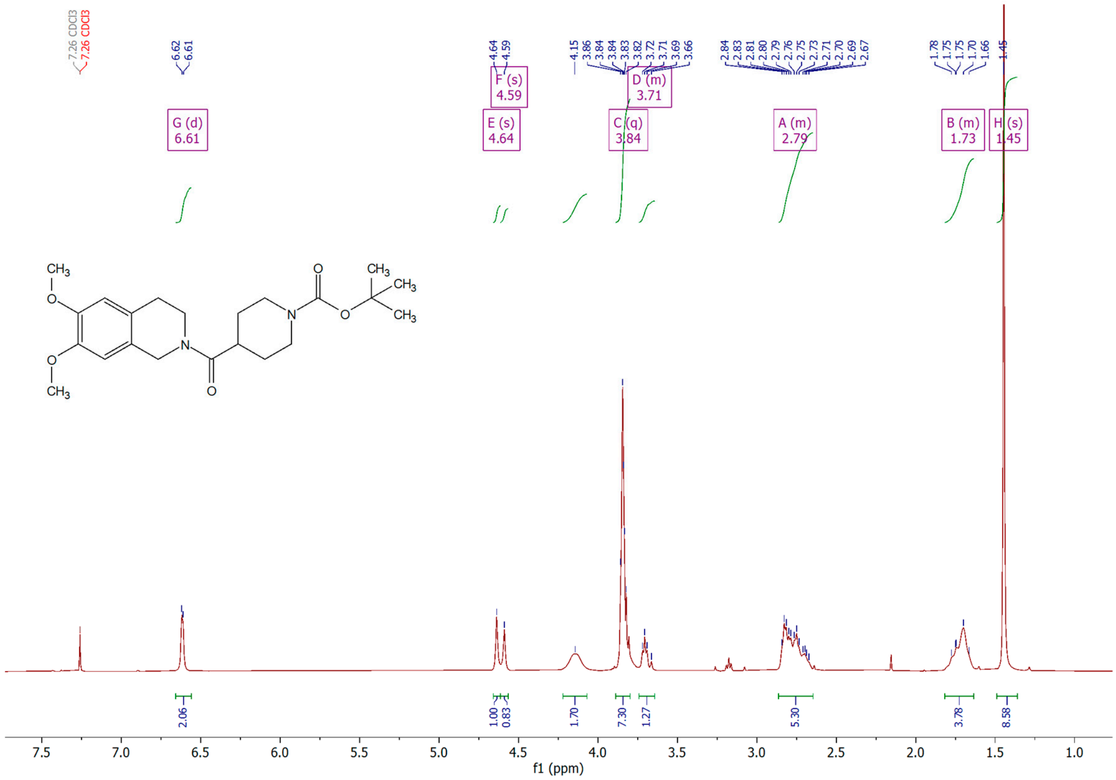

Figure S1.- ^{1}H-NMR of tert-butyl 4-(6,7-dimethoxy-1,2,3,4-tetrahydroisoquinoline-2-carbonyl)piperidine-1-carboxylate (**4**) (deuterated solvent used: $CDCl_3$).

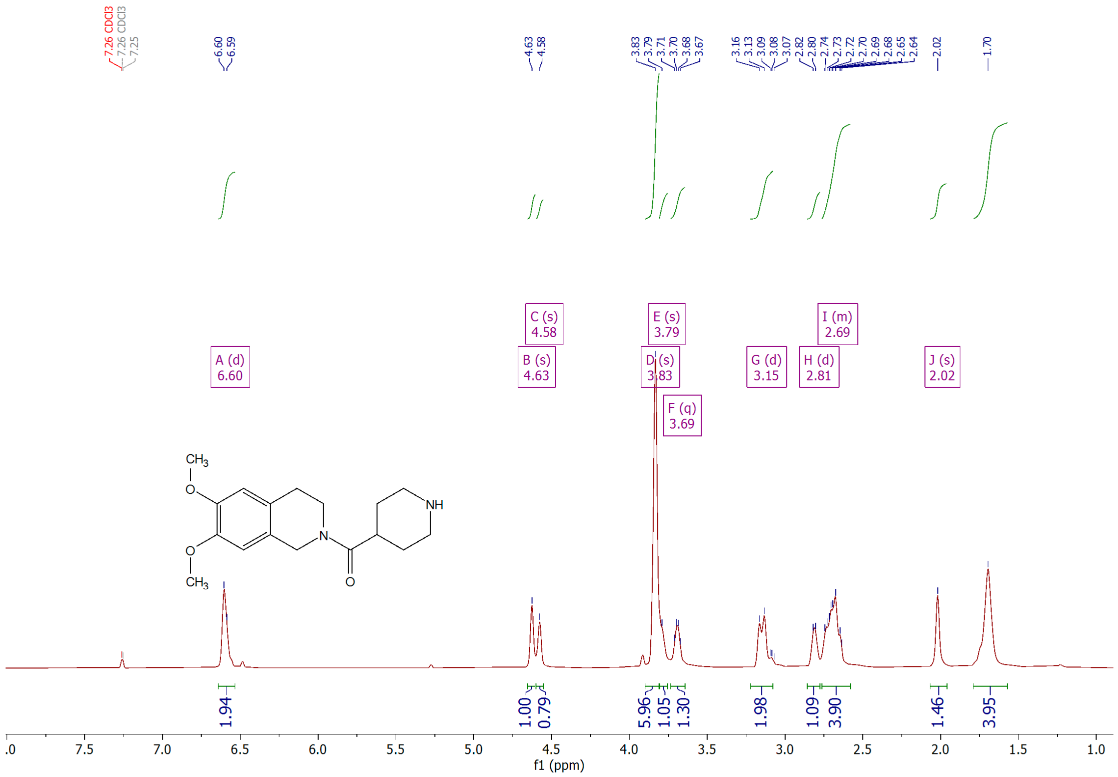

Figure S2.- ^{1}H-NMR of (6,7-dimethoxy-3,4-dihydroisoquinolin-2(1H)-yl)(piperidin-4-yl)methanone (**5**) (deuterated solvent used: $CDCl_3$).

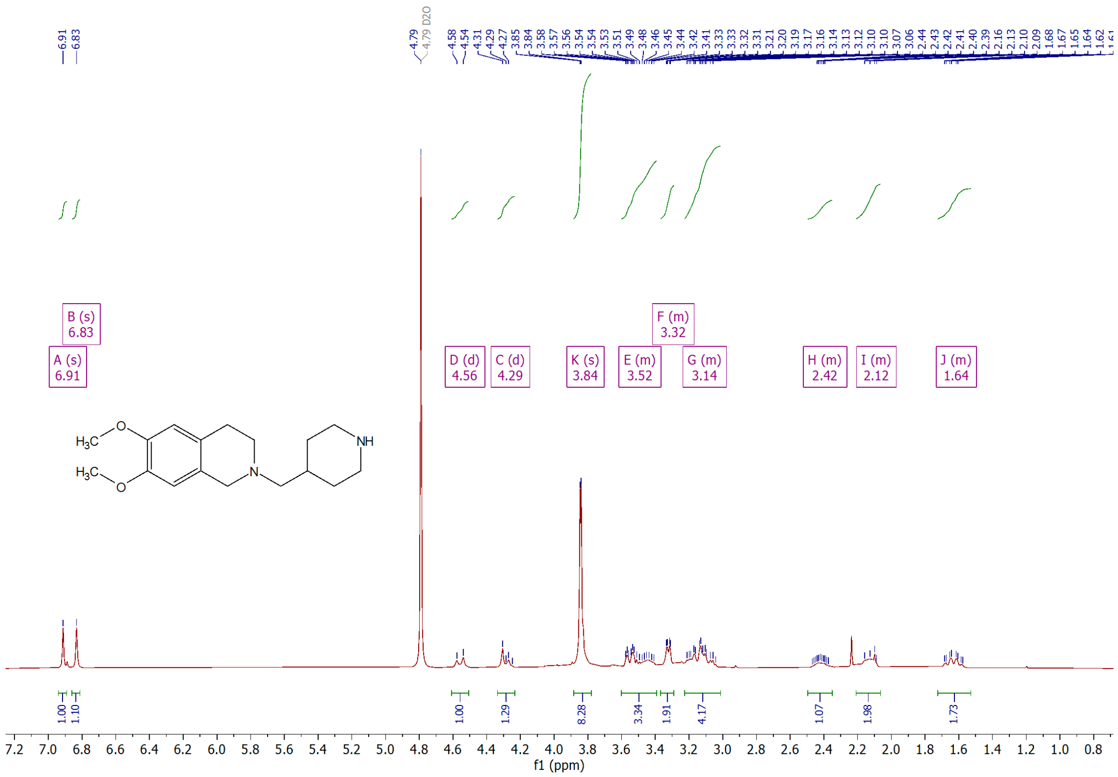

Figure S3.- ^{1}H-NMR of 6,7-dimethoxy-2-(piperidin-4-ylmethyl)-1,2,3,4-tetrahydroisoquinoline (**6**) (deuterated solvent used: D_2O).

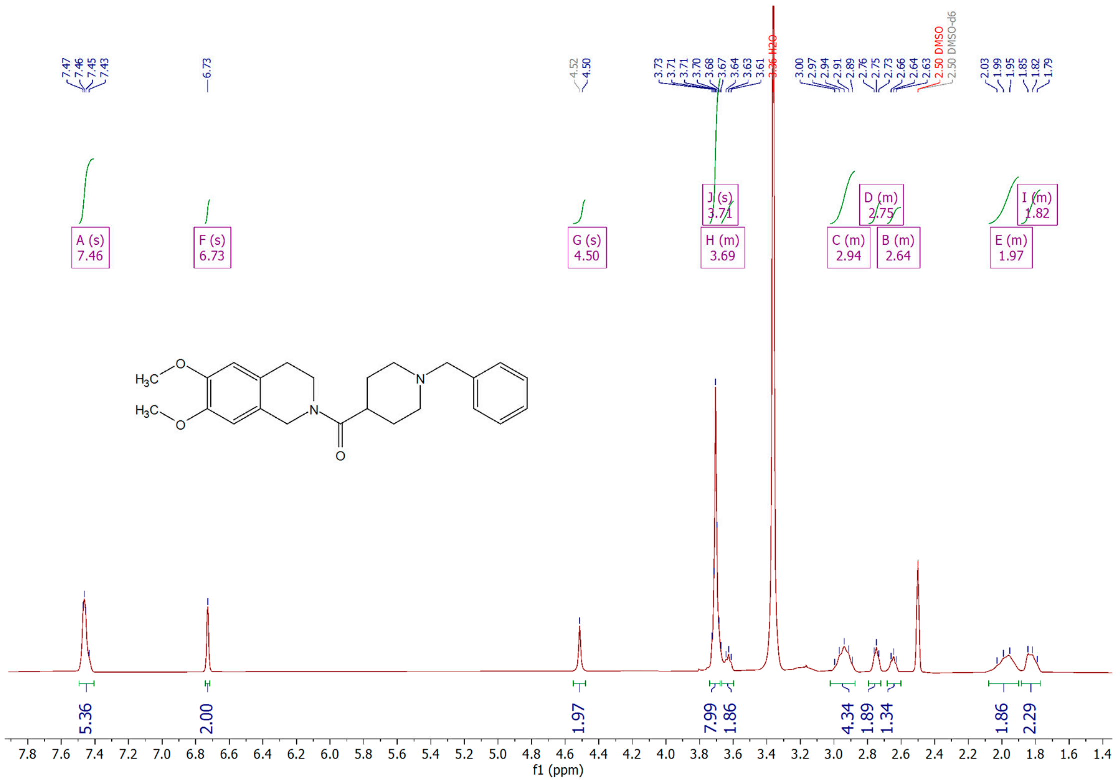

Figure S4.- ^{1}H-NMR of (1-benzylpiperidin-4-yl)(6,7-dimethoxy-3,4-dihydroisoquinolin-2(1H)-yl)methanone (**7**) (deuterated solvent used: DMSO-d_6).

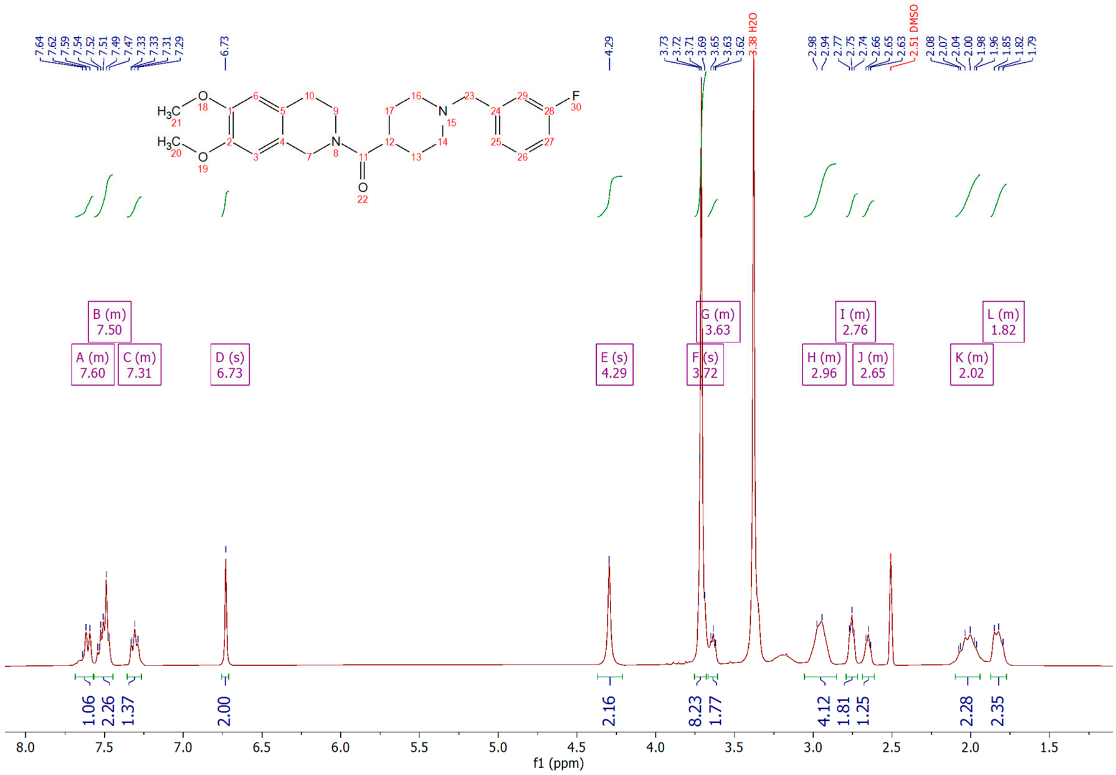

Figure S5.- ^{1}H-NMR of (6,7-dimethoxy-3,4-dihydroisoquinolin-2(1H)-yl)(1-(3-fluorobenzyl)piperidin-4-yl)methanone (**8**) (deuterated solvent used: DMSO-d_6).

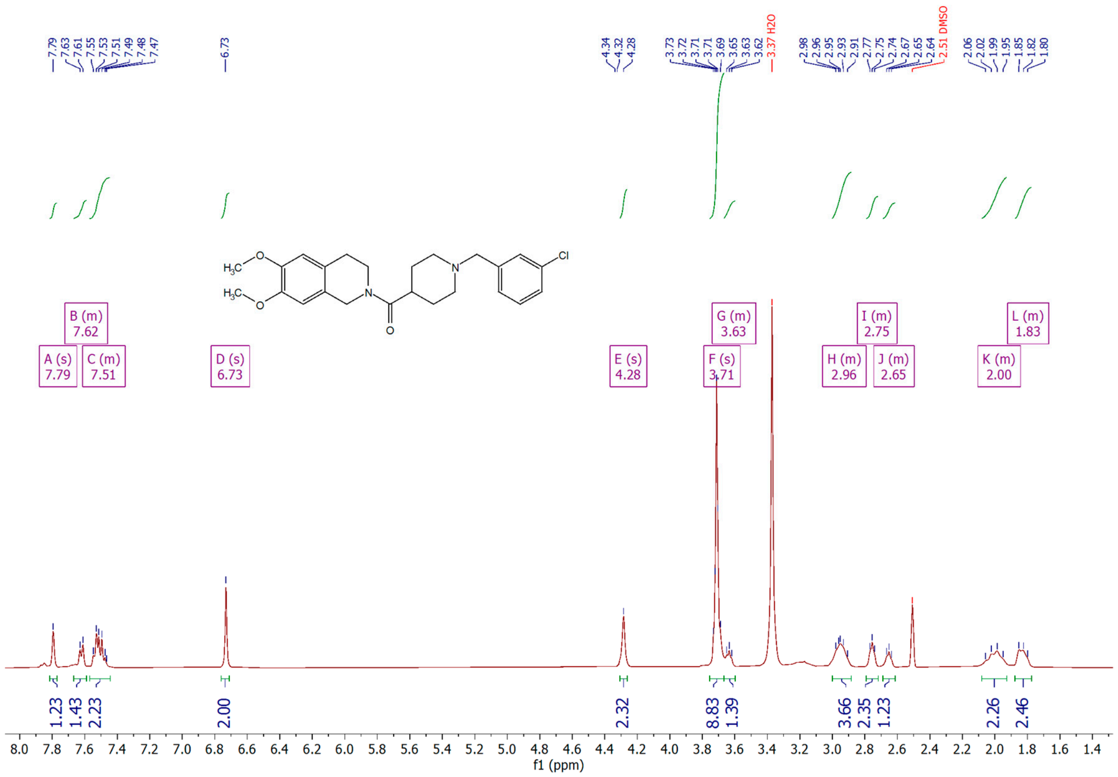

Figure S6.- ^{1}H-NMR of (1-(3-chlorobenzyl)piperidin-4-yl)(6,7-dimethoxy-3,4-dihydroisoquinolin-2(1H)-yl)methanone (**9**) (deuterated solvent used: DMSO-d_6).

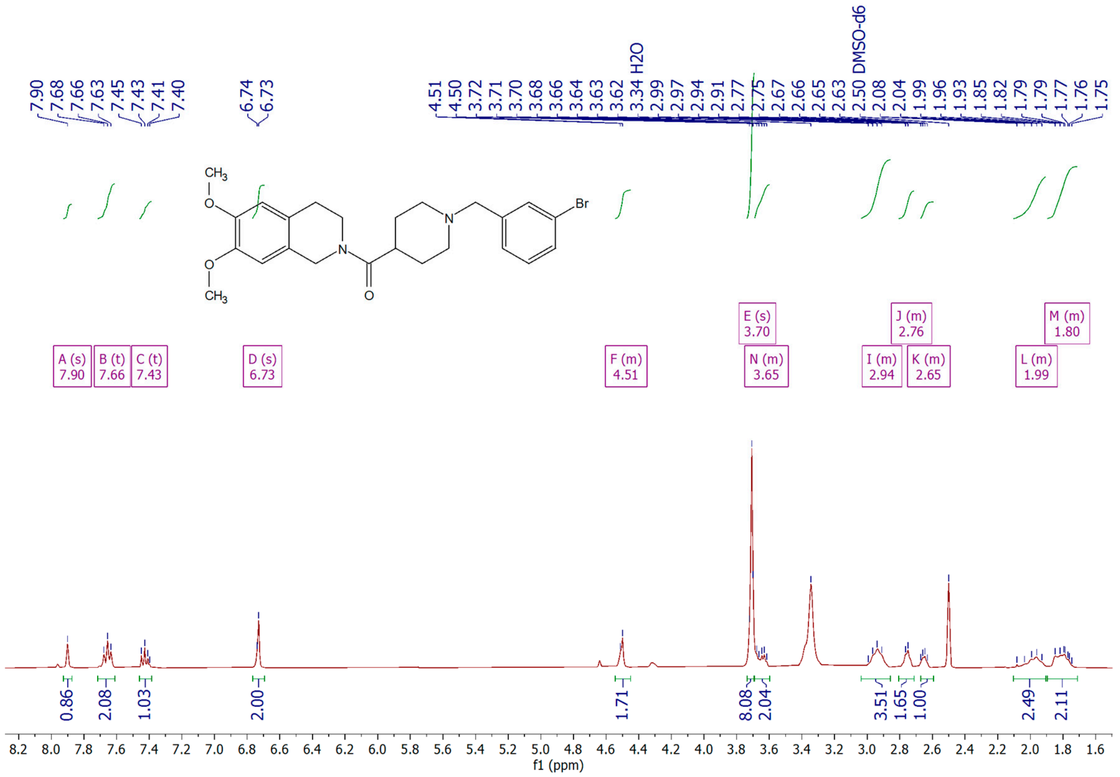

Figure S7.- ^{1}H-NMR of (1-(3-bromobenzyl)piperidin-4-yl)(6,7-dimethoxy-3,4-dihydroisoquinolin-2(1H)-yl)methanone (**10**) (deuterated solvent used: DMSO-d_6).

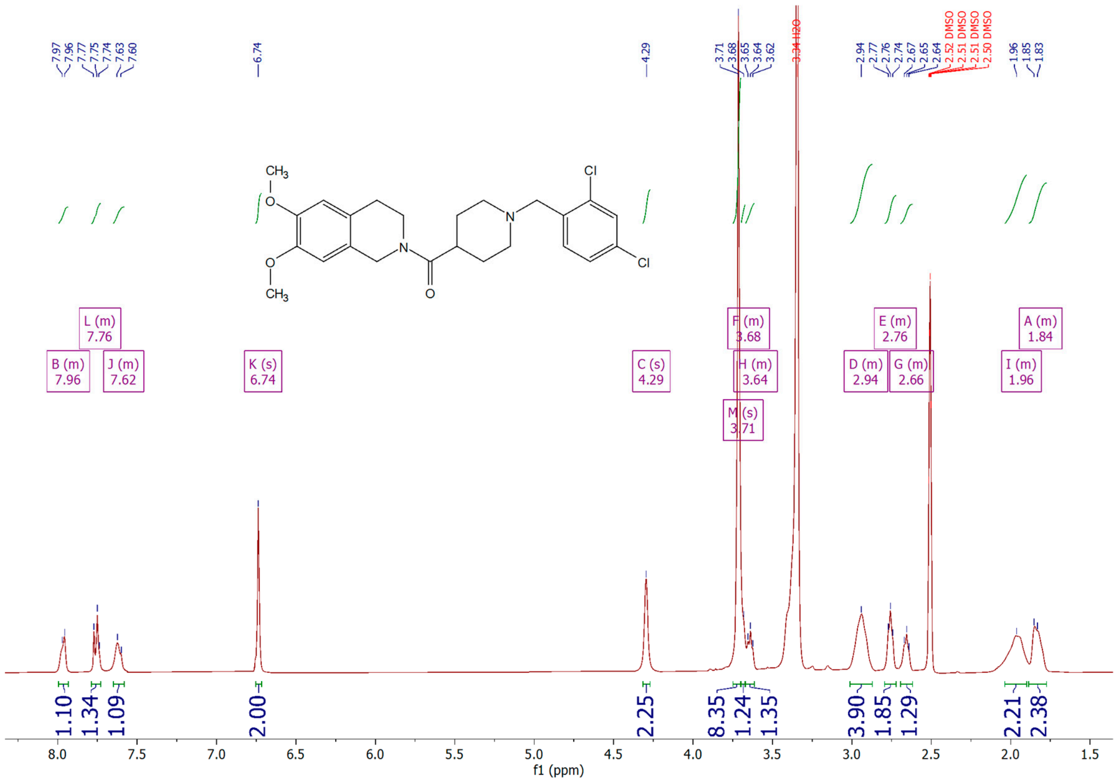

Figure S8.- ^{1}H-NMR of (1-(2,4-dichlorobenzyl)piperidin-4-yl)(6,7-dimethoxy-3,4-dihydroisoquinolin-2(1H)-yl)methanone (**11**) (deuterated solvent used: DMSO-d_6).

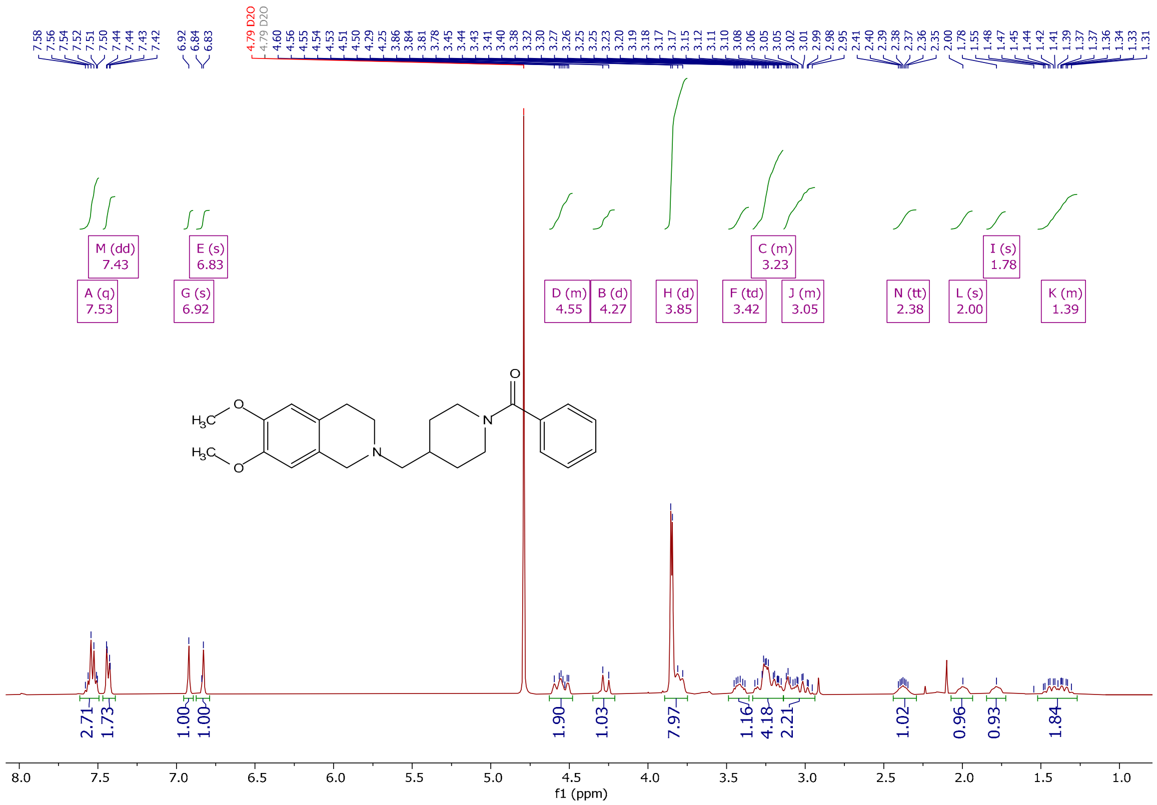

Figure S9.- ^{1}H-NMR of (4-((6,7-dimethoxy-3,4-dihydroisoquinolin-2(1H)-yl)methyl)piperidin-1-yl)(phenyl)methanone (**12**) (deuterated solvent used: D_2O).

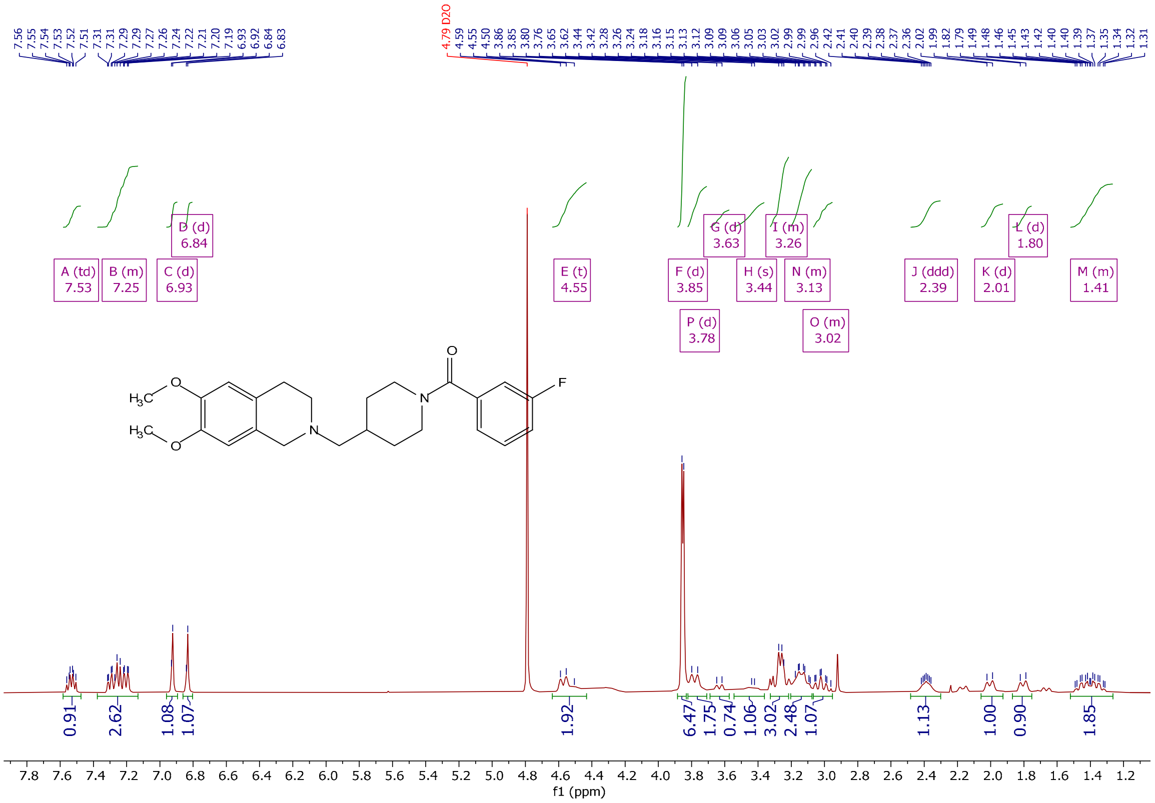

Figure S10.- ^{1}H-NMR of (4-((6,7-dimethoxy-3,4-dihydroisoquinolin-2(1H)-yl)methyl)piperidin-1-yl)(3-fluorophenyl)methanone (**13**) (deuterated solvent used: D_2O).

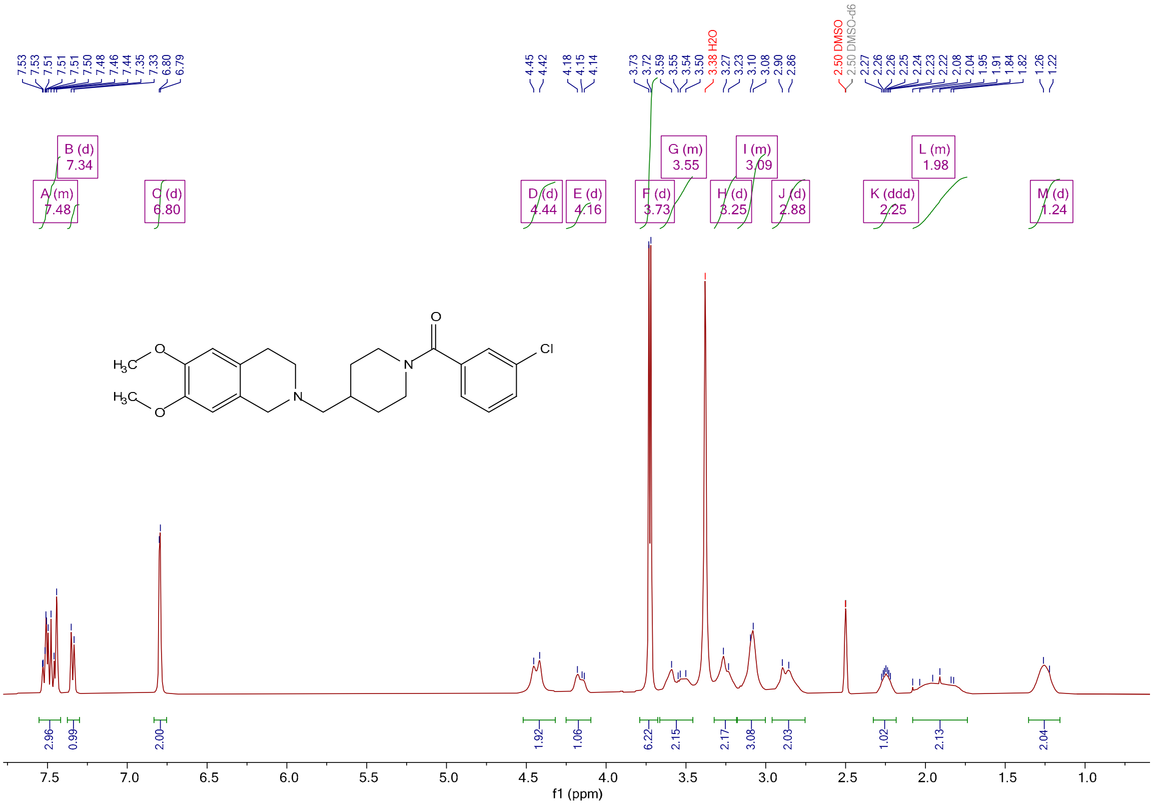

Figure S11.- ^{1}H-NMR of (3-chlorophenyl)(4-((6,7-dimethoxy-3,4-dihydroisoquinolin-2(1H)-yl)methyl)piperidin-1-yl)methanone (**14**) (deuterated solvent used: DMSO-d6).

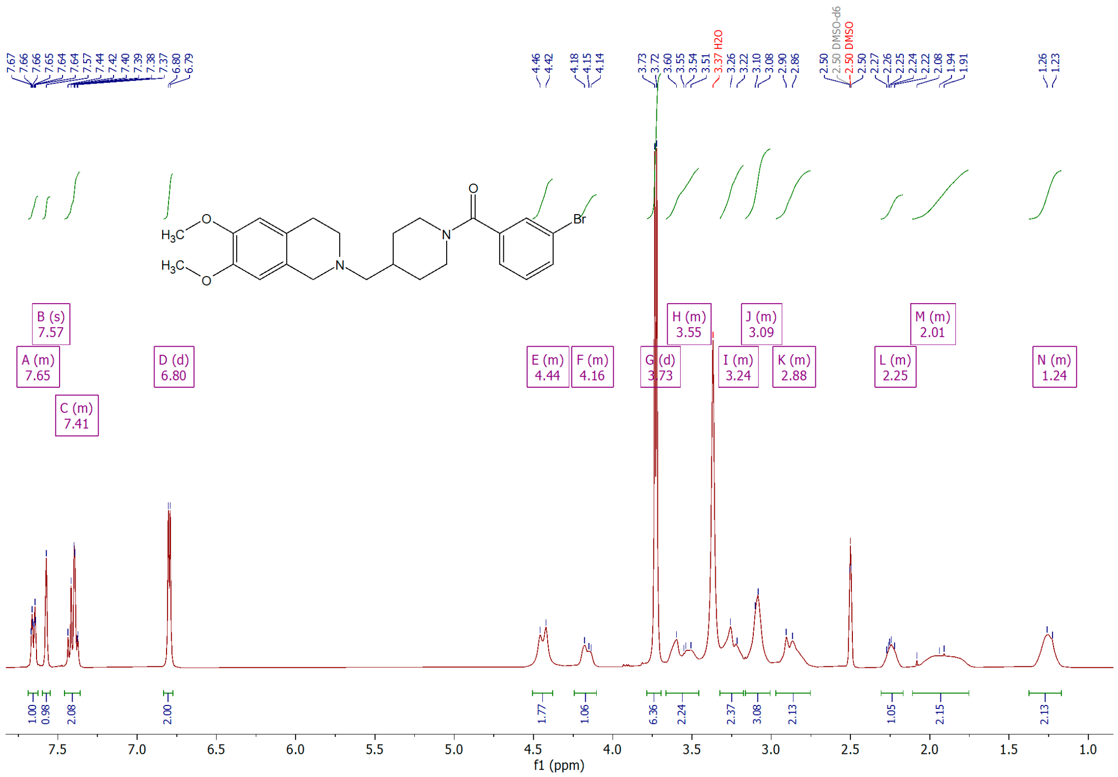

Figure S12.- ^{1}H-NMR of (3-bromophenyl)(4-((6,7-dimethoxy-3,4-dihydroisoquinolin-2(1H)-yl)methyl)piperidin-1-yl)methanone (**15**) (deuterated solvent used: DMSO-d_6).

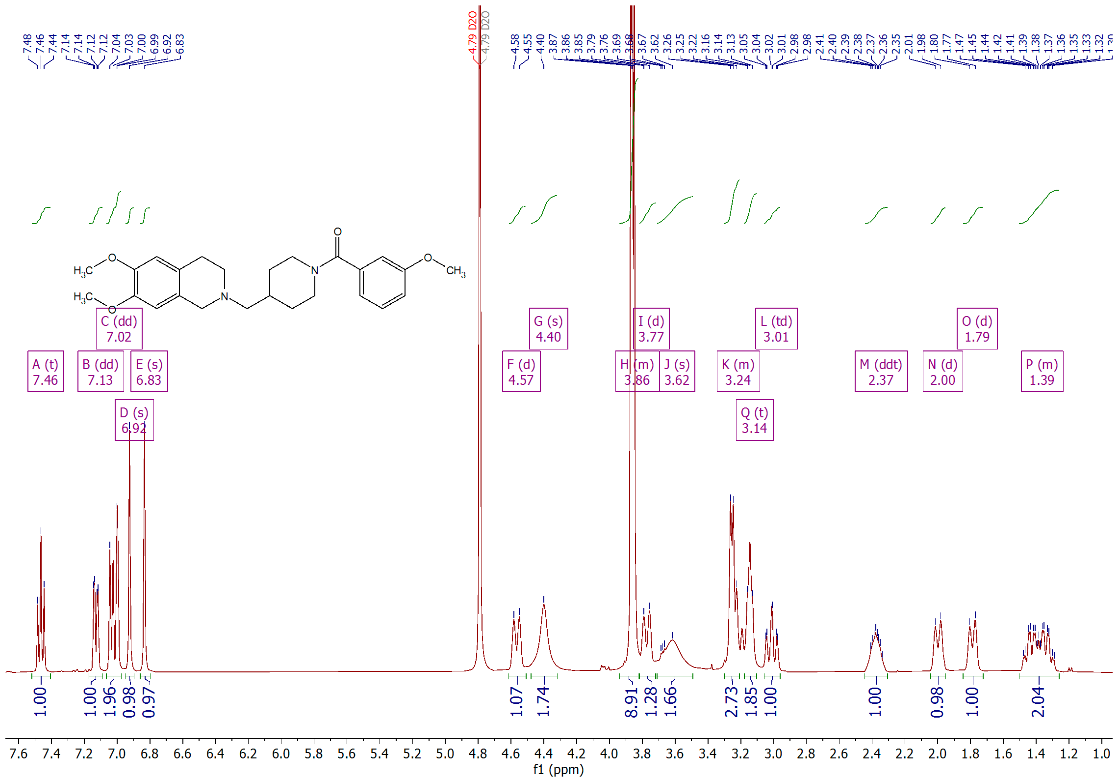

Figure S13.- ^{1}H-NMR of (4-((6,7-dimethoxy-3,4-dihydroisoquinolin-2(1H)-yl)methyl)piperidin-1-yl)(3-methoxyphenyl)methanone (**16**) (deuterated solvent used: D_2O).

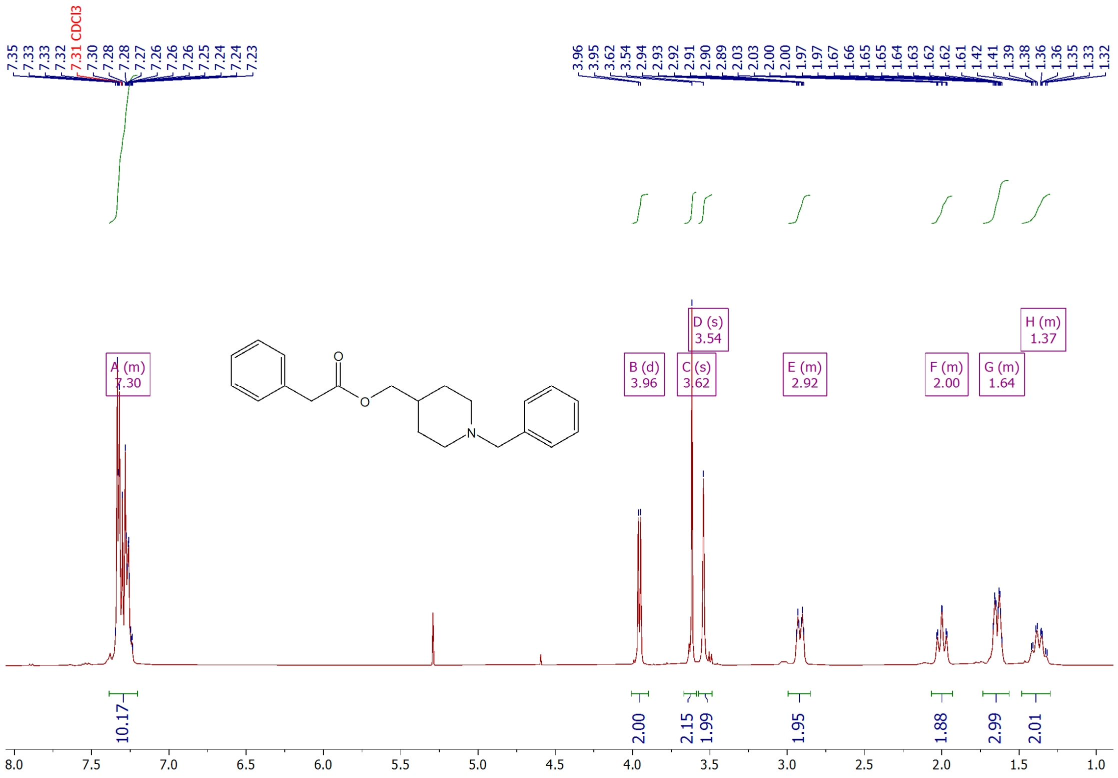

Figure S14.- ^{1}H-RMN of (1-Benzylpiperidin-4-yl)methyl 2-phenylacetate (**18**) (deuterated solvent used: $CDCl_3$).

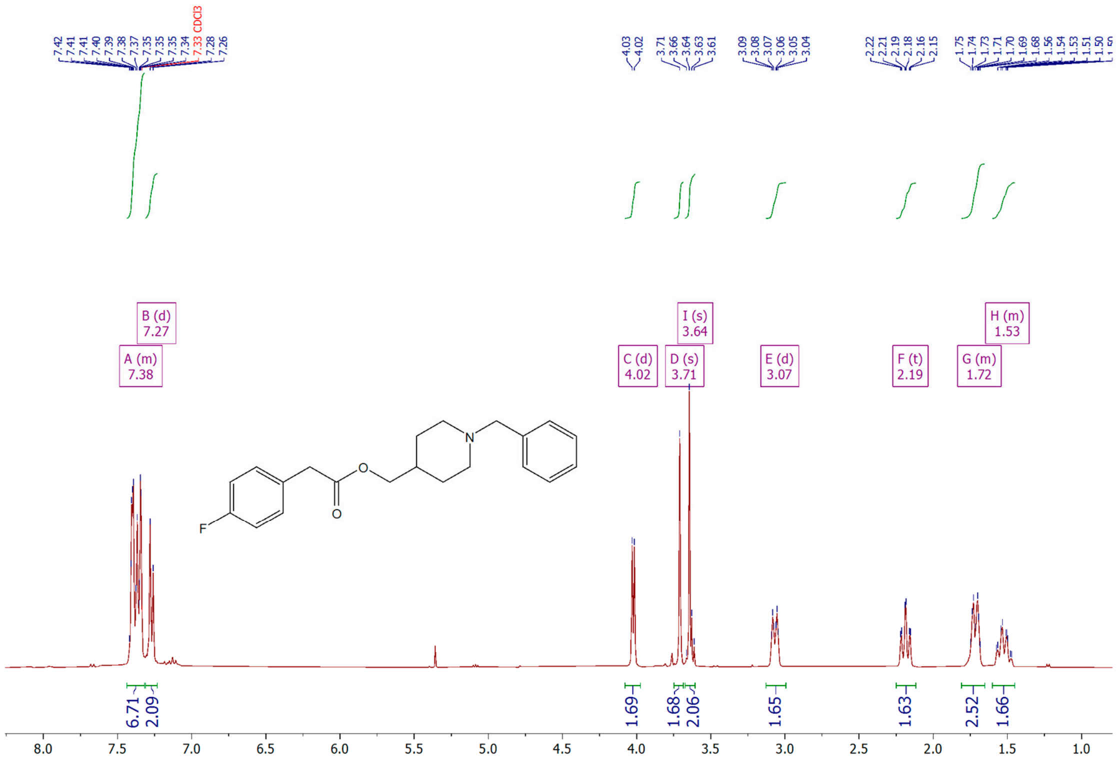

Figure S15.- ^{1}H-RMN of (1-Benzylpiperidin-4-yl)methyl 2-(4-fluorophenyl)acetate (**19**) (deuterated solvent used: $CDCl_3$).

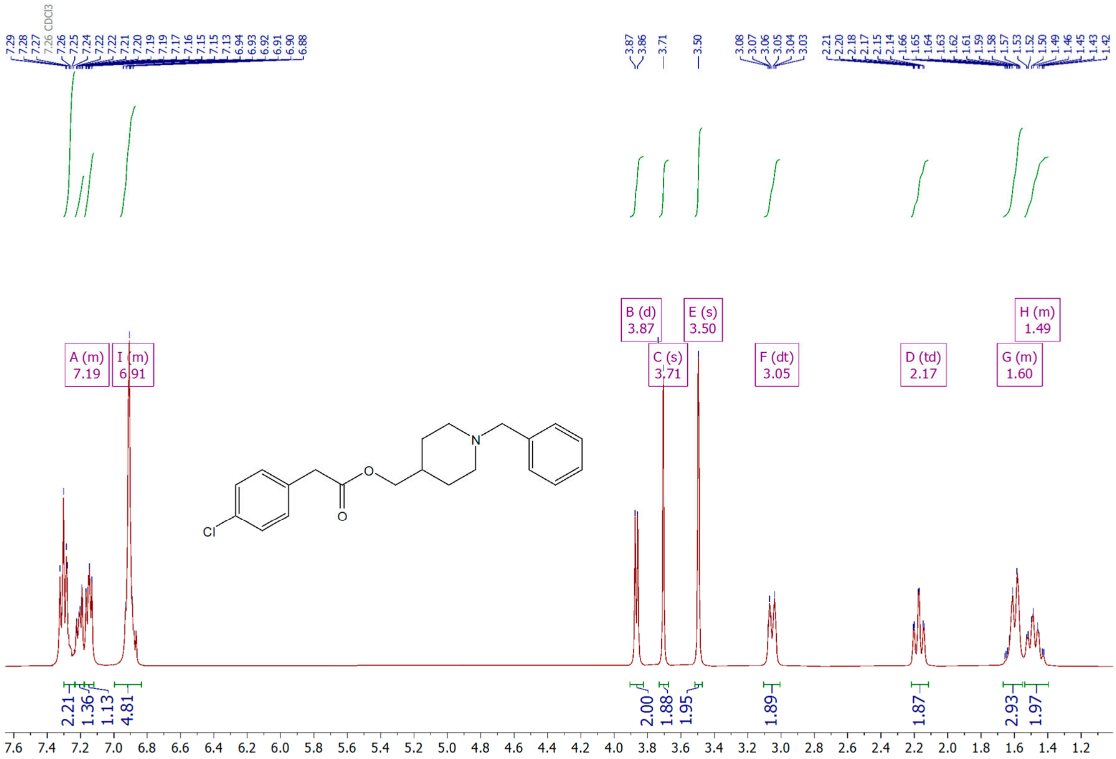

Figure S16.- ^{1}H-RMN of (1-Benzylpiperidin-4-yl)methyl 2-(4-chlorophenyl)acetate (**20**) (deuterated solvent used: $CDCl_3$).

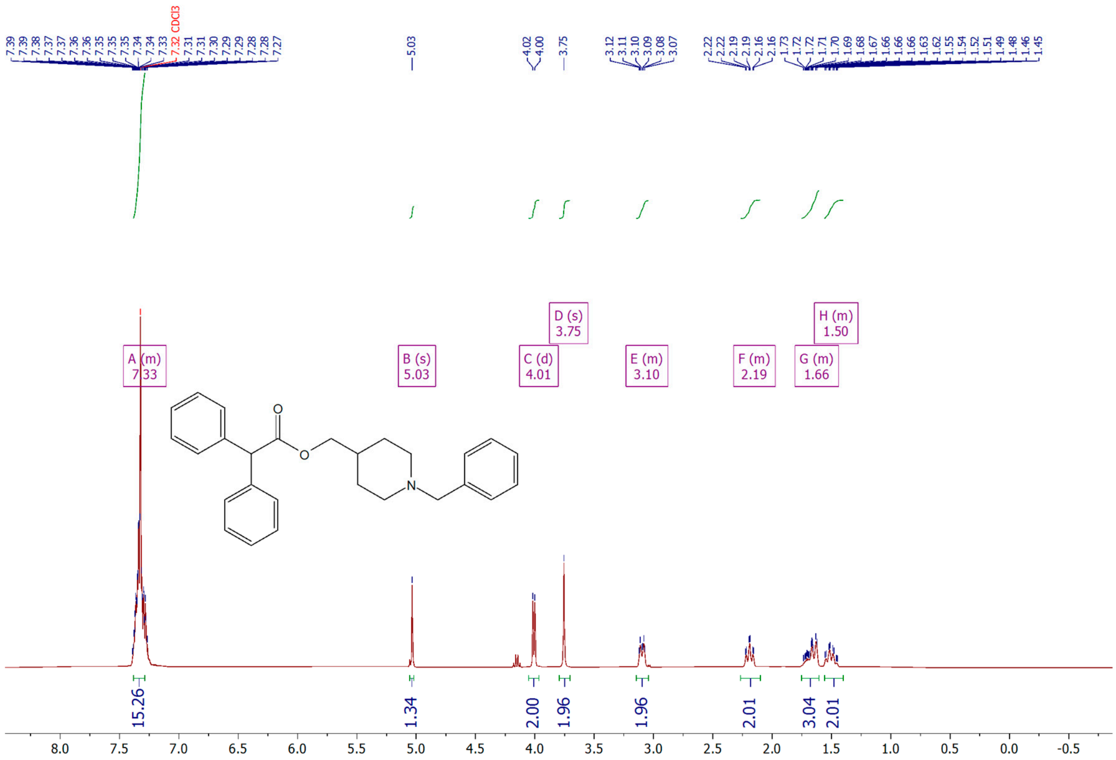

Figure S17.- ^{1}H-RMN of (1-Benzylpiperidin-4-yl)methyl 2,2-diphenylacetate (**21**) (deuterated solvent used: $CDCl_3$).

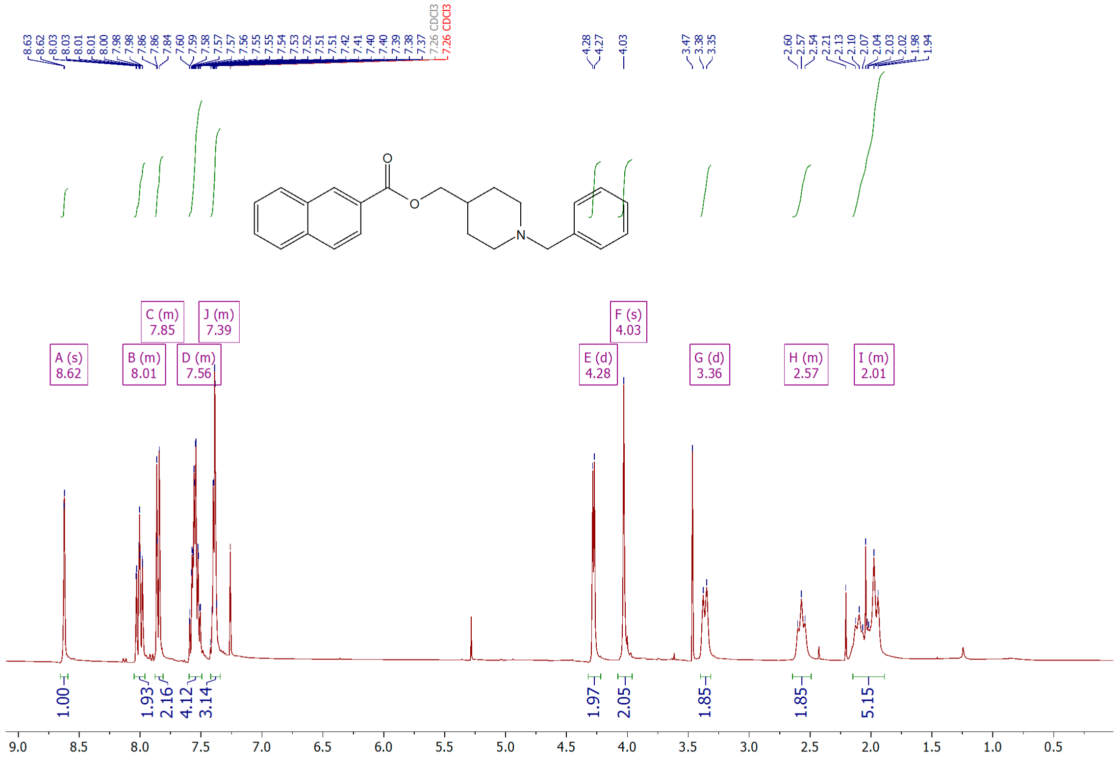

Figure S18.- ^{1}H-RMN of (1-Benzylpiperidin-4-yl)methyl 2-naphthoate (**22**) (deuterated solvent used: $CDCl_3$).

Supplementary Figures – ^{13}C-NMR Spectra (compounds 7–22, Figures S19–S33)

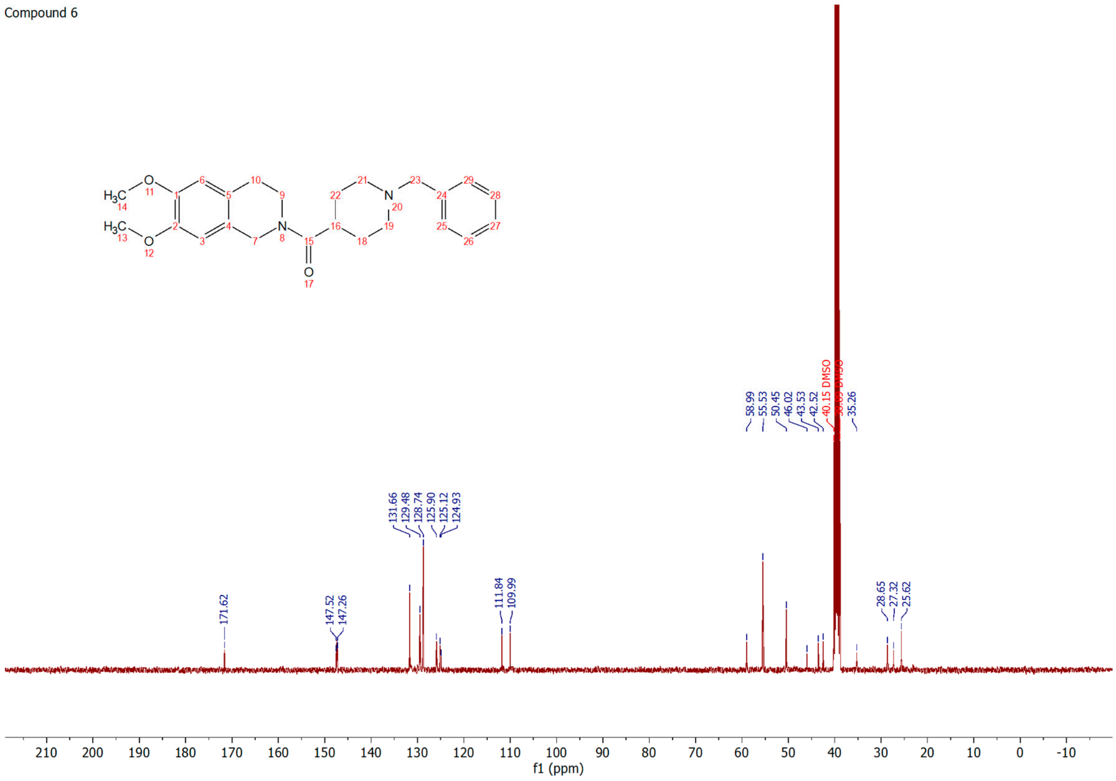

Figure S19.- ^{13}C-NMR of (1-benzylpiperidin-4-yl)(6,7-dimethoxy-3,4-dihydroisoquinolin-2(1H)-yl)methanone (**7**) (deuterated solvent used: DMSO-d$_6$).

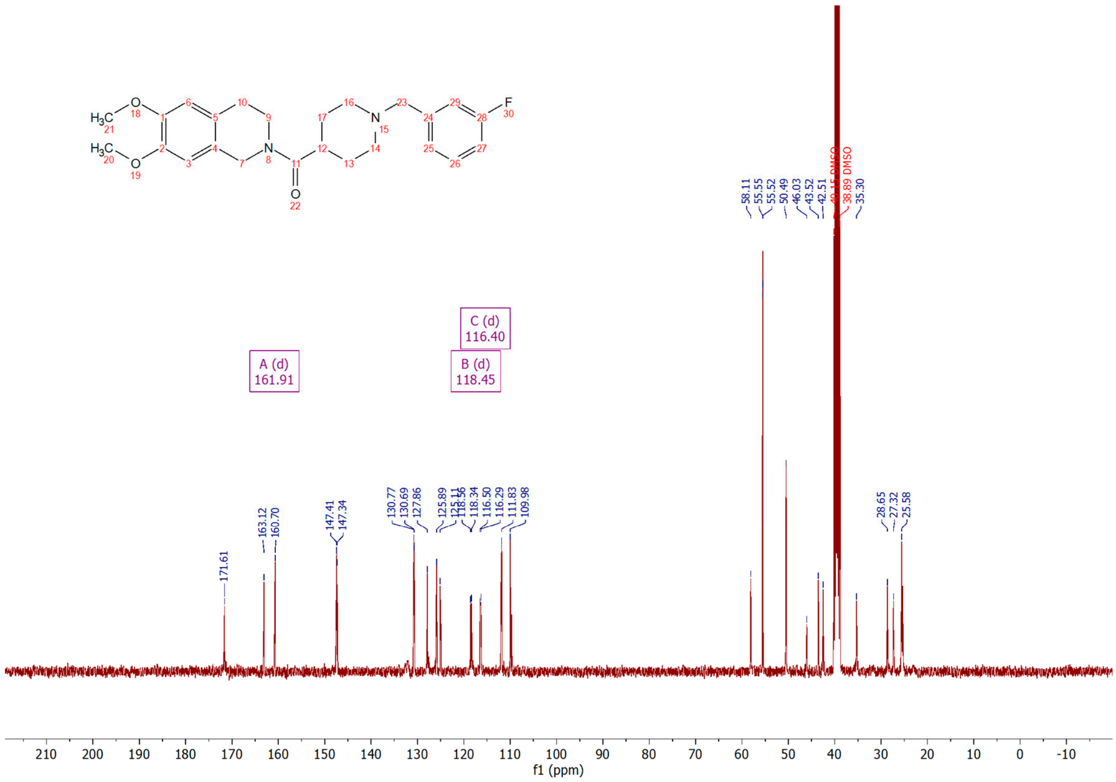

Figure S20.- ^{13}C-NMR of (6,7-dimethoxy-3,4-dihydroisoquinolin-2(1H)-yl)(1-(3-fluorobenzyl)piperidin-4-yl)methanone (**8**) (deuterated solvent used: DMSO-d_6).

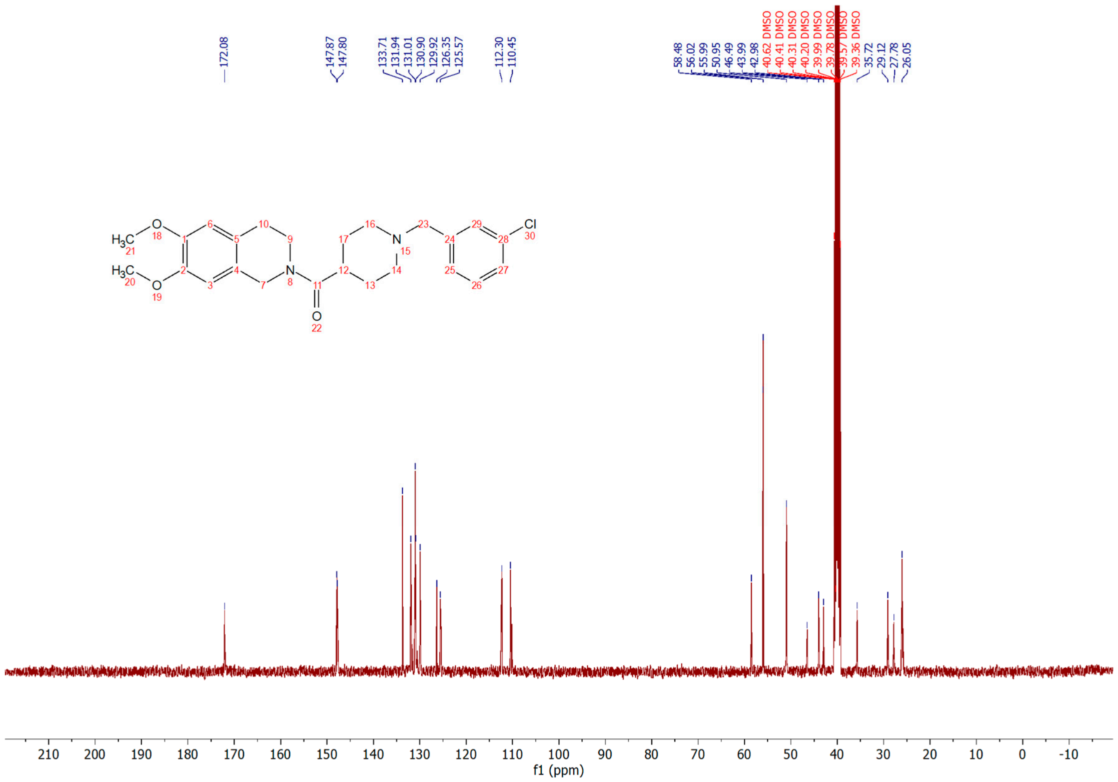

Figure S21.- ^{13}C-NMR of (1-(3-chlorobenzyl)piperidin-4-yl)(6,7-dimethoxy-3,4-dihydroisoquinolin-2(1H)-yl)methanone (**9**) (deuterated solvent used: DMSO-d_6).

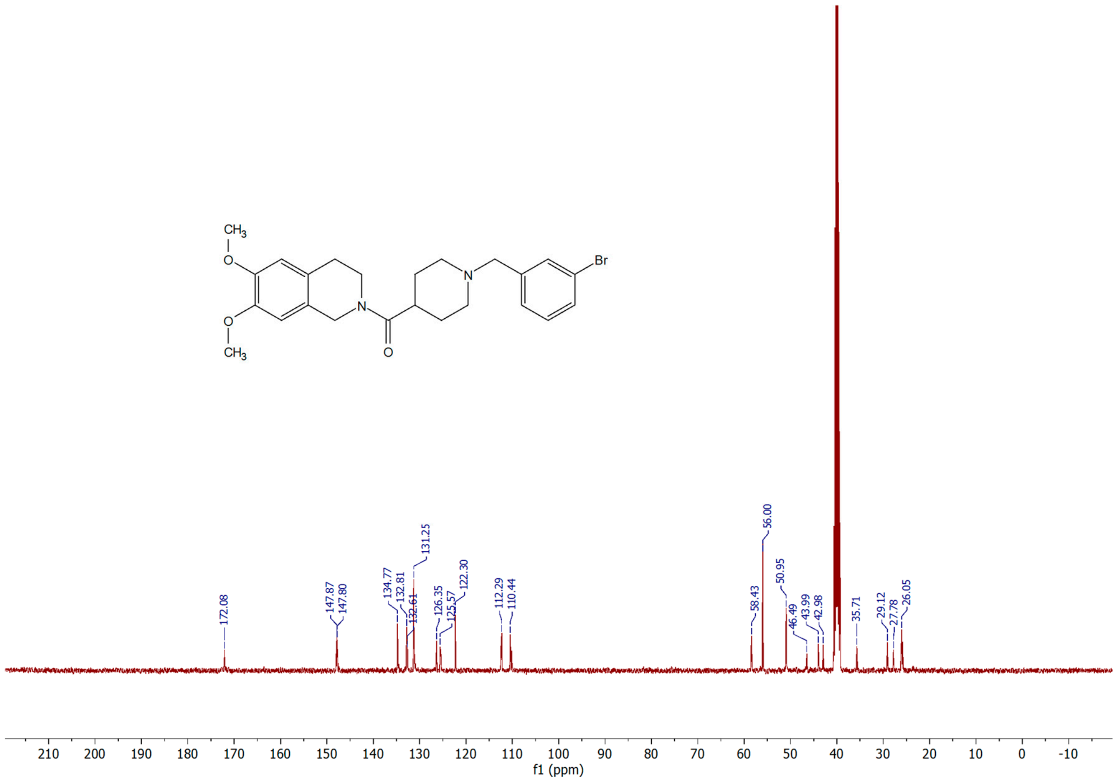

Figure S22.- ^{13}C-NMR of (1-(3-bromobenzyl)piperidin-4-yl)(6,7-dimethoxy-3,4-dihydroisoquinolin-2(1H)-yl)methanone (**10**). (deuterated solvent used: D_2O).

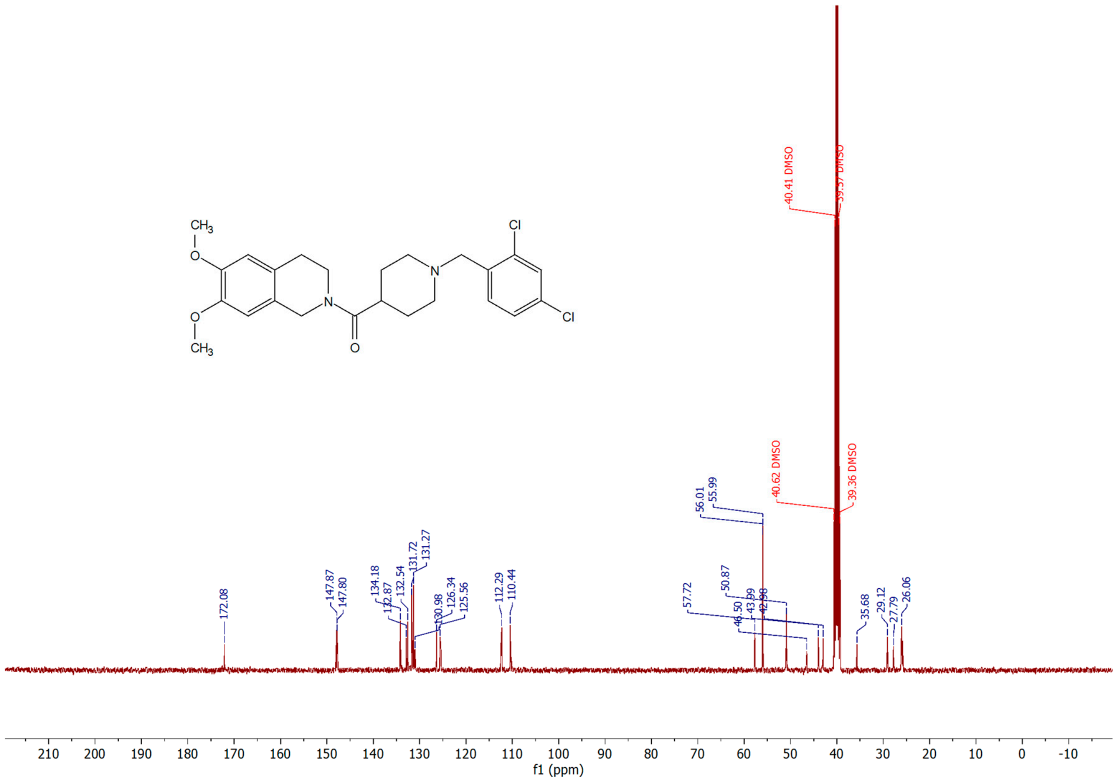

Figure S23.- ^{13}C-NMR of (1-(2,4-dichlorobenzyl)piperidin-4-yl)(6,7-dimethoxy-3,4-dihydroisoquinolin-2(1H)-yl)methanone (**11**). (deuterated solvent used: DMSO-d_6).

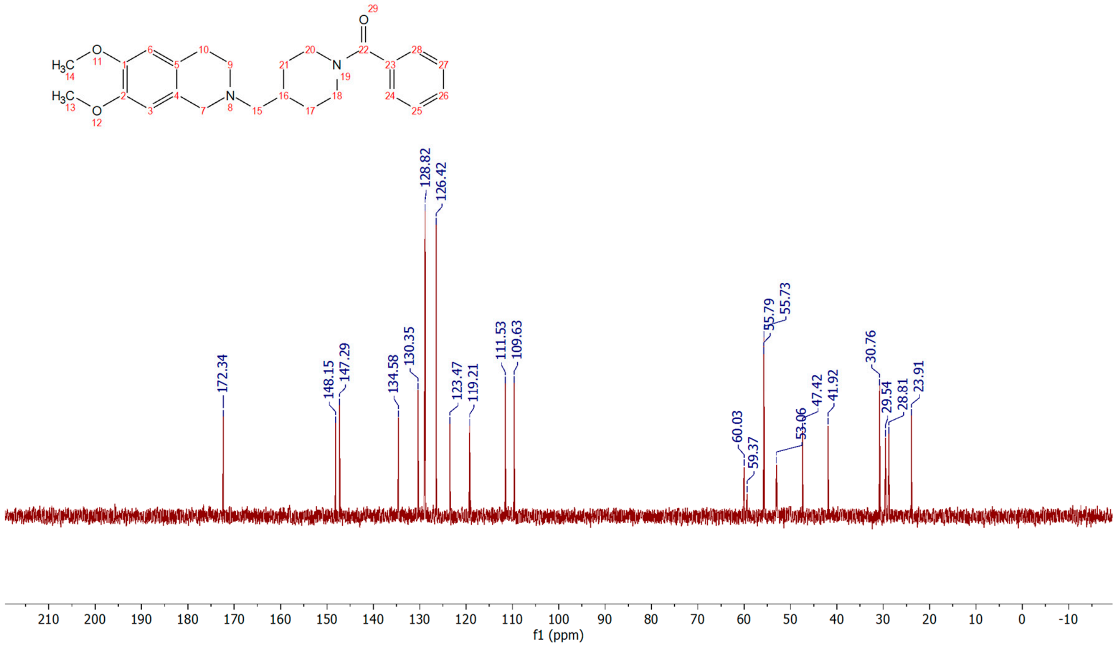

Figure S24.- ^{13}C-NMR of (4-((6,7-dimethoxy-3,4-dihydroisoquinolin-2(1H)-yl)methyl)piperidin-1-yl)(phenyl)methanone (**12**) (deuterated solvent used: D_2O).

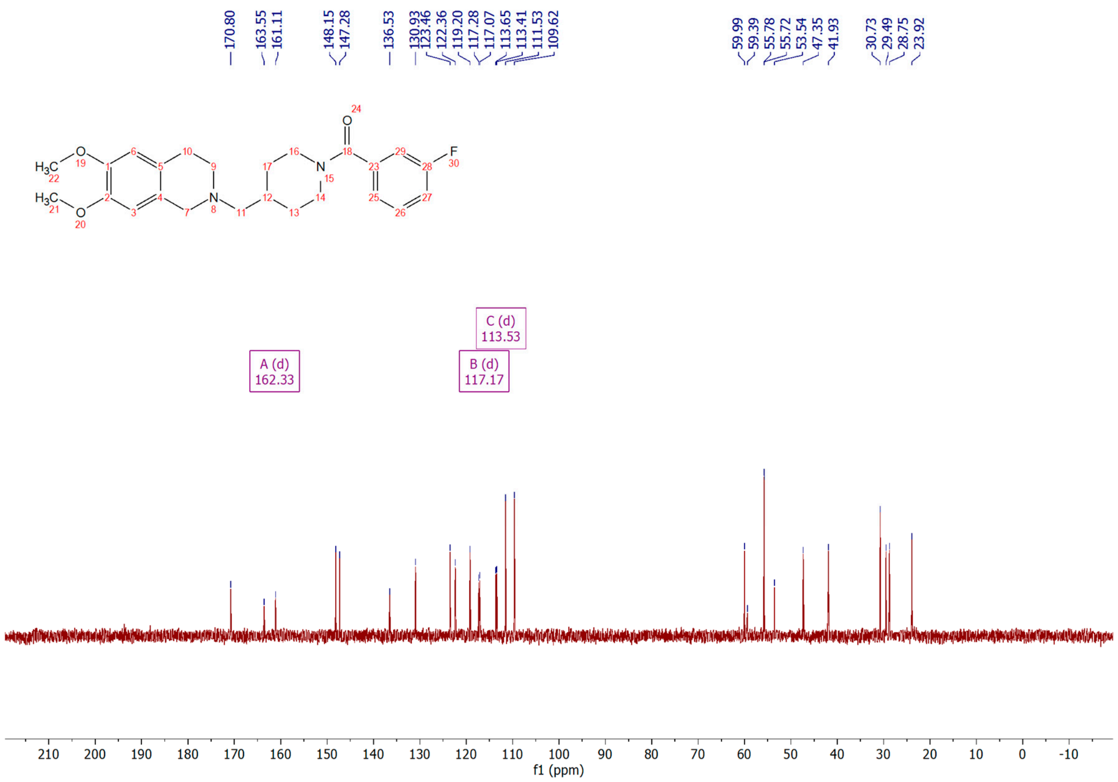

Figure S25.- ^{13}C-NMR of (4-((6,7-dimethoxy-3,4-dihydroisoquinolin-2(1H)-yl)methyl)piperidin-1-yl)(3-fluorophenyl)methanone (**13**) (deuterated solvent used: D_2O).

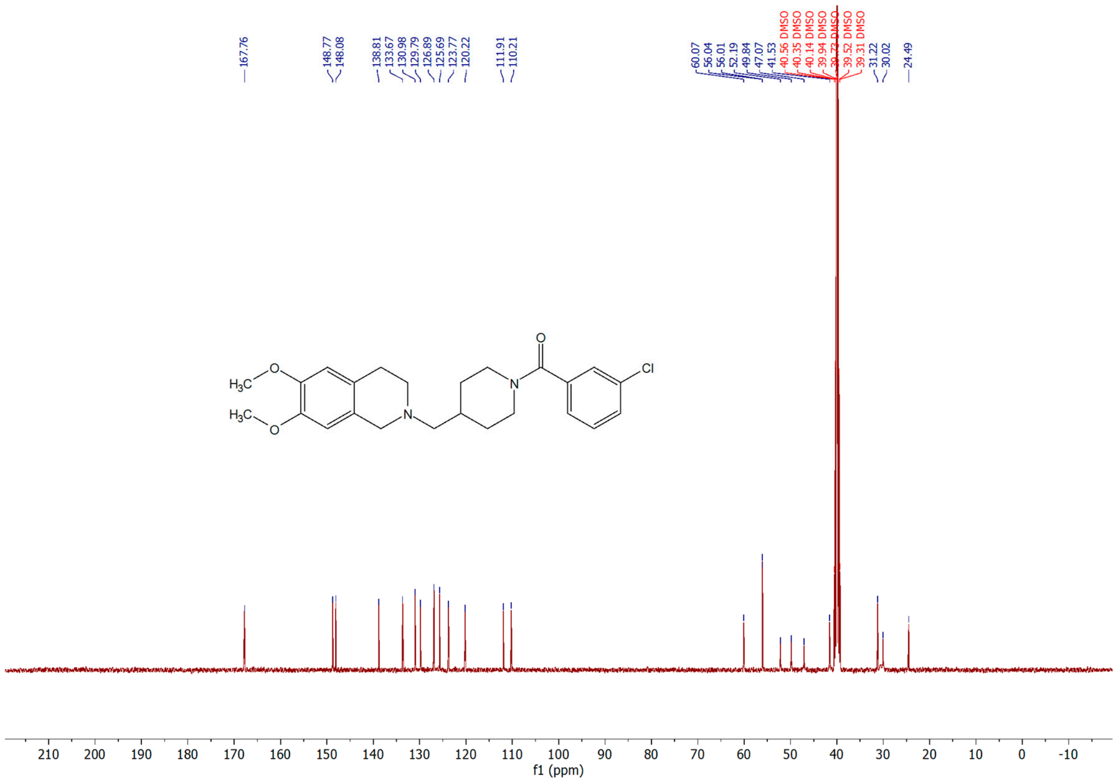

Figure S26.- ^{13}C-NMR of (3-chlorophenyl)(4-((6,7-dimethoxy-3,4-dihydroisoquinolin-2(1H)-yl)methyl)piperidin-1-yl)methanone (**14**) (deuterated solvent used: DMSO-d_6).

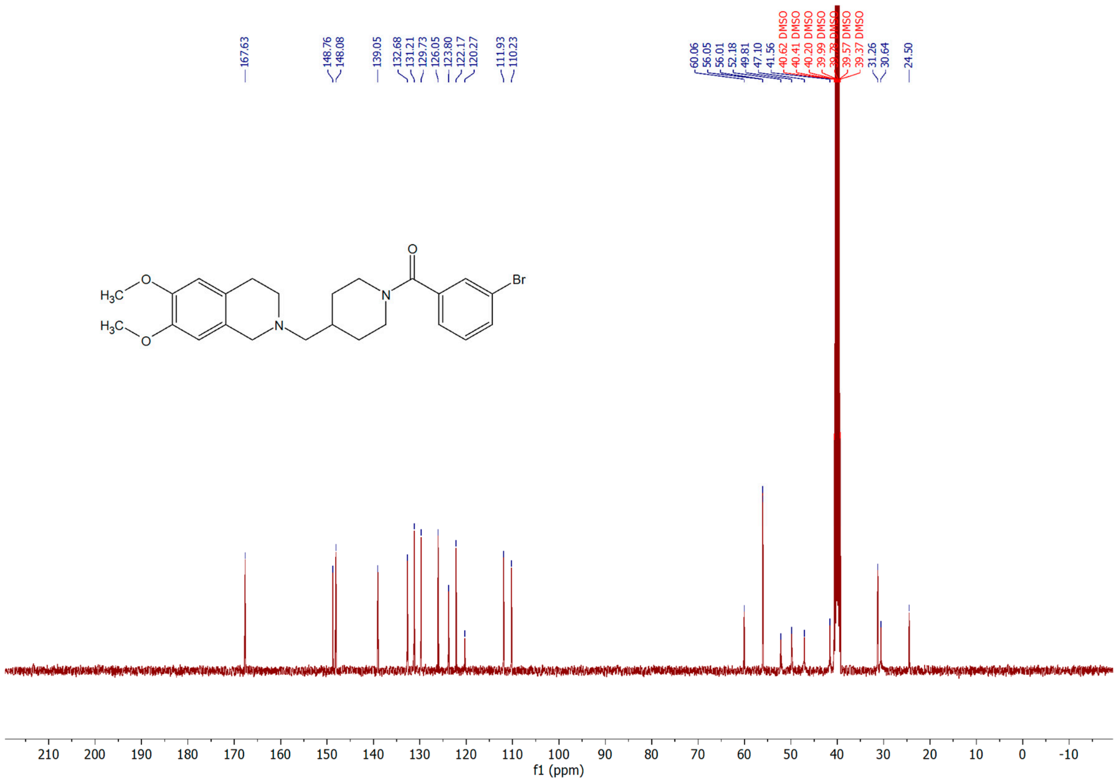

Figure S27.- ^{13}C-NMR of (3-bromophenyl)(4-((6,7-dimethoxy-3,4-dihydroisoquinolin-2(1H)-yl)methyl)piperidin-1-yl)methanone (**15**) (deuterated solvent used: DMSO-d_6).

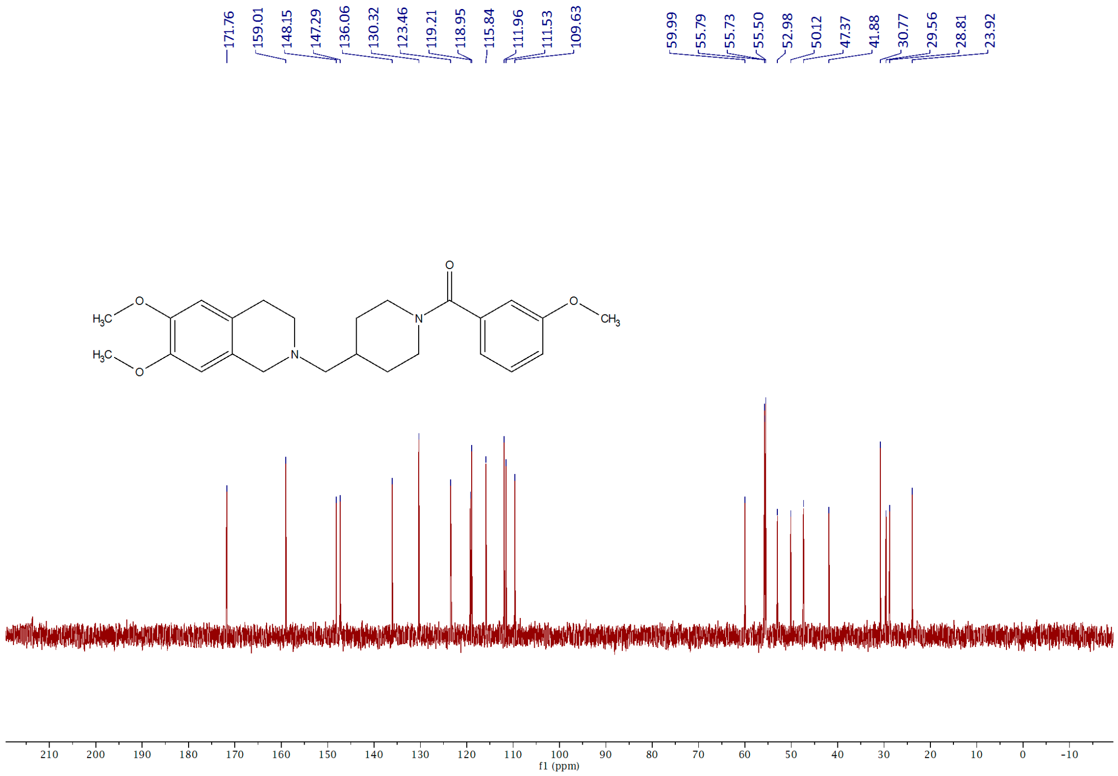

Figure S28.- ^{13}C-NMR of (4-((6,7-dimethoxy-3,4-dihydroisoquinolin-2(1H)-yl)methyl)piperidin-1-yl)(3-methoxyphenyl)methanone (**16**) (deuterated solvent used: D_2O).

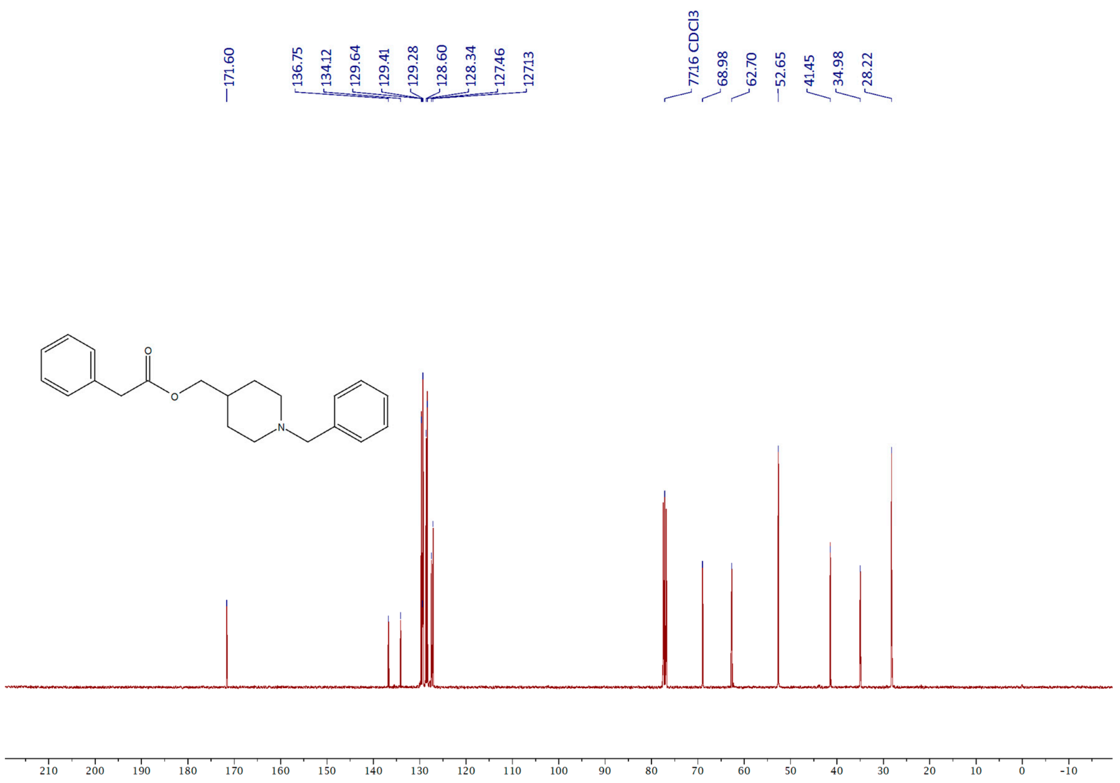

Figure S29.- ^{13}C-RMN of (1-Benzylpiperidin-4-yl)methyl 2-phenylacetate (**18**) (deuterated solvent used: $CDCl_3$).

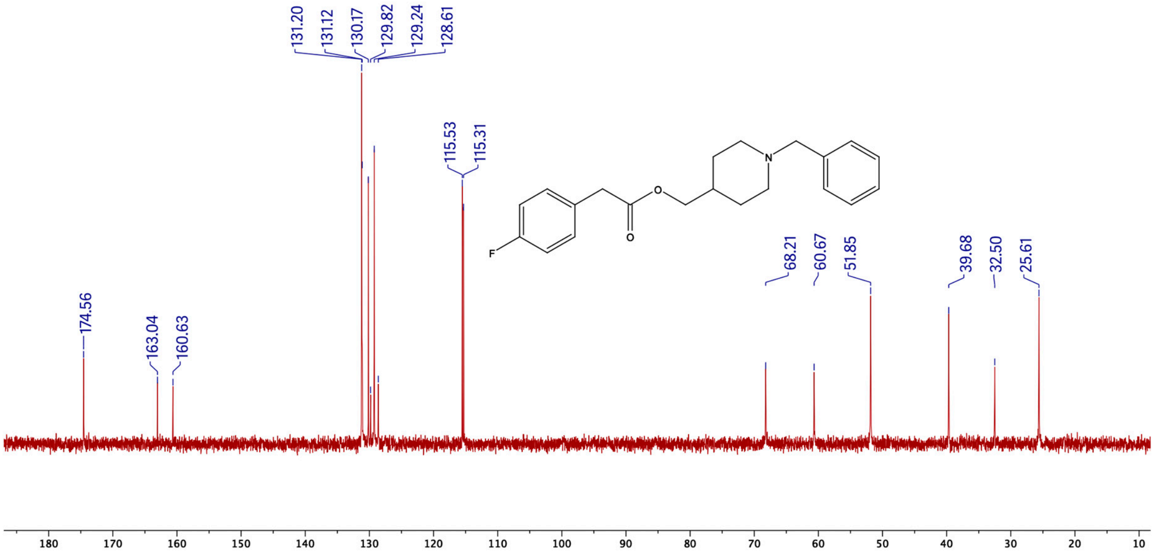

Figure S30.- ^{13}C-RMN of (1-Benzylpiperidin-4-yl)methyl 2-(4-fluorophenyl)acetate (**19**) (deuterated solvent used: D_2O).

BPB3 13C

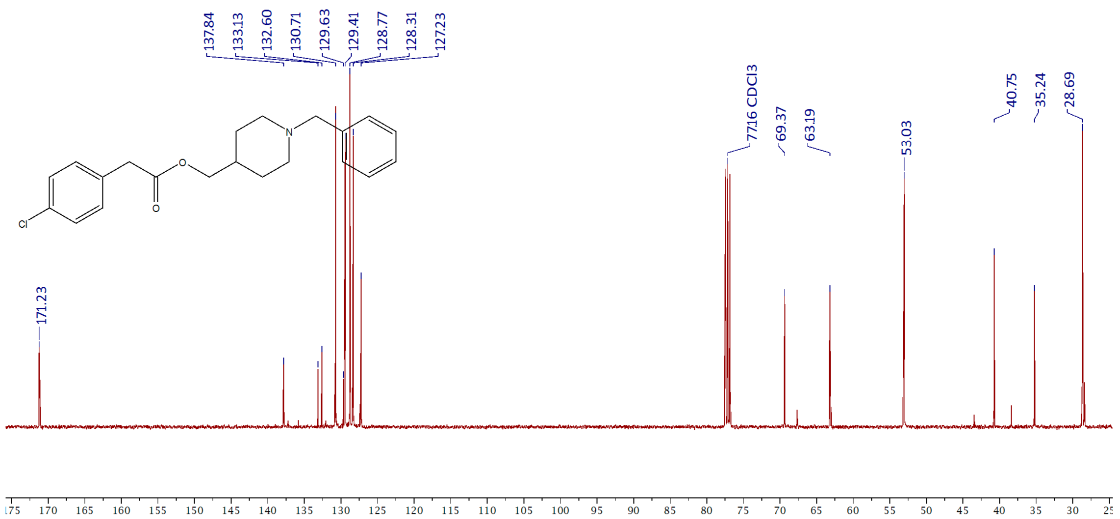

Figure S31.- ^{13}C-RMN of (1-Benzylpiperidin-4-yl)methyl 2-(4-chlorophenyl)acetate (**20**) (deuterated solvent used: $CDCl_3$).

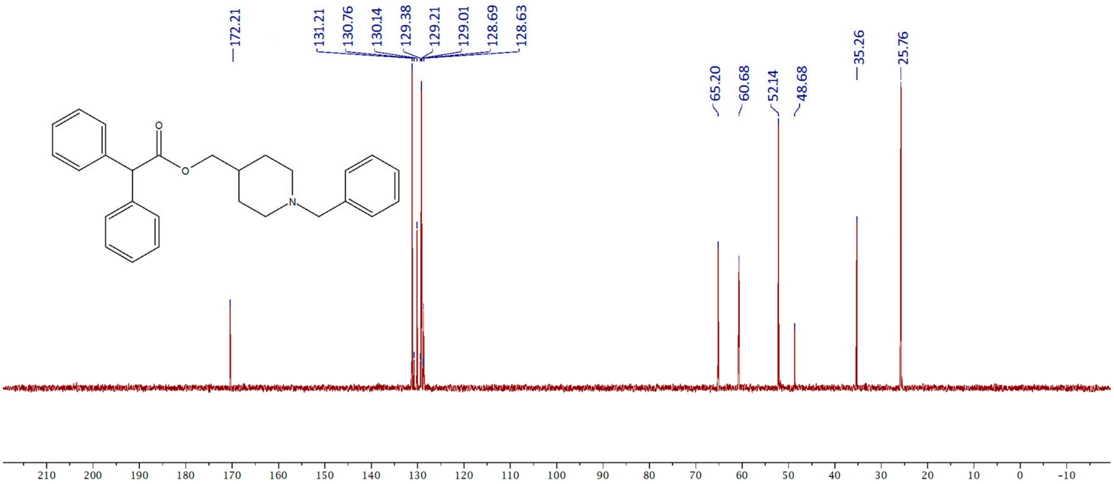

Figure S32.- ^{13}C-RMN of (1-Benzylpiperidin-4-yl)methyl 2,2-diphenylacetate (**21**) (deuterated solvent used: D_2O).

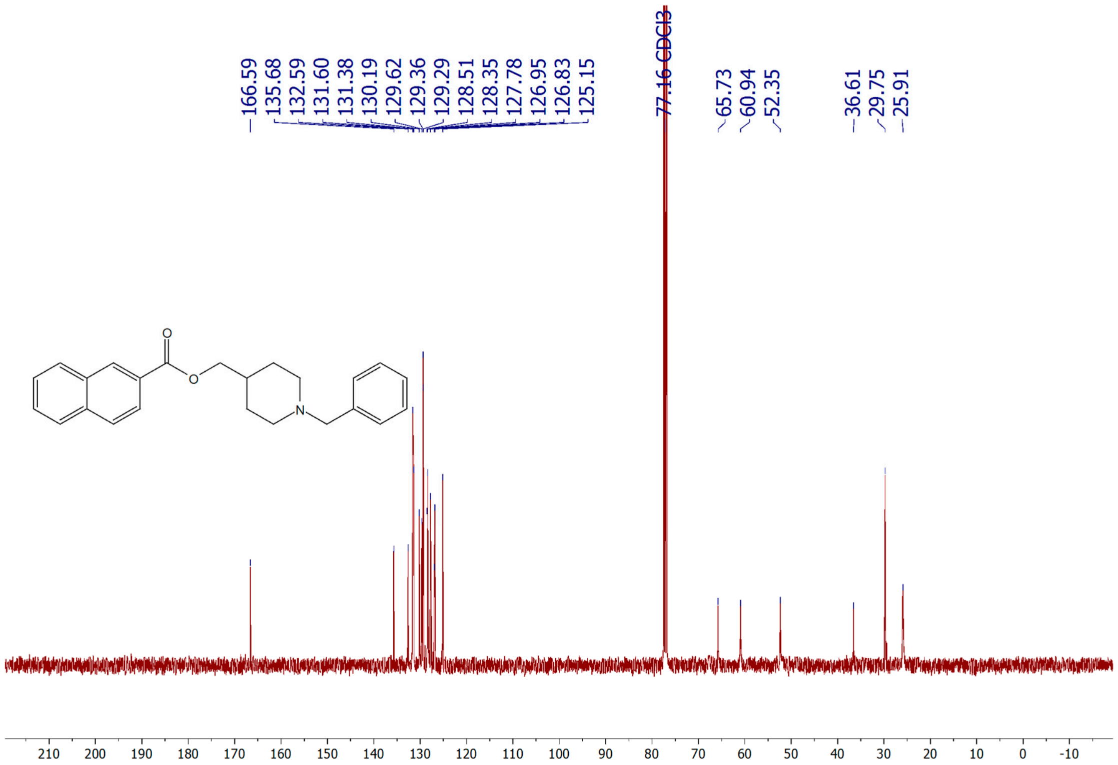

Figure S33.- ^{13}C-RMN of (1-Benzylpiperidin-4-yl)methyl 2-naphthoate (**22**) (deuterated solvent used: $CDCl_3$).

High resolution mass spectrum (HRMS): (compounds 1–22, Figures S34–S48)

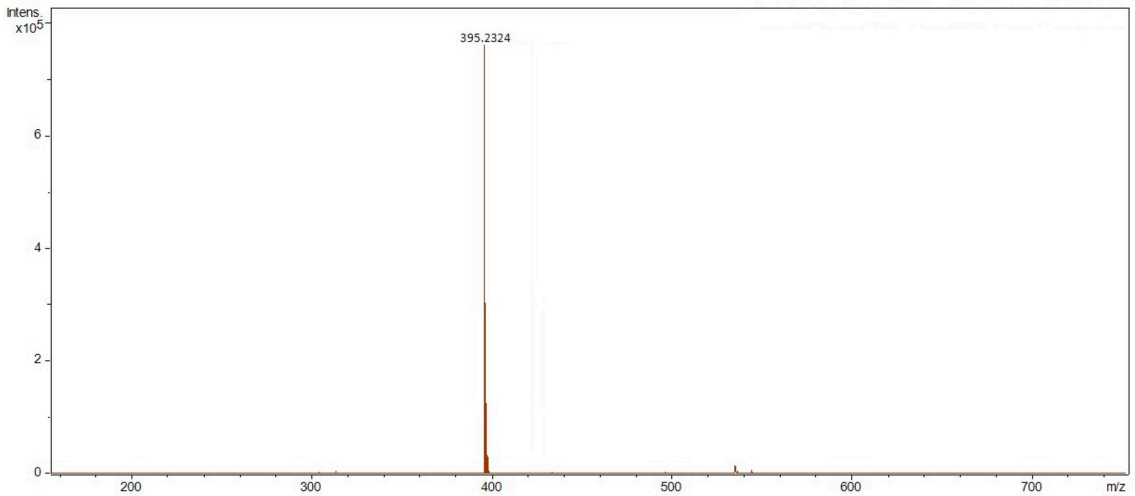

Figure S34.- High resolution mass spectrum (HRMS) of compound (**7**). The peak at m/z 395.2324

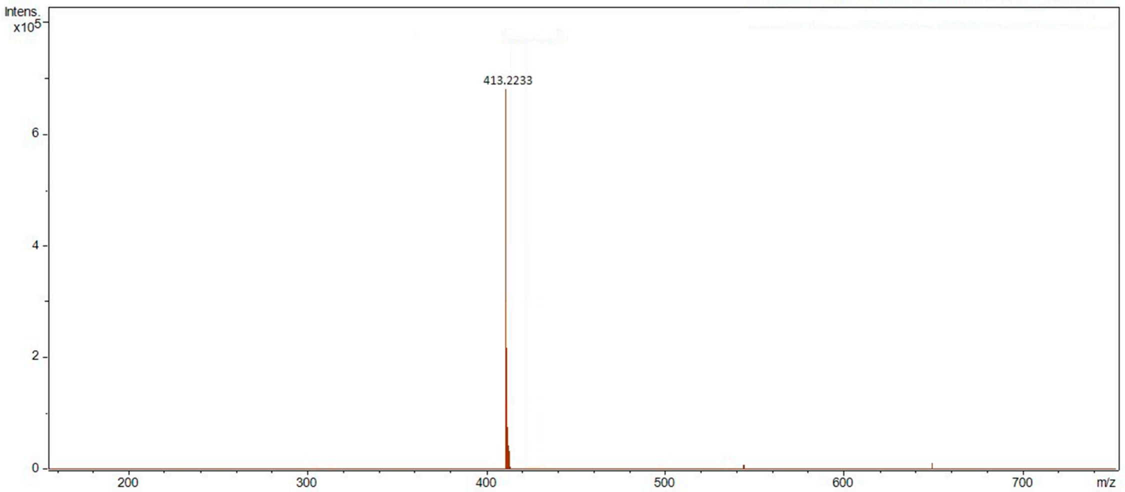

Figure S35.- High resolution mass spectrum (HRMS) of compound (**8**). The peak at m/z 413.2233.

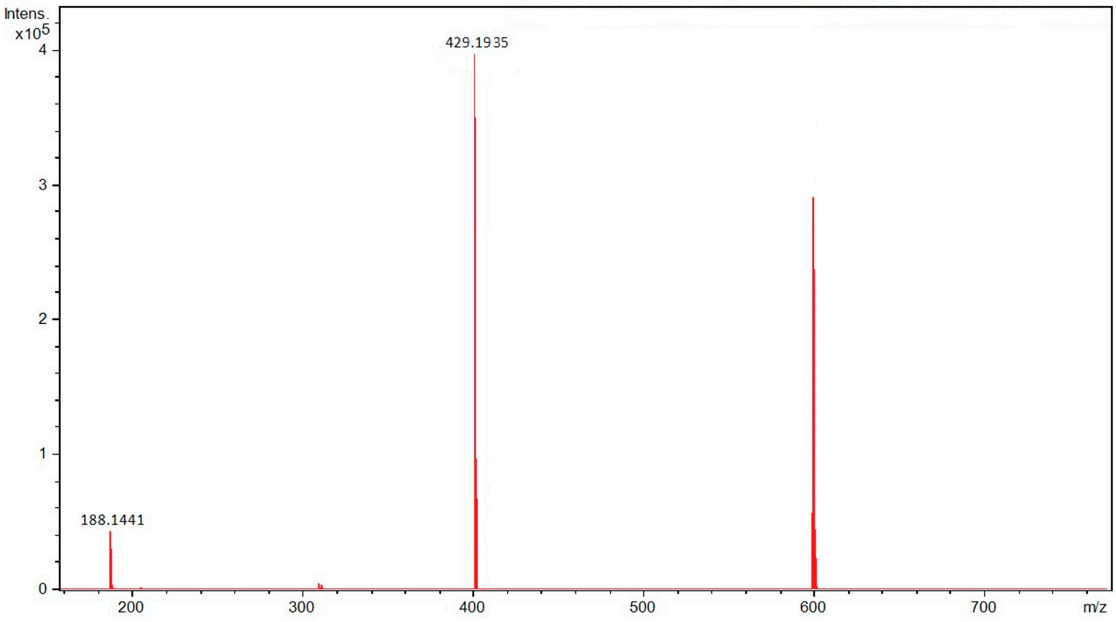

Figure S36.- High resolution mass spectrum (HRMS) of compound (**9**). The peak at m/z 429.1935.

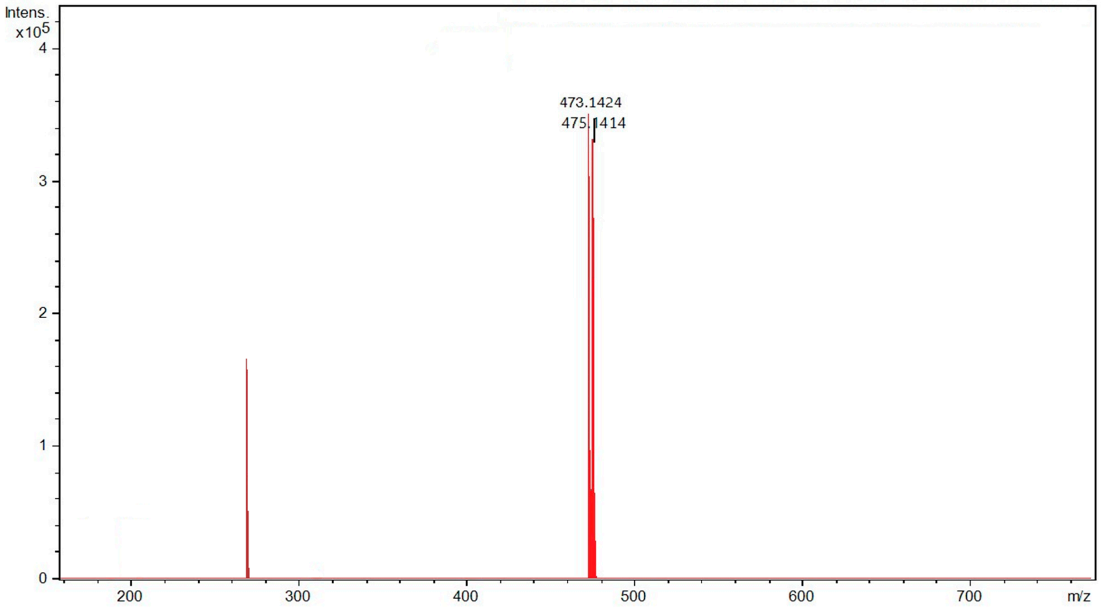

Figure S37.- High resolution mass spectrum (HRMS) of compound (**10**). The peak at $C_{24}H_{29}{}^{81}BrN_2O_3$ m/z 475.1414 and $C_{24}H_{29}{}^{79}BrN_2O_3$ m/z 473,1424.

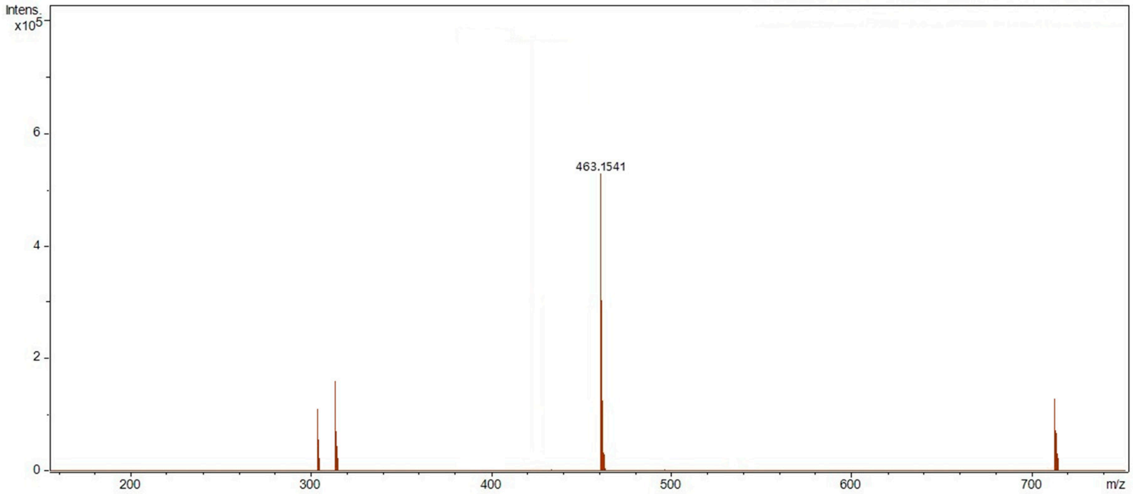

Figure S38.- High resolution mass spectrum (HRMS) of compound (**11**). The peak at m/z 463.1541.

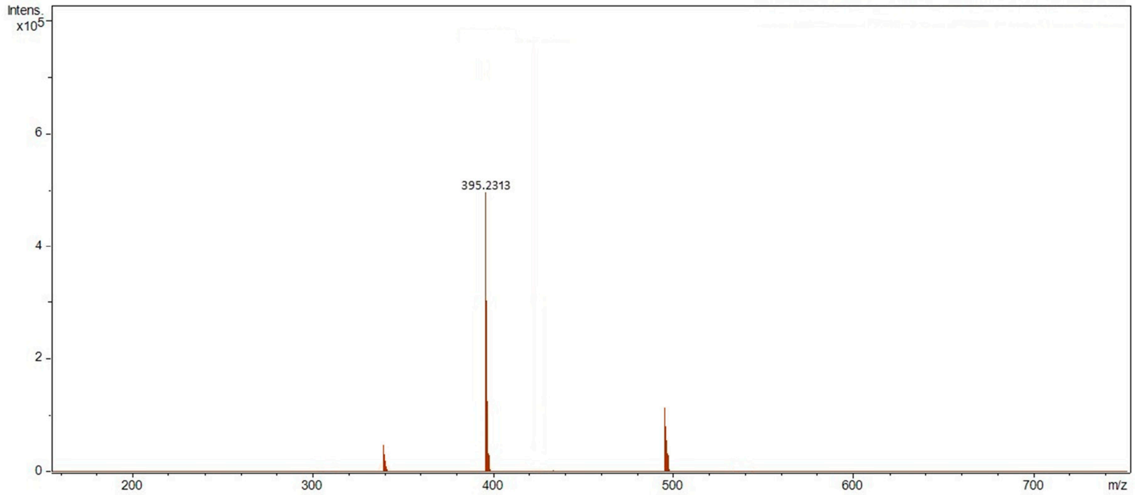

Figure S39.- High resolution mass spectrum (HRMS) of compound (**12**). The peak at m/z 395.2313.

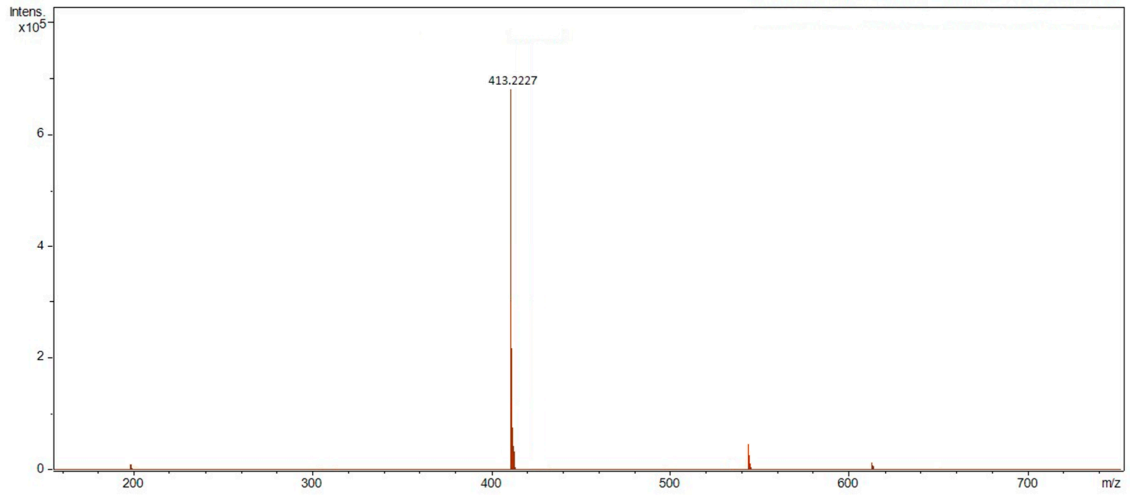

Figure S40.- High resolution mass spectrum (HRMS) of compound (**13**). The peak at m/z 413.2227.

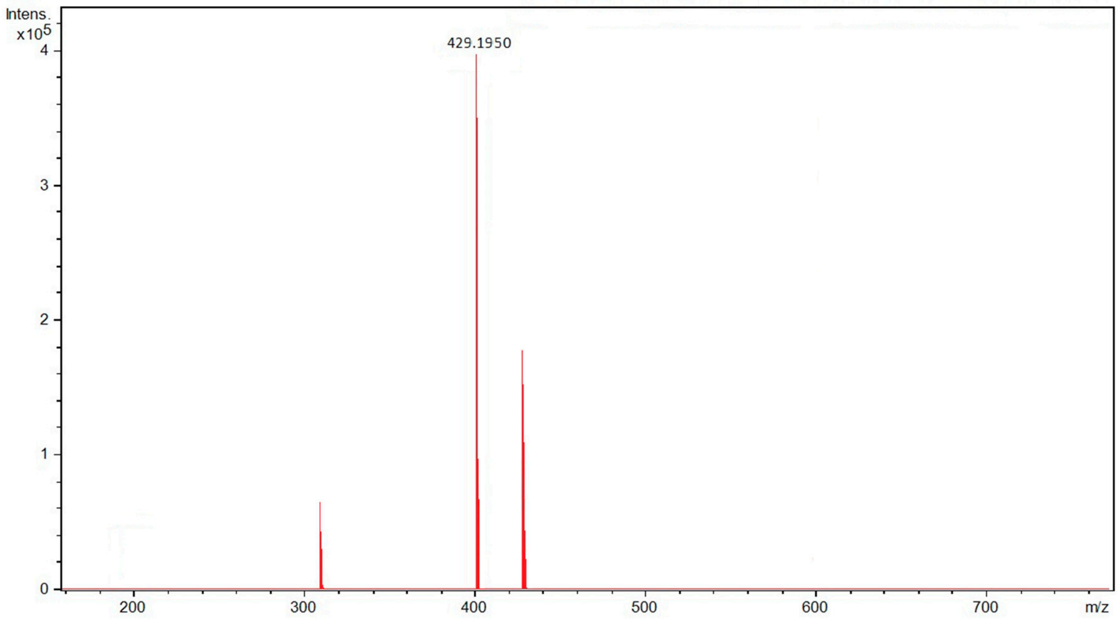

Figure S41.- High resolution mass spectrum (HRMS) of compound (**14**). The peak at m/z 429.1950.

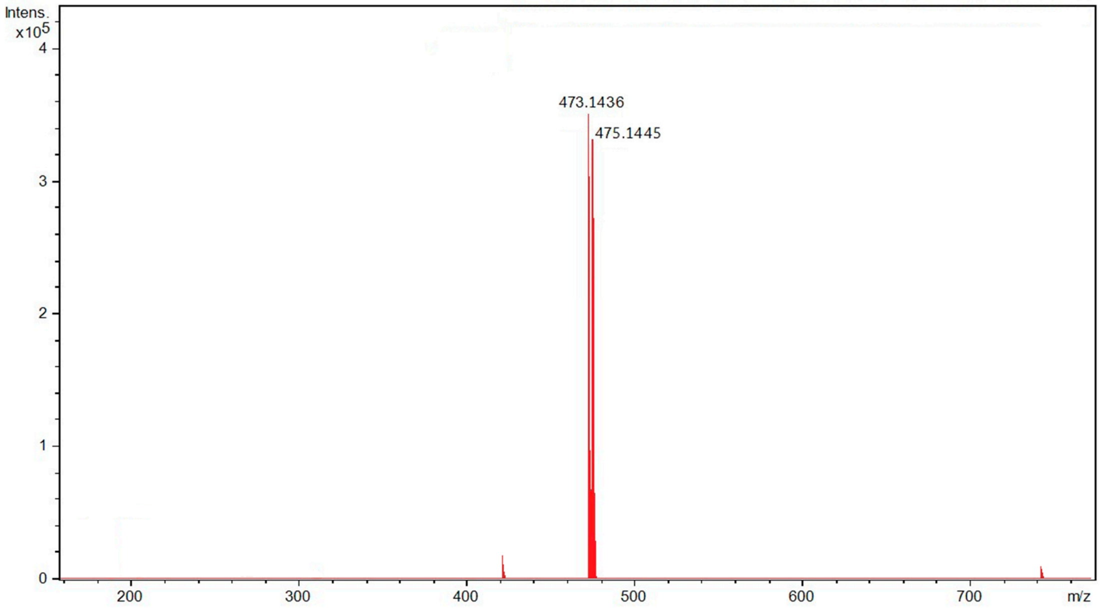

Figure S42.- High resolution mass spectrum (HRMS) of compound (**15**). The peak at $C_{24}H_{29}{}^{81}BrN_2O_3$ m/z 475.1436 and $C_{24}H_{29}{}^{79}BrN_2O_3$ m/z 473,1445.

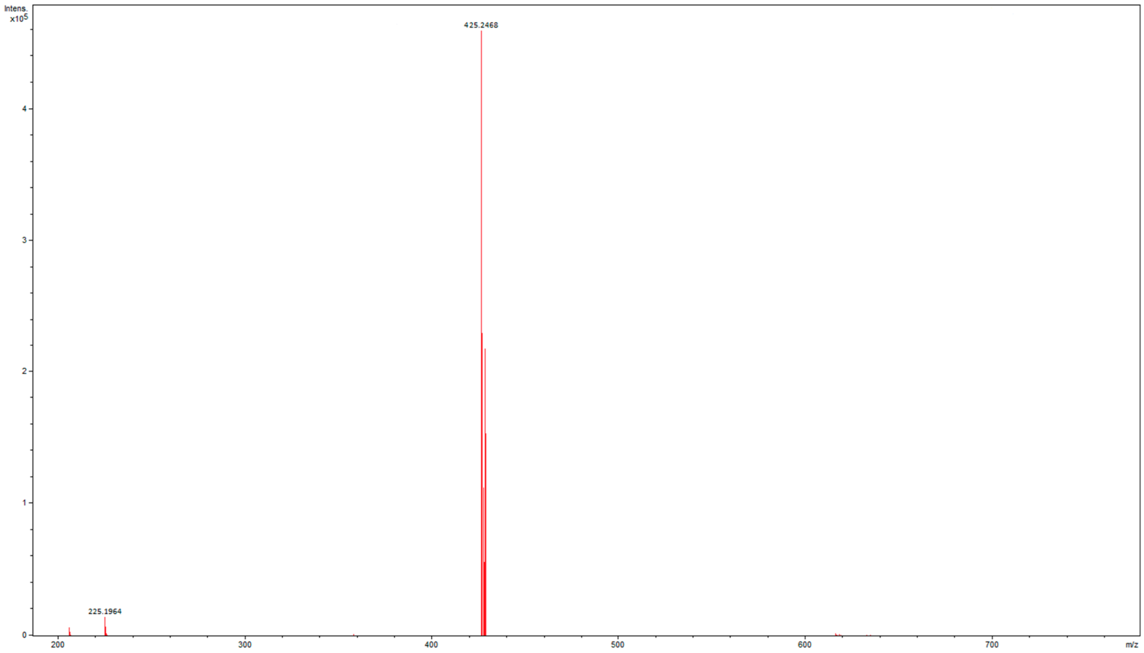

Figure S43.- High resolution mass spectrum (HRMS) of compound (**16**). The peak at m/z 425.2468.

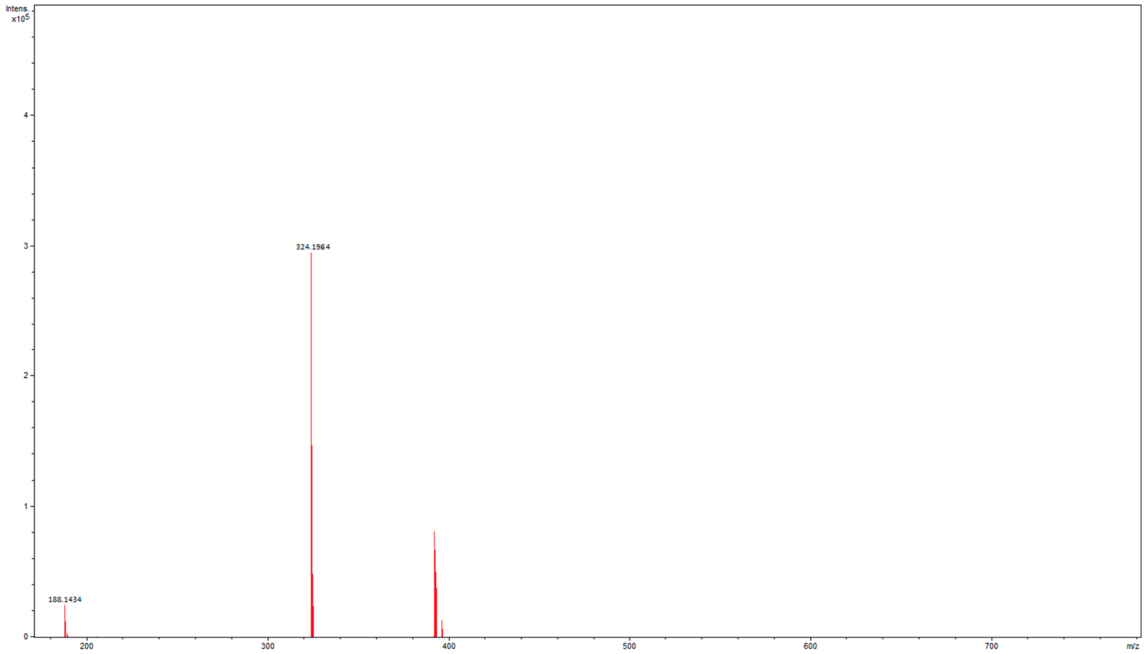

Figure S44.- High resolution mass spectrum (HRMS) of compound (**18**). The peak at m/z 324,1964.

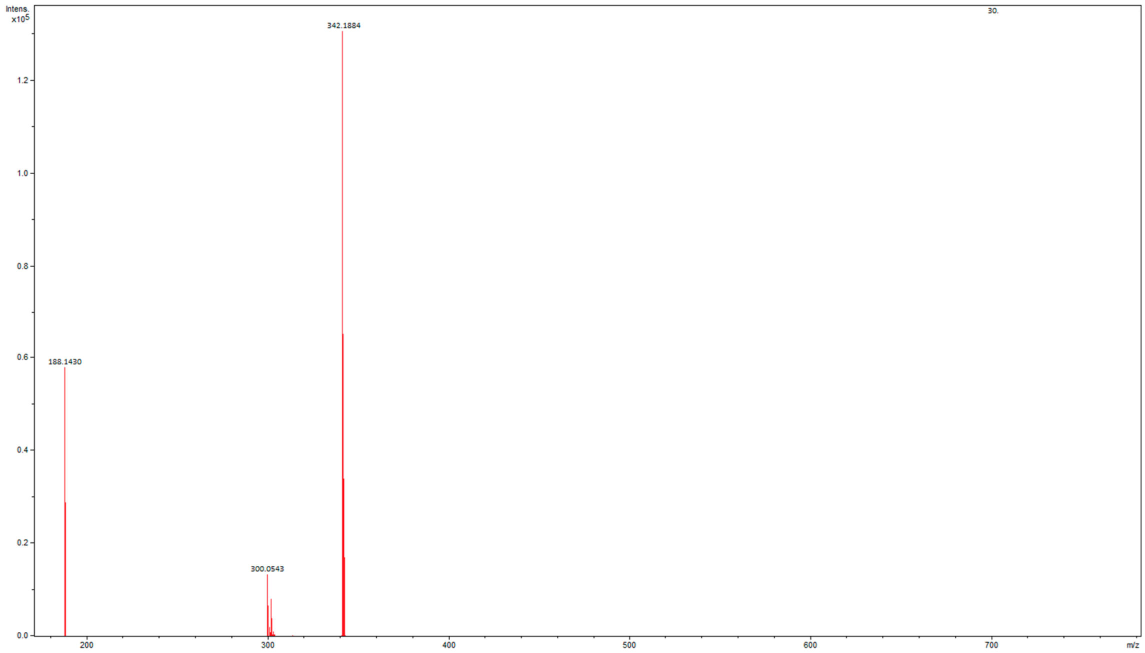

Figure S45.- High resolution mass spectrum (HRMS) of compound (**19**). The peak at m/z 342,1884.

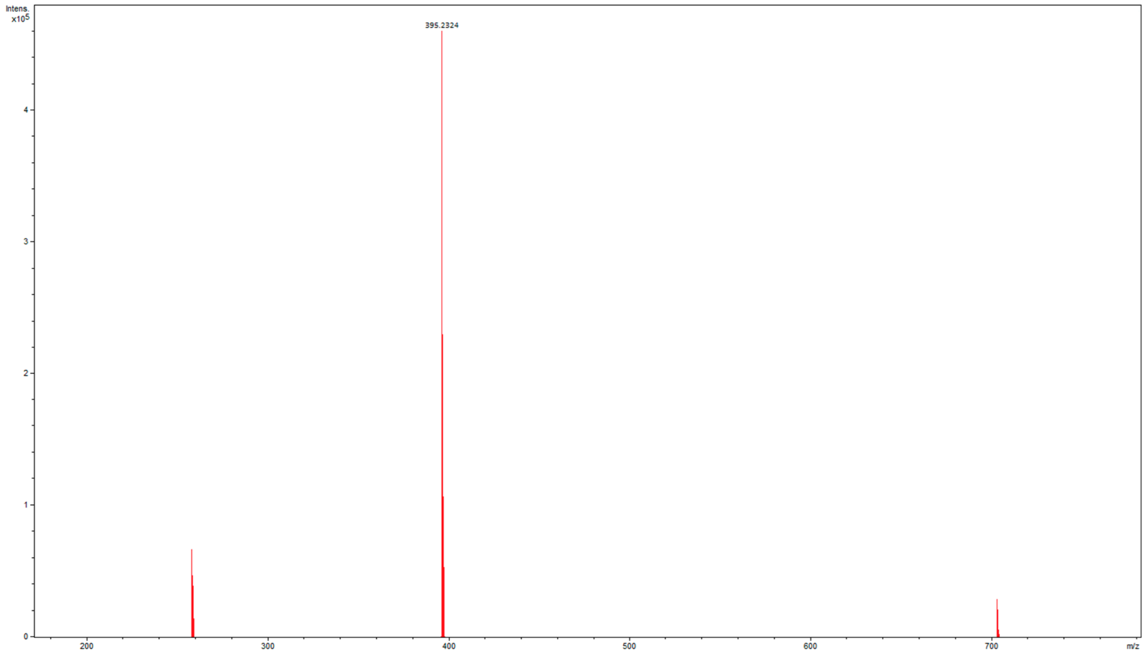

Figure S46.- High resolution mass spectrum (HRMS) of compound (**20**). The peak at m/z 395.2324.

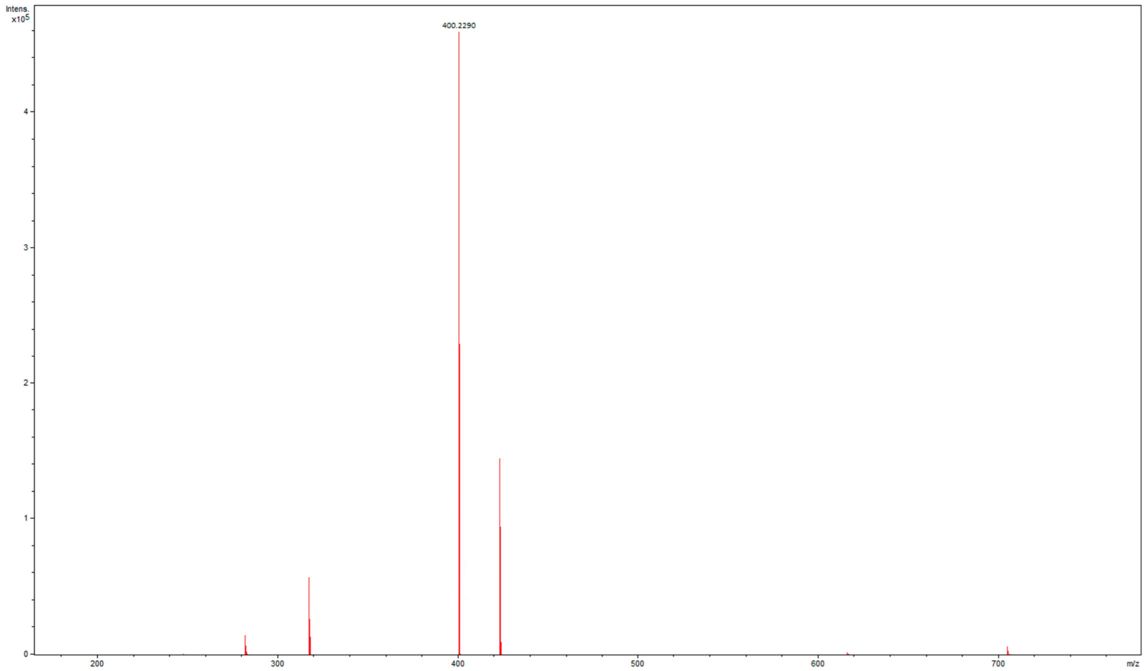

Figure S47.- High resolution mass spectrum (HRMS) of compound (**21**). The peak at m/z 400,2290.

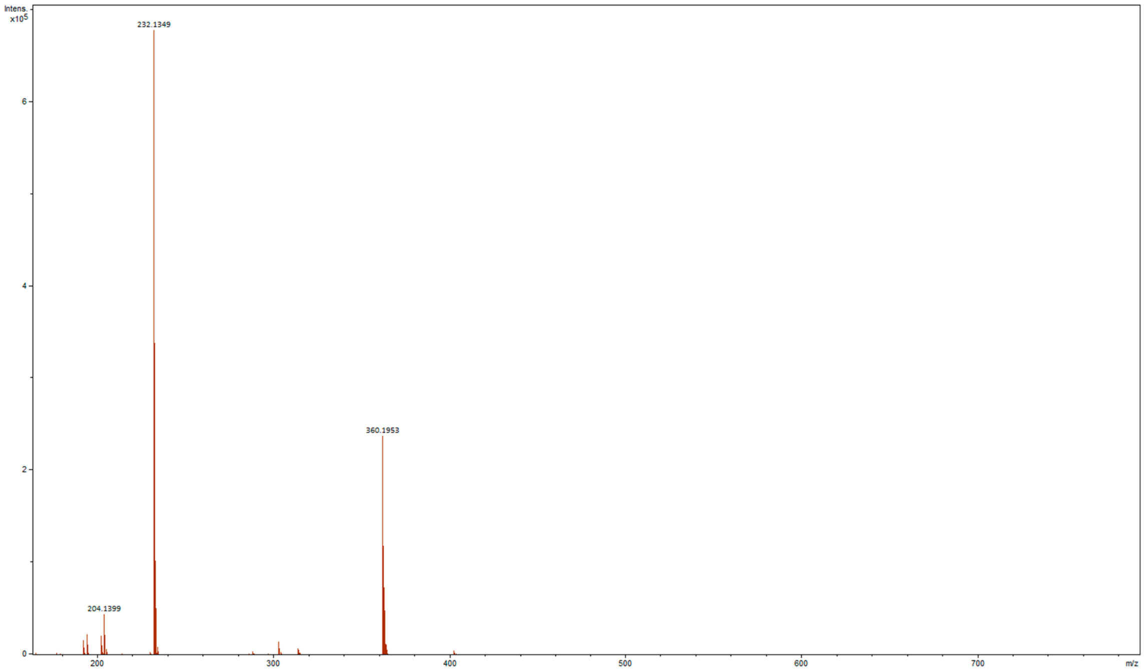

Figure S48.- High resolution mass spectrum (HRMS) of compound (**22**). The peak at m/z 360,1953.

2D - NMR HSQC and HMBC compounds 7 and 12.

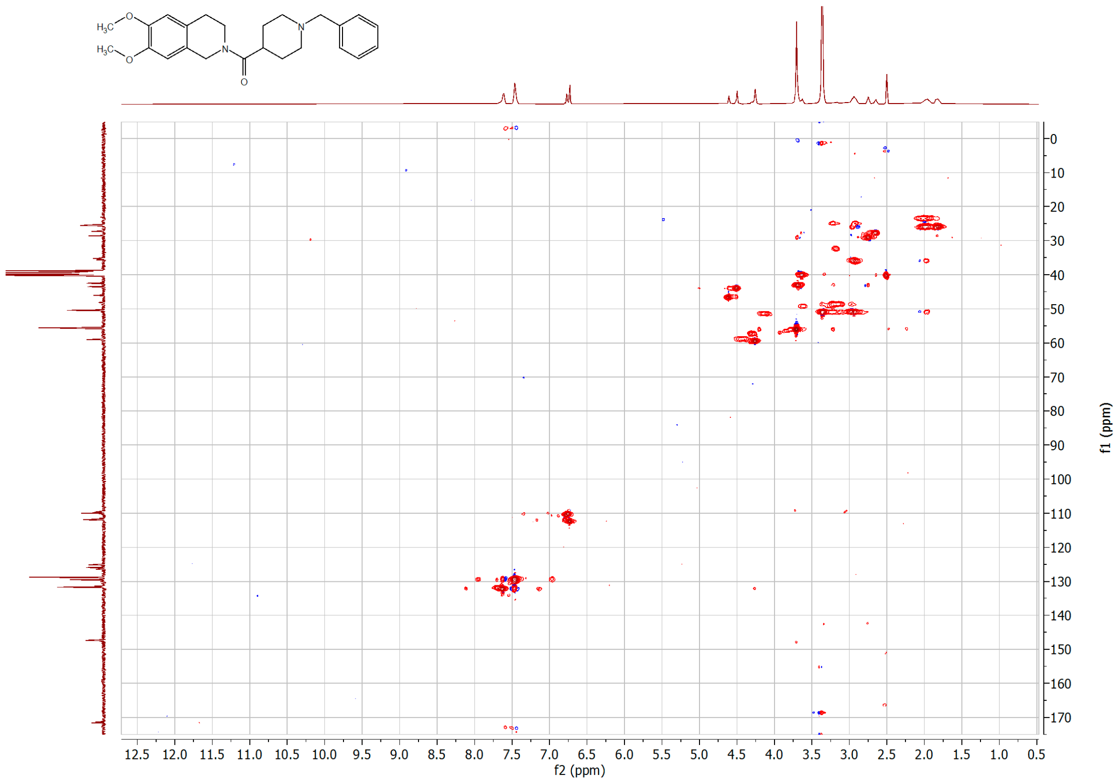

Figure S49.- 2D-HSQC of compound (**7**).

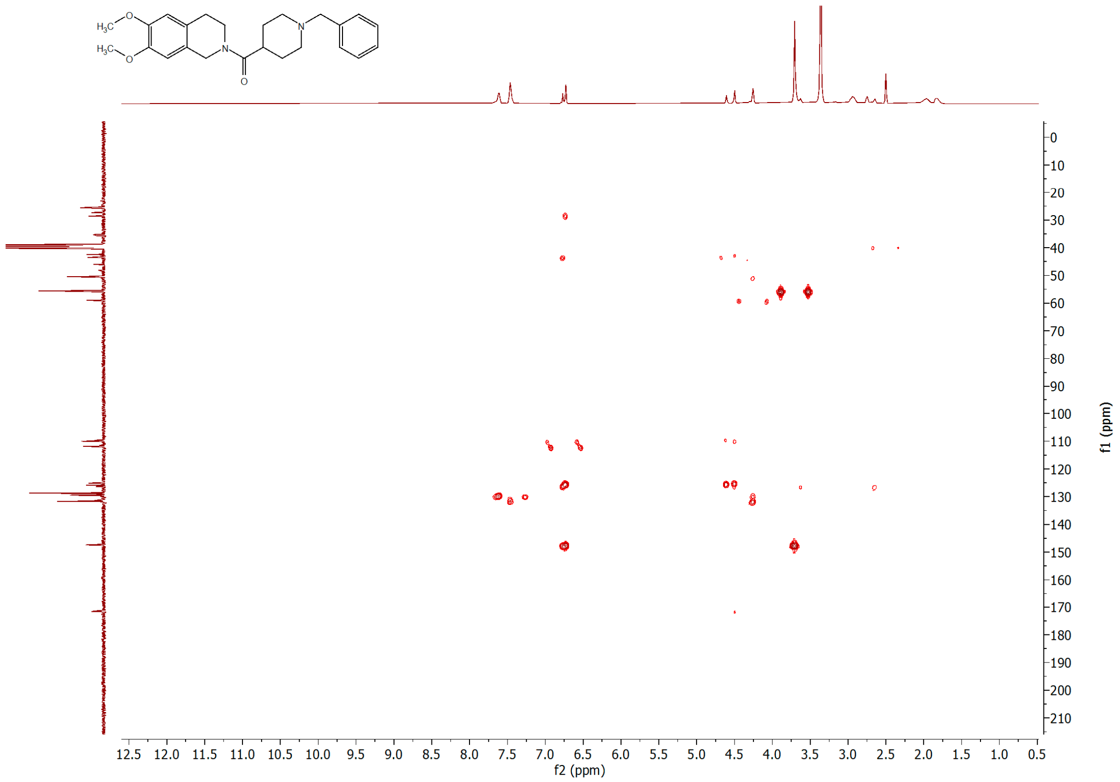

Figure S50.- 2D-HMBC of compound (**7**).

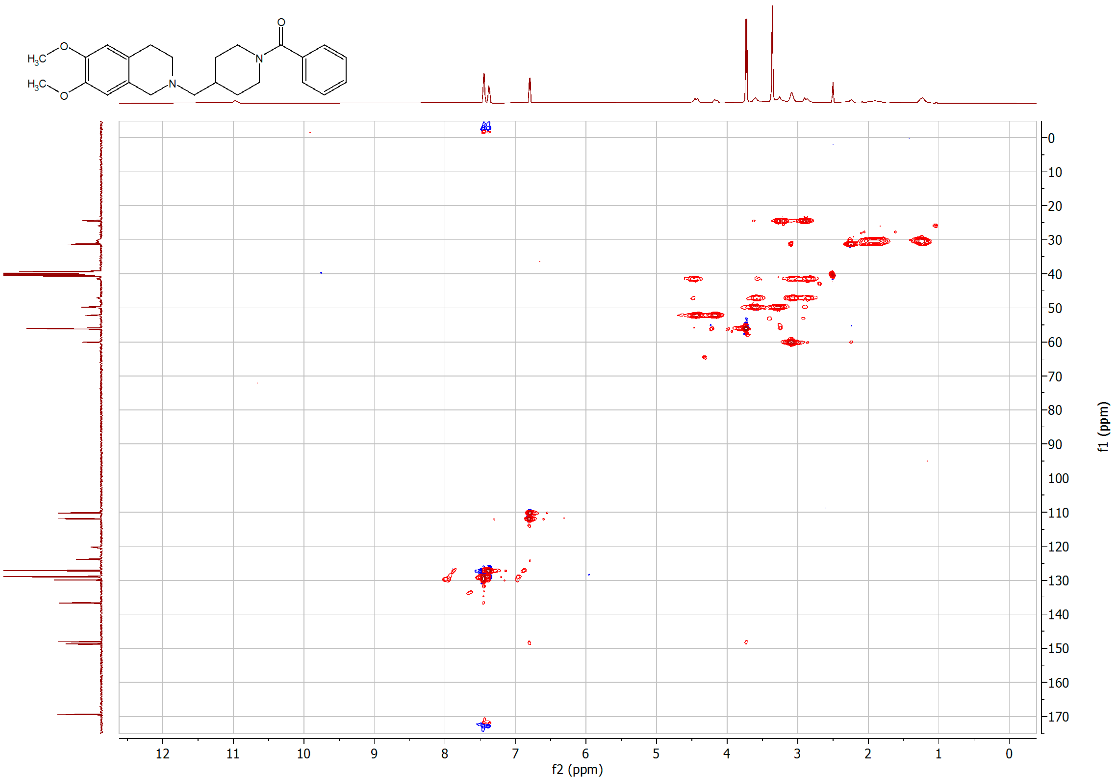

Figure S51.- 2D-HSQC of compound (**12**).

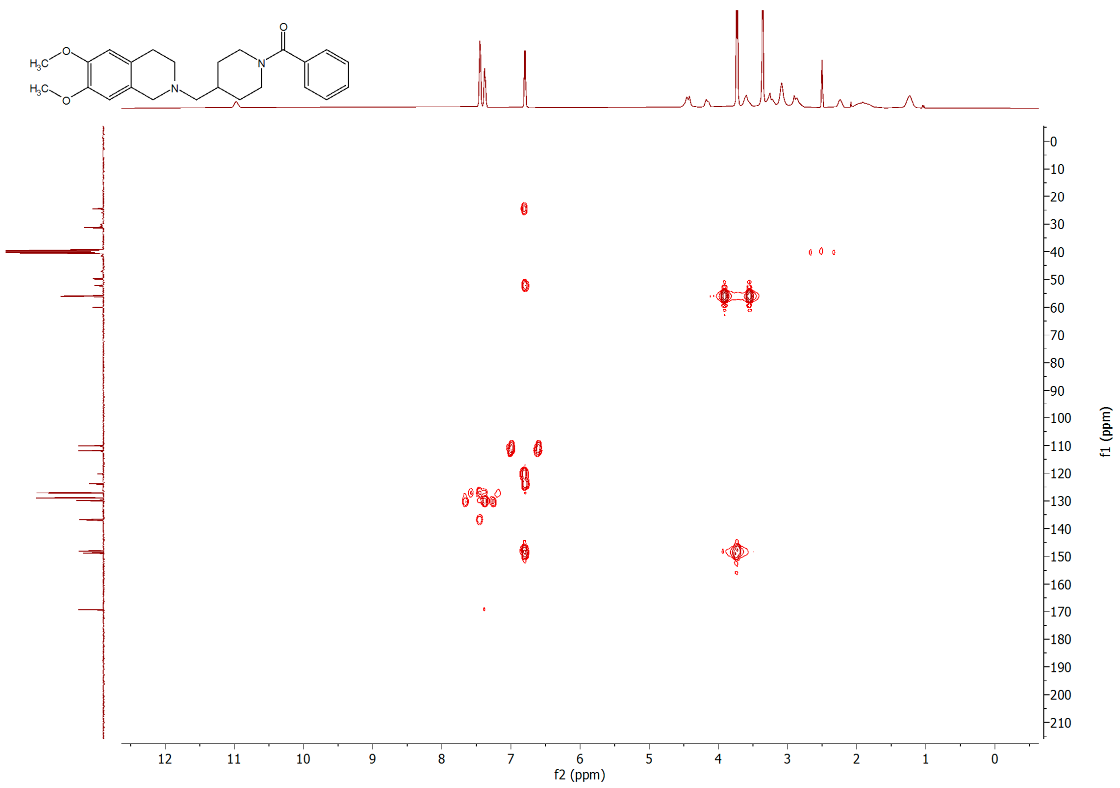

Figure S52.- 2D-HMBC of compound (**12**).

Biological assays - Concentration-Response Curves.

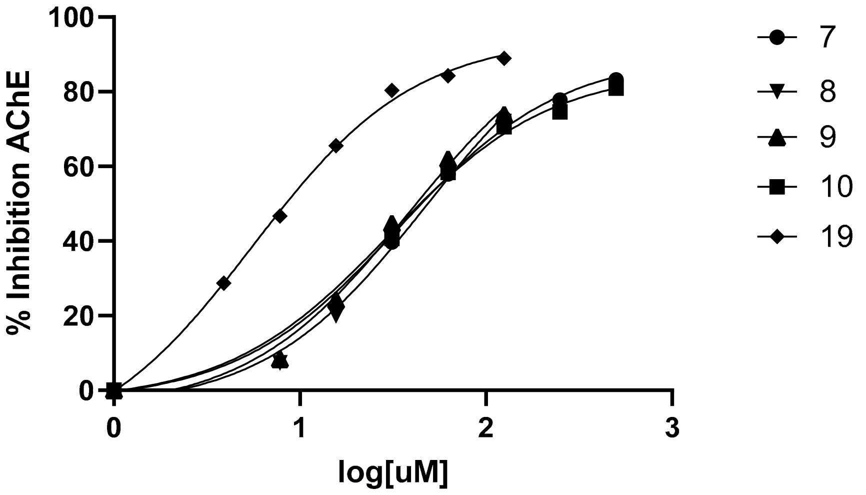

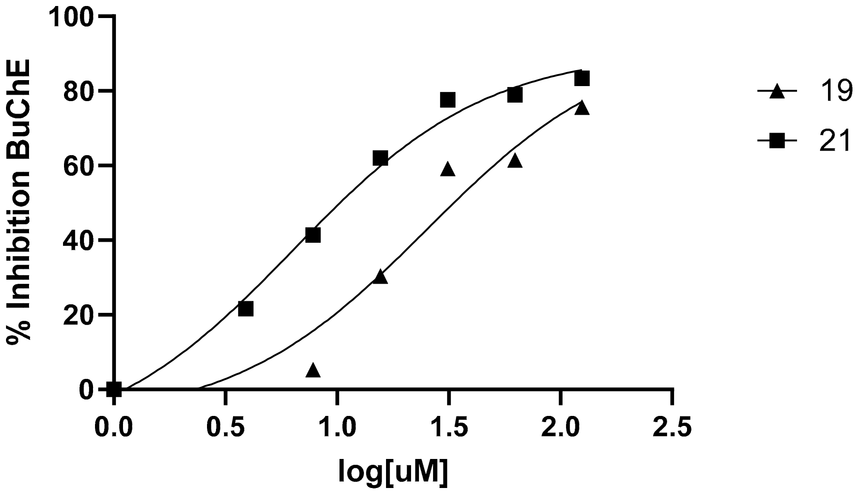

Figure S53.- Inhibitory activity (Compounds **7-10**) against AChE. Compound **19's** effect on inhibitory activity against AChE and BuChE and compound **21** effect on inhibitory activity against BuChR. Data points represent the mean ± SD of three experiments, each one carried out in triplicate.

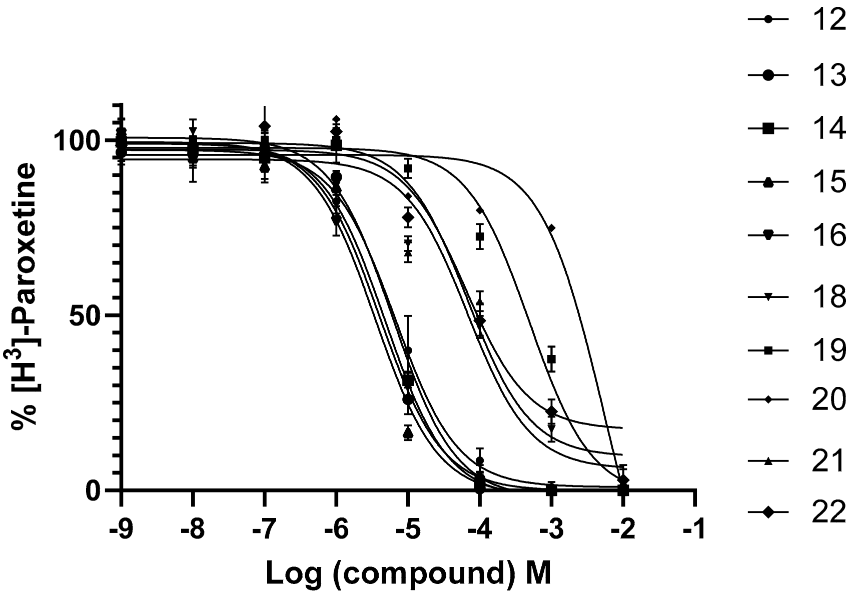

Figure S54 Compounds' effects on the binding of [3H]-paroxetine to h-SERT. Data points represent the mean ± SEM of three experiments, each one carried out in triplicate. The radioligand concentration in all displacement studies was 2 nM.
